# Development of the SIOPE DIPG network, registry and imaging repository: a collaborative effort to optimize research into a rare and lethal disease

**DOI:** 10.1007/s11060-016-2363-y

**Published:** 2017-01-21

**Authors:** Sophie E. M. Veldhuijzen van Zanten, Joshua Baugh, Brooklyn Chaney, Dennis De Jongh, Esther Sanchez Aliaga, Frederik Barkhof, Johan Noltes, Ruben De Wolf, Jet Van Dijk, Antonio Cannarozzo, Carin M. Damen-Korbijn, Jan A. Lieverst, Niclas Colditz, Marion Hoffmann, Monika Warmuth-Metz, Brigitte Bison, David T. W. Jones, Dominik Sturm, Gerrit H. Gielen, Chris Jones, Esther Hulleman, Raphael Calmon, David Castel, Pascale Varlet, Géraldine Giraud, Irene Slavc, Stefaan Van Gool, Sandra Jacobs, Filip Jadrijevic-Cvrlje, David Sumerauer, Karsten Nysom, Virve Pentikainen, Sanna-Maria Kivivuori, Pierre Leblond, Natasha Entz-Werle, Andre O. von Bueren, Antonis Kattamis, Darren R. Hargrave, Péter Hauser, Miklos Garami, Halldora K. Thorarinsdottir, Jane Pears, Lorenza Gandola, Giedre Rutkauskiene, Geert O. Janssens, Ingrid K. Torsvik, Marta Perek-Polnik, Maria J. Gil-da-Costa, Olga Zheludkova, Liudmila Shats, Ladislav Deak, Lidija Kitanovski, Ofelia Cruz, Andres Morales La Madrid, Stefan Holm, Nicolas Gerber, Rejin Kebudi, Richard Grundy, Enrique Lopez-Aguilar, Marta Zapata-Tarres, John Emmerik, Tim Hayden, Simon Bailey, Veronica Biassoni, Maura Massimino, Jacques Grill, William P. Vandertop, Gertjan J. L. Kaspers, Maryam Fouladi, Christof M. Kramm, Dannis G. van Vuurden, on behalf of the members of the SIOPE DIPG Network

**Affiliations:** 1 Department of Paediatrics, Division of Oncology/Haematology, VU University Medical Center, Amsterdam, The Netherlands; 2 Department of Pediatrics, Cancer and Blood Diseases Institute, Cincinnati Children’s Hospital Medical Center, Cincinnati, USA; 3 2TCI B.V., Breda, The Netherlands; 4 Department of Radiology and Nuclear Medicine, VU University Medical Center, Amsterdam, The Netherlands; 5 KPMG, Amstelveen, The Netherlands; 6 Yellow Research B.V., Amsterdam, The Netherlands; 7 Trasferimento Tecnologico (TTO), Fondazione IRCCS Istituto Nazionale dei Tumori, Milano, Italy; 8 Dutch Childhood Oncology Group (DCOG), The Hague, The Netherlands; 9 Division of Pediatric Hematology and Oncology, Department of Child and Adolescent Health, University of Göttingen, Göttingen, Germany; 10 Division of Pediatric Neurooncology, German Cancer Research Center (DKFZ) Heidelberg, Heidelberg, Germany; 11 Institute of Neuropathology, University of Bonn Medical Center, Bonn, Germany; 12 Divisions of Molecular Pathology and Cancer Therapeutics, The Institute of Cancer Research, Sutton, UK; 13 Neuro-oncology Research Group, VU University Medical Center, Amsterdam, The Netherlands; 14 Université Paris-Descartes, Hôpital Necker-Enfants malades, Paris, France; 15 UMR 8203 CNRS, Gustave Roussy, Paris-Saclay University, Villejuif, France; 16 Service de Neuropathologie, Hôpital Sainte-Anne, Paris, France; 17 Institut Gustave Roussy, Villejuif, France; 18 Department of Pediatrics and Adolescent Medicine, Medical University of Vienna, Vienna, Austria; 19 Immuno-oncology Centre Köln, Cologne, Germany; 20 Department of Pediatric Hematology and Oncology, University Hospitals Leuven, Leuven, Belgium; 21 Department of Microbiology and Immunology, KU Leuven, Leuven, Belgium; 22 Department of Oncology and Hematology, Children’s Hospital Zagreb, Zagreb, Croatia; 23 Department of Pediatric Hematology and Oncology, 2nd Faculty of Medicine, University Hospital Motol, Charles University, Prague, Czech Republic; 24 Pediatrics and Adolescent Medicine, University Hospital Rigshospitalet, Copenhagen, Denmark; 25 Division of Hematology-Oncology and Stem Cell Transplantation, Children’s Hospital, Helsinki University Hospital, Helsinki, Finland; 26 Helsinki University Hospital (HUH) and HUH Children and Adolescents, Helsinki, Finland; 27 Pediatric Oncology Unit, Oscar Lambret Comprehensive Cancer Center, Lille, France; 28 Service de Pédiatrie Onco-Hématologie, CHRU Hautepierre Strasbourg, Strasbourg, France; 29 Pediatric Oncology and Hematology, Department of Pediatrics, University Hospital of Geneva, Genève, Switzerland; 30 First Department of Pediatrics, National and Kapodistrian University of Athens, ‘Aghia Sofia’ Children’s Hospital, Athens, Greece; 31 Paediatric Oncology Unit, Great Ormond Street Hospital (GOSH), London, UK; 32 Second Department of Pediatrics, Semmelweis University, Budapest, Hungary; 33 Pediatric Hematology-Oncology, The Children’s Hospital, Reykjavik, Iceland; 34 Department of Paediatric Oncology, Our Lady’s Children’s Hospital, Crumlin, Dublin, Ireland; 35 Pediatric Radiotherapy Unit, Department of Diagnostic Radiology and Radiotherapy, Fondazione IRCCS Istituto Nazionale dei Tumori, Milano, Italy; 36 Department of Pediatric Oncology and Hematology, Hospital of Lithuanian University of Health Sciences Kaunas Clinic, Kaunas, Lithuania; 37 Department of Radiation Oncology, University Medical Center Utrecht and Princess Maxima Center for Pediatric Oncology, Utrecht, The Netherlands; 38 Department of Pediatrics, Division of Oncology/Hematology, Haukeland University Hospital, Mons, Norway; 39 Department of Oncology, The Children’s Memorial Health Institute, Warsaw, Poland; 40 Pediatric Hemathology and Oncology Division, University Hospital S. João Alameda Hernani Monteiro, Porto, Portugal; 41 Department of Neuro-Oncology, Russian Scientific Center of Radiology, Moscow, Russia; 42 Department of Children Oncology and Haematology, City Clinical Hospital #31 of Saint-Petersburg, St.-Petersburg, Russian Federation; 43 Department of Pediatric Oncology/ Haematology, Children University Hospital, Kosice, Slovakia; 44 Department of Pediatrics, Division of Haematooncology, University Medical Center Ljubljana, Ljubljana, Slovenia; 45 Department of Pediatric Hematology and Oncology, Hospital Sant Joan de Déu, Barcelona, Spain; 46 Department of Pediatric Hematology and Oncology, Department of Woman and Child Health, Karolinska University Hospital, Stockholm, Sweden; 47 Department of Oncology, University Children’s Hospital of Zurich, Zurich, Switzerland; 48 Cerrahpasa Medical Faculty & Oncology Institute, Pediatric Hematology-Oncology, Istanbul University, Istanbul, Turkey; 49 Children’s Brain Tumour Research Centre, The Medical School, University of Nottingham, Nottingham, UK; 50 Jefatura de Servicio de Oncología, Hospital de Pediatría, Centro Médico Nacional Siglo XXI, Instituto Mexicano del Seguro Social, Distrito Federal, Mexico; 51 Division of Pediatric Oncology, Hospital Infantil de Mexico Federico Gomez, Mexico City, Mexico; 52 Stichting Semmy, Weesp, The Netherlands; 53 The DIPG Collaborative, Cincinnati, USA; 54 Great North Childrens Hospital, Victoria Wing, Royal Victoria Infirmary, Newcastle upon Tyne, UK; 55 Pediatric Oncology Unit, Fondazione IRCCS Istituto Nazionale dei Tumori, Milano, Italy; 56 Neurosurgical Center Amsterdam, Academic Medical Center and VU University Medical Center, Amsterdam, The Netherlands; 57 Academy of Princess Máxima Center for Pediatric Oncology, Lundlaan 6, 3584 EA Utrecht, The Netherlands; 58 Institutes of Neurology and Healthcare Engineering, UCL, London, UK

**Keywords:** Diffuse intrinsic pontine glioma (DIPG), Collaboration, International research-infrastructure, SIOPE DIPG network, SIOPE DIPG registry

## Abstract

Diffuse intrinsic pontine glioma (DIPG) is a rare and deadly childhood malignancy. After 40 years of mostly single-center, often non-randomized trials with variable patient inclusions, there has been no improvement in survival. It is therefore time for international collaboration in DIPG research, to provide new hope for children, parents and medical professionals fighting DIPG. In a first step towards collaboration, in 2011, a network of biologists and clinicians working in the field of DIPG was established within the European Society for Paediatric Oncology (SIOPE) Brain Tumour Group: the SIOPE DIPG Network. By bringing together biomedical professionals and parents as patient representatives, several collaborative DIPG-related projects have been realized. With help from experts in the fields of information technology, and legal advisors, an international, web-based comprehensive database was developed, The SIOPE DIPG Registry and Imaging Repository, to centrally collect data of DIPG patients. As for April 2016, clinical data as well as MR-scans of 694 patients have been entered into the SIOPE DIPG Registry/Imaging Repository. The median progression free survival is 6.0 months (95% Confidence Interval (CI) 5.6–6.4 months) and the median overall survival is 11.0 months (95% CI 10.5–11.5 months). At two and five years post-diagnosis, 10 and 2% of patients are alive, respectively. The establishment of the SIOPE DIPG Network and SIOPE DIPG Registry means a paradigm shift towards collaborative research into DIPG. This is seen as an essential first step towards understanding the disease, improving care and (ultimately) cure for children with DIPG.

**Electronic supplementary material:**

The online version of this article (doi:10.1007/s11060-016-2363-y) contains supplementary material, which is available to authorized users.

## Introduction

Diffuse intrinsic pontine glioma (DIPG) is a pediatric brain cancer for which there is no curative treatment yet. Despite multiple clinical trials studying (combinations of) cytotoxic chemotherapy, including novel agents, the median overall survival of 9 months has not improved over the past decades [1, 2]. Although major advances have been accomplished in knowledge on the biological background of the disease by discovery of a high prevalence of specific mutations in genes encoding for histone 3.1 and 3.3, ACVR1 and P53 [3–9], much is yet to be learned on the mechanisms that contribute to treatment resistance. Research on the DIPG patient population, however, is hampered because integrative, large scale clinical, radiological and biological data are lacking.

There are several factors that contribute to the scarcity of data. First, DIPG is an orphan disease with a yearly incidence of 2.32 per 1,000,000 residents aged 0–20 years [10]. Second, DIPGs are diagnosed clinically, based on typical MR-imaging findings [11], in combination with a classic triad of neurological symptoms [12]. Biopsy procedures to obtain tumor material have long been considered dangerous and not contributing to the diagnosis, treatment approach or survival outcome [13]. Fortunately, recent years have seen an emergence of studies that include biopsies, however, the discovery of new mutations have caused an on-going debate about the actual definition of the disease itself [9, 14]. This is exemplified by the recently published new WHO classification of central nervous system tumors, that has reclassified DIPG to the category of WHO grade IV diffuse midline gliomas with histone mutations [15]. Inconsistent definition of DIPG has hampered in- and exclusion or response criteria for clinical trials, which resulted in a great variety of mostly incomparable clinical trials, many of which are single-center, single-arm studies with only few patients enrolled [10].

Collaboration and data sharing are promising strategies for tackling rare diseases, by facilitating uniform and hypothesis-driven research [16]. To overcome the current lack of data and improve the integration, speed, quality, and coherence of research, we aimed to (1) create a DIPG research-infrastructure consortium, and (2) initiate collaborative collection of comprehensive data on DIPG patients. This paper describes the methodology of the set-up of an international research network infrastructure, the SIOPE DIPG Network and SIOPE DIPG Registry, including legal and IT aspects, as well as preliminary patient inclusion data.

## Materials and methods

### The establishment of a research-infrastructure consortium

In January 2011, in a DIPG meeting organized by the Semmy Foundation in Amsterdam, the SIOPE DIPG Network was established as a sub-committee of the high-grade glioma (HGG) working group of International Society of Paediatric Oncology Europe (SIOPE). The SIOPE DIPG Network is a collaboration of pediatric oncologists, neurologists, neurosurgeons, radiotherapists, radiologists, pathologists, molecular biologists, psychologists and others motivated to carry out excellent clinical and biological research in the field of DIPG. Initially started as a European network, it has extended to colleagues from all over the world, with participants from Russia, Turkey and Mexico.

The SIOPE DIPG Network is comprised of (i) an executive committee, (ii) a group of scientific advisors, (iii) National Coordinators (NCs) and (iv) members. The Executive Committee (i) manages and controls the DIPG Network, and abides by and enforces the mission and the core values of the Network. Scientific Advisors (ii) are individuals with expertise in areas such as: biostatistics and biometry, medical ethics and health policy, basic science research, translational research, (neuro)psychology, neuroimaging, or other areas not mentioned. Scientific Advisors are consulted to advise the Executive Committee in matters of development and implementation of research protocols including ideas for innovative studies that could be executed using the Network. NCs (iii) are those DIPG Network members that coordinate collaboration between the SIOPE DIPG Network and biologists and clinicians in their countries. NCs identify and select hospitals and scientific experts in their countries, that are involved in the treatment of DIPG patients and that potentially may join the DIPG Network. DIPG Network members (iv) participate in research projects initiated by the DIPG Network following the principles of Good Clinical Practice. Potential members need to be approved by the Executive Committee before subscription to the DIPG Network. Network members are free to decide on whether they wish to participate in a research project on a case-by-case basis and at their sole discretion.

The mission of the SIOPE DIPG Network is to serve as a research-infrastructure for the design and execution of high quality, international multicenter laboratory and clinical studies, intended to enhance the understanding of DIPG and to improve outcome of patients suffering from DIPG. The mission, aims, core values and structure of the SIOPE DIPG Network are described in the SIOPE DIPG Network Bylaws (see Legal aspects).

### Collaborative collection of comprehensive data

**Fig. 1:**
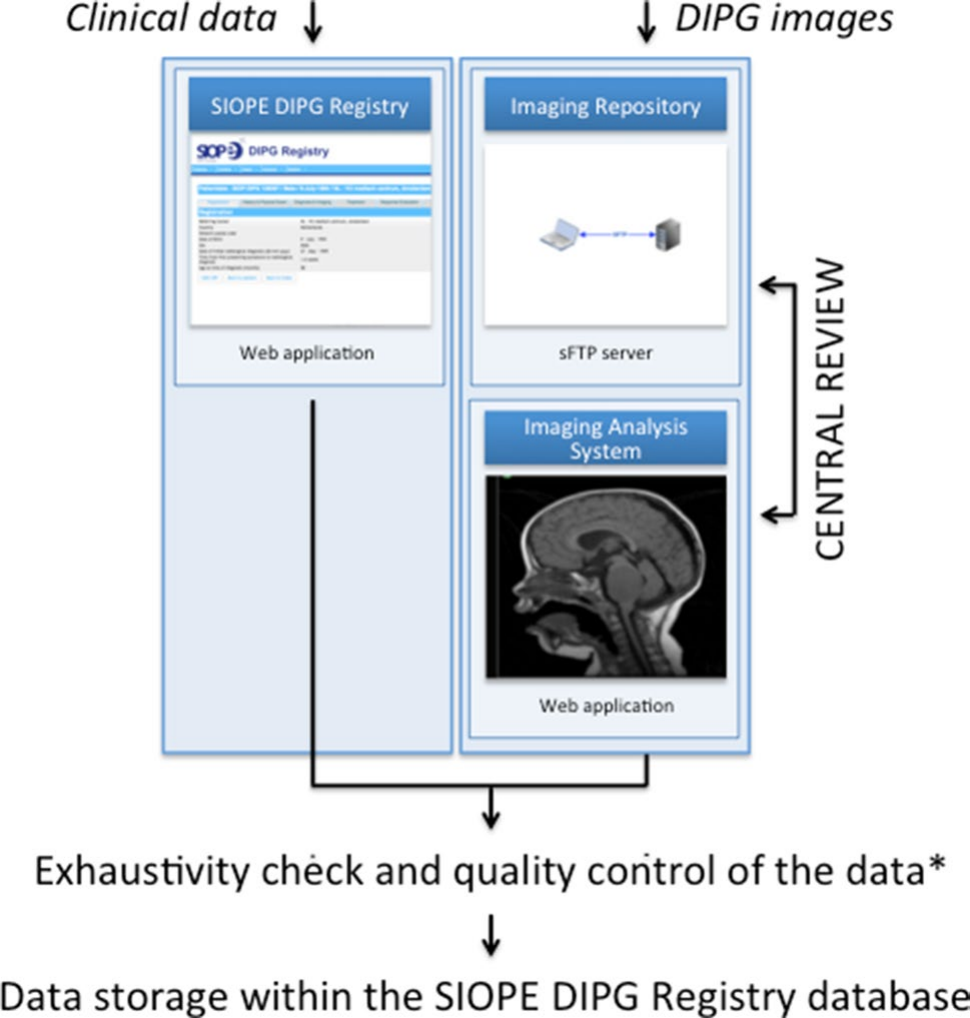
Organizational chart of the SIOPE DIPG registry and imaging repository. For details on the quality control process please see Supplementary Fig. 1

The establishment of a DIPG registry was set as first project of the Network, with the purpose to include clinical, biological and centrally reviewed radiology data of patients with DIPG, both in- and outside clinical trials. The SIOPE DIPG Registry is composed of an online web application and database for clinical data, and an Imaging Repository for radiological data (Fig. 1).

**Fig. 2:**
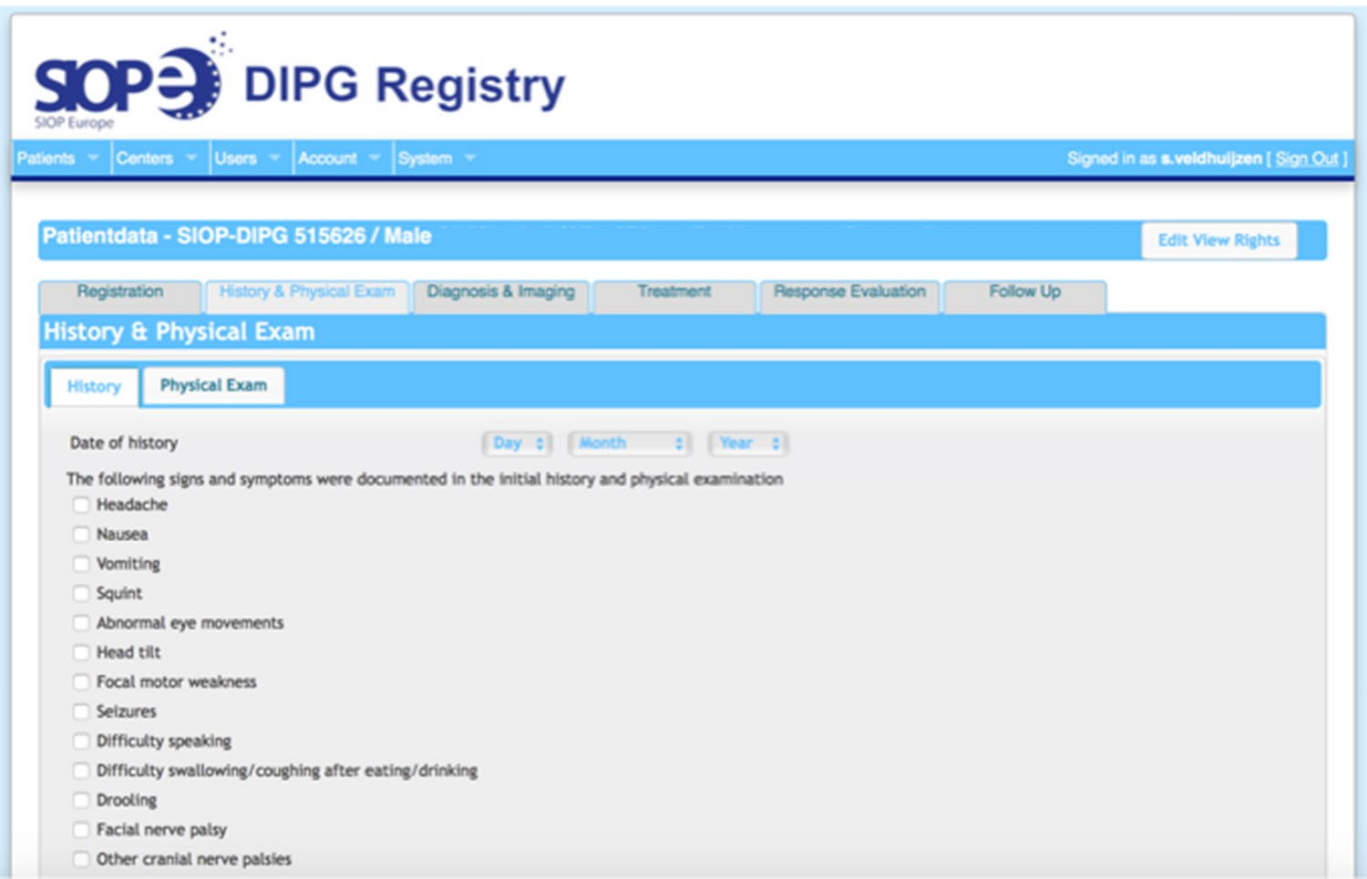
Screenshot of the SIOPE DIPG Registry showing the electronic case report forms (e-CRFs). The* open tab* represents the e-CRF for history and physical exam

In parallel, an International DIPG Registry was initiated and developed, which includes patient data from the USA, Canada, Australia and New Zealand. To allow for the inclusion of uniform data, standardized electronic Case Report Forms (e-CRFs; Fig. 2) were developed by the SIOPE DIPG Network, in coordination with colleagues from the International DIPG Registry. The online e-CRFs collect data on demographics, medical history and physical exam at time of diagnosis together with the results from radiological and pathological review by the local hospital, treatment data (including radiotherapy, chemotherapy, surgery and supportive care such as steroids), data on clinical and radiological follow up, and last known status of the patient (see Data Entry Manual; Supplementary material 1).

In parallel to the clinical data, anonymized MRI-scans scans are uploaded via a secure FTP server or sent on CDs. De-identification/ pseudonymization, according to the country’s law, is performed either in the referring center, during upload or at the time of receipt. When fully anonymized, these images are uploaded into the SIOPE DIPG Imaging Repository (Fig. 1). Expert neuroradiologists are brought together in a central neuroradiology panel. This panel has access to view assigned images from the Imaging Repository for blinded central review of submitted cases.

#### Eligibility criteria

The criteria for patient inclusion in the SIOPE DIPG Registry are: (i) patients with DIPG, or with focal Pontine Glioma (fPG), defined as a T1-weighted hypointense and T2-weighted hyperintense tumor with at least 50% involvement of the pons (DIPG) or less than 50% involvement of the pons (fPG) on T2, and as confirmed by expert neuroradiologists via the central radiology review procedure described above (ii) age at diagnosis between 0 and 21 years, and (iii) written informed consent in case of prospective registration. Furthermore, in order to enable validation of the diagnosis following the current guideline, a minimum of diagnostic criteria is required i.e. clinical and radiological data (MRI scans) to be shared in the registry and, if available, pathology data.

#### Ethical considerations

The SIOPE DIPG Registry is conducted according to the principles of the Declaration of Helsinki. No personal identifiers, besides date of birth, are included in the e-CRFs. If in a certain country this is not allowed, age at diagnosis is submitted instead. All patients are assigned a unique SIOPE DIPG Registry number. Per member site, a separate list, kept under a special password, connects the DIPG Registry number with the personal identifiers. Access to this list is restricted to a local coordinator at each site.

In most participating countries informed consent is not mandatory for retrospective registration of (mostly deceased) patients. If required a consent form is sent to parents and signed. Prospective registration of living patients requires an informed consent procedure. National coordinators are responsible for the translation of the standardized informed consent to the language of their country. Translated forms will be centrally collected and available to local hospitals upon request. In this procedure, a SIOPE Network member informs parents (and patients), after which he/she provides the Patient Information Form (Supplementary material 2) and requests for informed consent. Parents or patients may reject participation at all times.

#### Data collection

Each country represented in the SIOPE DIPG Network is committed to delivering data to the SIOPE DIPG Registry and Imaging Repository. After subscription to the Network, the approved Network member receives a username and password to enter data into the Registry. Data collection covers both retrospective and prospective registration. Retrospective data will be collected from local hospitals, national registries and clinical trials. For prospective registration, Network members are encouraged to inform their patients about the existence of the SIOPE DIPG Registry followed by the informed consent procedure. In case of decline, the e-CRFs will be left blank, but a unique Registry number is created, which will only be used for epidemiologic studies. To describe data retrieval, as well as responsibility and ownership of the data, uniform international agreements for collaborative research purposes were created (see Legal aspects).

#### Exhaustivity check and quality control of the data

To ensure the reliability, validity, and completeness of the data [17], an appropriate program of Quality Control was implemented (Supplementary Fig. 1). Quality Control of data is an integral part of the project and takes place at all stages: before, during and after data entry.

#### Data storage and safety

Based on the e-CRF’s, an optimized relational database was constructed. The database along with the web application is hosted on a dedicated server where the web application is the single point of contact with the database. All end-user connections use the secure HTTP (HTTPS) protocol to ensure protection of the privacy and integrity of the exchanged data. The server is placed within a Virtual Private LAN protected by a dedicated firewall ring. For server maintenance purposes direct access to the server is only possible through a restricted virtual private network (VPN) connection. The DIPG Registry is built on a generic framework in which presentation, logic and data layer are separated. The framework was designed with several active protection features to prevent unsolicited use of the application such as user/role/session validation, the use of antiforgery tokens and brute force protection. To ensure data safety, database input controls and extensive audit trailing are used. Every action within the system and the database is logged. The server, application and database are monitored 24h/7days and backups are made and stored daily on a different server in order to provide a disaster recovery scenario. The SIOPE DIPG Registry framework herewith provides a stable, secure and generic basis in any of its products. A penetration test (black box approach) was performed to validate the effectiveness of the (visible) security implemented on the SIOPE DIPG Registry and Imaging Repository. This test will be repeated on a regular basis.

#### Legal aspects

The daily and financial management, and hosting of the SIOPE DIPG Registry is carried out by the Dutch Childhood Oncology Group (DCOG), a National Paediatric Haematology-Oncology Society (NaPHOS) member of SIOPE. DCOG is mandated by the Executive Committee of the DIPG Network to act as a legal entity on its behalf in matters concerning the DIPG Registry, by a letter of mandate.

The construction of a collaborative research infrastructure, with geographical differences in health care structures and legislation faces considerable challenges. Experts in the field of sensitive data transfer and access rights have been consulted to certify issues concerning data anonymization, -collection, and -safety. To meet multinational standards, two legal documents have been drafted, abiding to EU law and taking into account SIOPE DIPG Network members’ national laws. The first contains the SIOPE DIPG Network Bylaws (Supplementary material 3), that describe the mission, aims, core values and structure of the SIOPE DIPG Network as well as terms and conditions for submitting, reviewing and approving proposals for research projects using data from the SIOPE DIPG Registry. Furthermore, the Bylaws provide a Scientific Advisory Agreement for consultation of experts outside the SIOPE DIPG Network, such as specialised neuroradiologist for central radiology review. Second is the SIOPE DIPG Registry and Imaging Repository Regulatory Document (Supplementary material 4), describing the terms and conditions for management, maintenance of and access to the DIPG Registry and Imaging Repository.

#### Use of data

For strategic decisions concerning novel collaborative clinical and biological research projects in the field of DIPG, NCs meet or consult several times a year. In this way the SIOPE DIPG Network itself is responsible for the optimal use of obtained data. Data from the SIOPE DIPG Registry and Imaging Repository are available to researchers for collaborative, interdisciplinary, and translational studies. For use of the data from the Registry, the researcher must be a member of the SIOPE DIPG Network. The availability of data to the researcher is conditional to obtained approval from the Executive Committee, after submission of a project proposal, and permits and licenses required by the researcher’s national law. The Executive Committee may set additional conditions to a specific project and stipulates the general terms and conditions with regard to receipt and use of data. Subsequently, only requested, relevant data are selected from the DIPG Registry and made available to the researcher. The researcher owns results of a research project, including the intellectual property rights thereto. Publication of results generated with data from the SIOPE DIPG Registry requires to comply with rules concerning authorship, as defined by the International Committee of Medical Journal Editors (ICMJE). Each year, the Executive Committee sends a report to the members of the SIOPE DIPG Network on the number of approved, performed and rejected projects.

## Results

### International collaboration in DIPG research

Since its inception in 2011, the SIOPE DIPG Network has expanded each year. Currently, 27 countries (Austria, Belgium, Croatia, Czech Republic, Denmark, Finland, France, Germany, Greece, Hungary, Iceland, Ireland, Italy, Lithuania, Norway, Poland, Portugal, Slovakia, Slovenia, Spain, Sweden, Switzerland, The Netherlands, United Kingdom, Turkey, Russia, and Mexico; Supplementary Fig. 2) have committed to the SIOPE DIPG Network and Registry. There is also a close collaboration with the International DIPG Registry, which represents the collaborative efforts of physicians and researchers from North America, Canada, Australia and New Zealand (Supplementary Fig. 2). To coordinate similar data collection, there are frequent telephone conferences and annual working visits between the SIOPE DIPG Network chair, the SIOPE DIPG Registry coordinator and International DIPG Registry team stationed at the Clinical Management and Research Support Core (CMRSC) at Cincinnati Children’s Hospital Medical Center. Both DIPG registries are financially supported by The DIPG Collaborative, a collection of more than 20 parent foundations with the common interest of promoting and funding research into DIPG.

### SIOPE DIPG registry and imaging repository

**Fig. 3:**
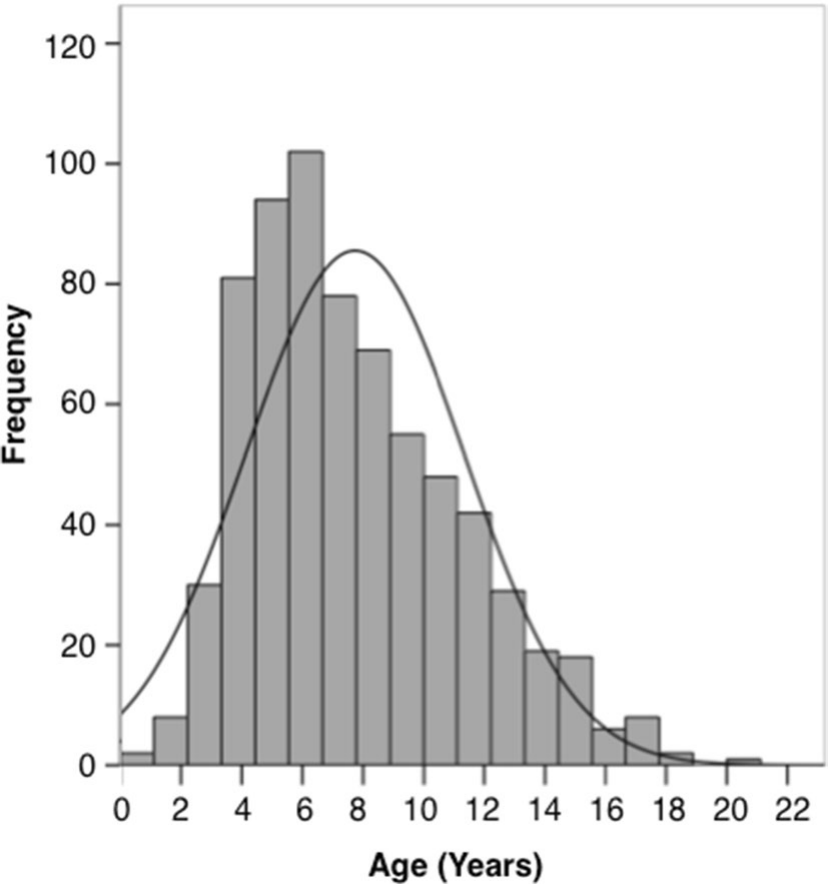
Histogram showing the age distribution of the total cohort

**Fig. 4:**
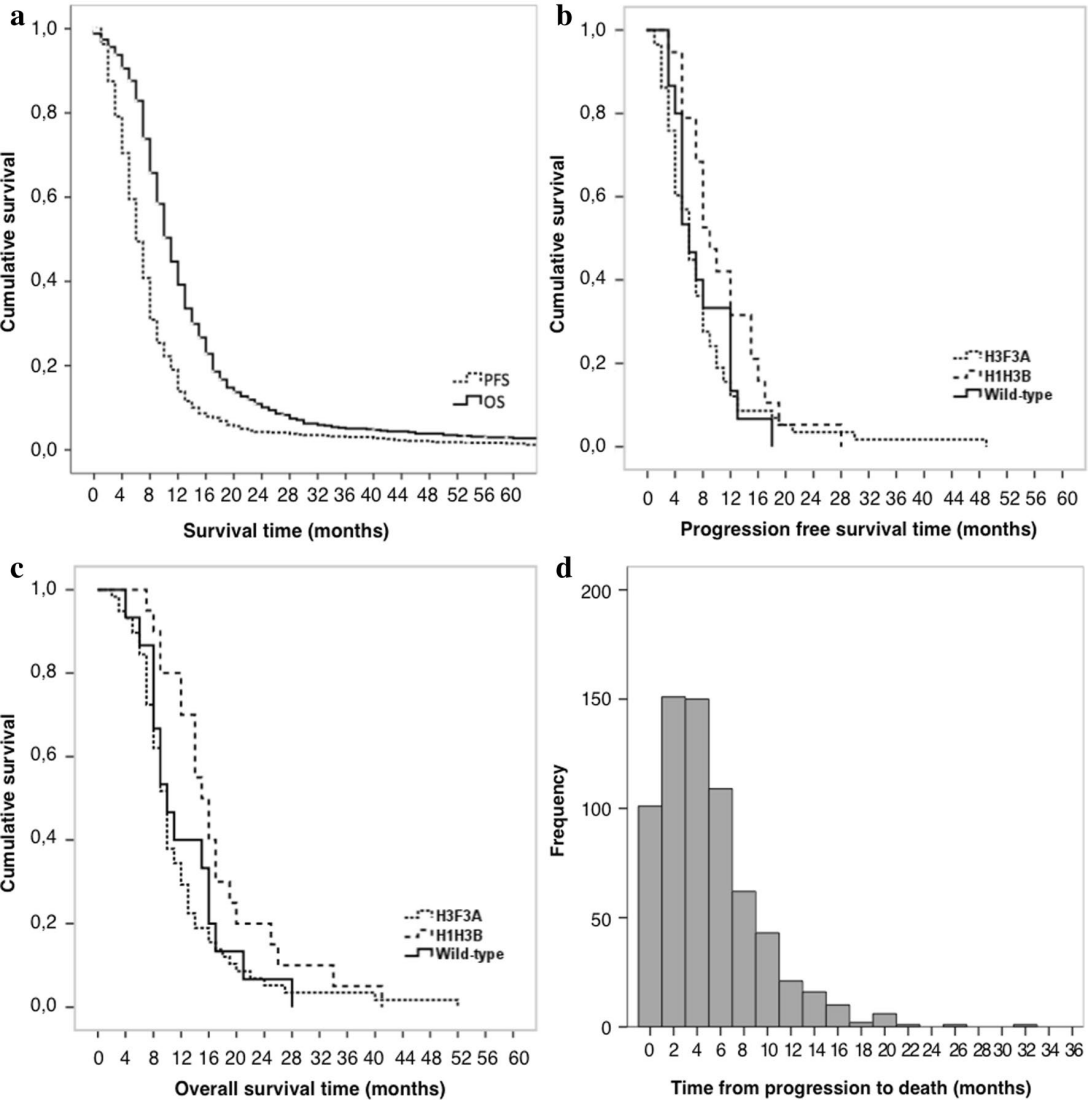
Survival data. **a** Kaplan Meier estimates of progression free survival (PFS; n = 684) and overall survival (OS; n = 691). **b** Kaplan Meier estimates of progression free survival (PFS) stratified by mutational status (H3F3A n = 59, H1H3B n = 20, wild-type n = 15). **c** Kaplan Meier estimates of overall survival (OS) stratified by mutational status (H3F3A n = 59, H1H3B n = 20, wild-type n = 15). **d** Histogram showing the distribution of time from progression to death

Currently, as a prerequisite to start prospective patient inclusion in the SIOPE DIPG Registry, members of the SIOPE DIPG Network are in the process of Medical Ethical Committee and IRB review, with some countries already including patient data upon approval. As of April 2016, six countries have submitted retrospective data of 694 patients to the SIOPE DIPG Registry and Imaging Repository. Data were retrieved from three national registries, two local hospitals, and one clinical trial. Figure 3 shows the age distribution of patients included in the SIOPE DIPG Registry, with a median age of 7 years (standard deviation (SD) ± 3.5). Table 1 shows the patient characteristics, clinical, radiological and biological disease characteristics, and treatment details of the total cohort. For 94 patients, tumor material was available for genetic analysis. Results are shown in Table 2. The median progression free survival, defined as time from diagnosis to clinical signs of disease progression (i.e., increase of symptoms or new symptoms) and/or radiological tumor progression on MRI, was 6.0 months (95% Confidence Interval (CI) 5.6–6.4 months). The median OS, defined as time from diagnosis to death, was 11.0 months (95% CI 10.5–11.5 months). PFS and OS are both plotted in Fig. 4a. Figure 4b, c show the PFS and OS stratified by mutational status. Figure 4d, finally, shows the distribution of time from progression to death (median 4 months). Ten percent of patients were alive at 2 years post diagnosis. At 5 years post diagnosis only two percent were alive. No disease-free survival was observed.

**Table 1:** Demographics, disease characteristics and treatment data of the total cohort (n = 694)

Category	Variable	n	Valid (%)
Total		694	
Country	Germany	312/694	45
	Netherlands	132/694	19
	France	118/694	17
	Italy	79/694	11
	United Kingdom	45/694	7
	Croatia	8/694	1
Gender	Female	359/694	52
	Male	335/694	48
Age	(mean, SD)	7.7	± 3.5
Symptom duration	<6 weeks	413/627	66
	6–12 weeks	127/627	20
	13–24 weeks	47/627	8
	>24 weeks	40/627	6
Cranial nerve palsy	Yes	484/568	85
	No	84/568	15
Pyramidal signs	Yes	270/562	48
	No	292/562	52
Cerebellar signs	Yes	338/562	60
	No	224/562	40
T1-weighted	Hypo-intense	422/439	96
	Iso-intense	16/439	4
	Hyper-intense	1/439	0
T2-weighted	Hypo-intense	5/465	1
	Iso-intense	2/465	0
	Hyper-intense	458/465	99
Pontine involvement	<50%	3/550	0
	>50%	547/550	100
Tumor size	Anterior-posterior Ø in mm (mean, SD)	36	±7
	Transverse Ø in mm (mean, SD)	43	±8
	Cranial-caudal Ø in mm (mean, SD)	42	±9
Enhancement	Yes	336/516	65
	No	180/516	35
Ring-enhancement	Yes	191/491	39
	No	300/491	61
Margin	Ill-defined	363/481	76
	Well-defined	118/481	24
Extension	Yes	493/549	90
	No	56/549	10
Metastasis brain	Yes	7/547	1
	No	540/547	99
Metastasis spine	Yes	8/420	2
	No	412/420	98
Hemorrhage	Yes	60/458	13
	No	398/458	87
Necrosis	Yes	191/473	40
	No	282/473	60
Hydrocephalus	Yes	89/505	18
	No	416/505	82
Radiation	Yes	650/691	94
	No	41/691	6
Chemotherapy at diagnosis	Yes	498/689	72
	*Oral	252/495	51
	*IV	230/495	46
	*Both	13/495	3
	*Cytotoxic	323/495	65
	*Targeted	129/495	26
	*Both	43/495	9
	*EGFR	111/495	22
	*mTOR / PI3K	15/495	3
	*EGFR/mTOR	1/495	0
	*HDAC inhibitor	37/495	8
	*Other	331/495	67
	No	191/689	28
Chemotherapy at progressive disease	Yes	370/684	54
	No	314/684	46
Re-irradiation	Yes	61/694	9
	No	633/694	91
Hydrocephalus treatment	Yes	158/694	23
	No	536/694	77
Biopsy	Yes	260/694	37
	*WHO Grade IV	91/260	35
	*Glioblastoma multiforme	76/91	84
	*DIPG^	15/91	16
	*WHO Grade III	71/260	27
	*Anaplastic astrocytoma	61/71	86
	*Anaplastic oligoastrocytoma	8/71	11
	*Anaplastic oligodendroglioma	2/71	3
	*WHO Grade II	38/260	15
	*Diffuse astrocytoma	20/38	53
	*Low-grade astrocytoma n.o.s	11/38	29
	*Fibrillary astrocytoma	4/38	10
	*Oligoastrocytoma	2/38	5
	*Oligodendroglioma	1/38	3
	*WHO Grade unknown	60/260	23
	No	434/694	63
Autopsy	Yes	16/380	4
	*WHO Grade IV	12/16	75
	*Glioblastoma multiforme	12/12	100
	*WHO Grade II-IV	1/16	6
	*Astrocytoma	1/1	100
	*WHO Grade unknown	3/16	19
	No	364/380	96

*SD* Standard Deviation,* AP* Anterior-posterior,* WHO* World Health Organization, ^*^Following the 2016 WHO classification criteria [15]

**Table 2:** Genetic characteristics of patients with available tumor material (n = 94)

Category	Variable	n	VALID %
Total		94	
Material type	Biopsy	86/94	92
	Autopsy	8/94	8
Histone mutations	H3F3A	59/94	63
	H1H3B	20/94	21
	H1H3C	0/16	–
	H1H3I	0/16	–
	Wild-type	15/94	16
Additional mutations	ACVR1	9/45	17
	Wild-type	45/54	83
	TP53	18/29	62
	Wild-type	11/29	38
	ATM	3/16	19
	Wild-type	13/16	81
	PIK3CA	5/30	17
	Wild-type	25/30	83
	PIK3R1	3/15	20
	Wild-type	12/15	80
	MET	1/15	7
	Wild-type	14/15	93

## Discussion

A first step is made to improve the infrastructure of research into DIPG. This was done by (1) the establishment of the SIOPE DIPG Network, and (2) the development and initiation of the SIOPE DIPG Registry and Imaging Repository. This initiative, enabling collaborative research, is seen as major first step towards improving care and (ultimately) cure for children with DIPG.

Collaboration is pursued to overcome the factors hampering research into DIPG. This paper is the first to publish pooled patient data of almost 700 DIPG patients collected from national registries, local hospitals and clinical trials. To date, published patient data are largely from phase I/II trials, which cover only a small percentage of the actual population diagnosed with DIPG. This possibly results in publication/selection bias. Future registration of *all* DIPG patients, both in- and outside trials, will give the opportunity to analyze ‘real-life’ DIPG patient data resulting in better description of incidence, characteristics and survival of DIPG patients. Also, it will generate a representative reference cohort, which may be used as historical control in any future study. With the SIOPE DIPG Network and SIOPE DIPG Registry/Imaging Repository, an infrastructure has been created that allows for research transparency, international collaboration and the elimination of duplication of research efforts. Already two international studies were published by the SIOPE DIPG Network, concerning palliative care and end-of-life decisions [18] and steroid use [19] in DIPG patients. The first large-scale international study including all patients registered in the SIOPE DIPG Registry and International Registry, with an estimated total of >1000 DIPG cases, is currently being conducted. This study will evaluate the characteristics of long-term surviving patients in comparison to the total group of patients.

The preliminary patient data of the 694 patients currently included in the SIOPE DIPG Registry, shows an equal gender distribution, rapid onset of symptoms pre-diagnosis (86% <12 weeks of which 66% within 6 weeks), a clinical presentation including cranial nerve palsy in the majority (85%) of patients, and two-third of patients showing gadolinium contrast enhancement on the diagnostic MRI, of which 57% (39% of the total cohort) showed partial ring-like enhancement suggestive for necrosis. At time of diagnosis, only 1% of the diagnostic MRIs showed metastasis in the brain, and 2% in the spine. Eighteen percent of patients present with hydrocephalus. Biopsy was performed in one-third of the patients, showing a range of WHO grades. From the 94 patients in whom histone mutational status was determined, two-third harbored a H3F3A mutation, versus 21% of patients harboring a H1H3B mutation, and 16 % were classified as wild-type. This distribution, as well as the observed difference in survival in favor of the H1H3B mutational subgroup, is in line with international literature [4, 6, 9, 20]. Almost all patients received radiotherapy, 9% received re-irradiation, and a sticking 72% received chemotherapy, which is contradictory since there is no chemotherapeutic strategy yet, that has shown to be effective [1, 2]. Autopsy was performed in only 4% of patients. Currently, the majority of patients included in the SIOPE DIPG Registry are patients with a radiologically confirmed and centrally reviewed T1-weighted hypointense and T2-weighted hyperintense tumor with at least 50% involvement of the pons (DIPG) [11]. The recent WHO re-classification, however, may imply that the inclusion criteria for the SIOPE DIPG Registry need to be adjusted to also include patients with non-pontine diffuse midline gliomas in the future.

Dependent on the extent to which biopsies and autopsies will be (re-)introduced for DIPG, data on biological characteristics will gradually increment in the Registry, which will increase the knowledge on DIPG etiology, pathogenesis, possible drug targets and the mechanisms that contribute to the observed resistance to treatment. Furthermore, big-data analysis of aggregated clinical, radiological and especially biological data facilitates the discovery of patterns that indicate patient subgroups, which enables consensus formation on classification, in-/exclusion and response criteria, and improves the quality and comparability of future trials. Moreover, joining forces within an international research-infrastructure will stimulate the initiation of, and active accrual in, international multicenter trials, with sufficient power to address the many unanswered research questions. This, together with the recent evolution of ideas concerning therapeutic strategies [21–24], should facilitate the identification and selection of novel tolerable and effective therapies.

Data collection in the Registry will have some (initial) limitations. Due to the former lack of local hospital- and national registrations, lack of specific ICD-codes^1^, and due to a presumed limited documentation of clinical, radiological and pathological data, retrospective data collection will very likely be incomplete. Based on data from the Dutch retrospective study [10], and included parties in the SIOPE DIPG Network (with a total number of about 600 million residents aged 0–19 years; April 2016) it is estimated that over 350 children are eligible for prospective registration in the SIOPE DIPG Registry each year. It is expected that annually about 200 patients (60%) will be registered in the first years, and that this number will increase when the SIOPE DIPG Network expands, resulting in higher data completeness per country over time.

Recent publications in DIPG literature have shown that coupling genetic data to clinical data will become increasingly important to understand and/or predict the clinical behavior of the disease [9, 14]. Therefore, as for now, data of the most common genetic aberrations are entered in the Registry via a ‘Biopsy/Autopsy e-CRF’. A next step of the SIOPE DIPG Registry is to establish a (virtual) biobank of DIPG material, linked with the DIPG Genomics Repository at Progenetix (dipg.progenetix.org), a cancer genome database [25]. Ideally, the increased availability of DIPG tumor tissue will lead to generally available, representative, and possibly even patient subgroup-specific cell cultures and xenograft models, which enable thorough basic research and high-throughput screening of candidate therapies. Other future perspectives are to include questionnaires for Quality of Life research since research on this important subject is largely lacking, especially data on end-stage disease symptoms and the associated specific needs for palliative and end-of-life care [18]. The collection of conventional MR-imaging data in the Imaging Repository, will in the future be expanded to multimodality MR-imaging and other advanced imaging techniques such as PET. The data also might be useful for educational purposes (e-learning) in an aim to improve diagnostics of these tumors.

To conclude, with the collaborative efforts of professionals treating children with DIPG, patient/parent organizations, legal advisors, experts in the field of information technology and imaging experts, an international research-infrastructure was successfully set up, which led to the development and initiation of the SIOPE DIPG Registry. With already 694 patients registered, this Registry stimulates collaborative preclinical and clinical research efforts. The first study using data from both the SIOPE and International Registry is already in its final stages. The existence of the International DIPG Registry, surveying similar data as the SIOPE DIPG Registry, allows for external cross-validation of data, generating robust data on the DIPG patient population. Big data analysis of the Registry’s data will potentially lead to the discovery of patterns that pave the way to the identification of effective therapies towards a cure for patients suffering from DIPG.

The methodology used for the SIOPE DIPG Registry will, most likely, be easily translatable to other pediatric cancer registries, as almost all of these are orphan diseases that could benefit from international registration and collaboration in research.

## Electronic supplementary material

Below is the link to the electronic supplementary material.

Supplementary material 1 (DOCX 547 KB)

Supplementary material 2 (DOCX 95 KB)

Supplementary material 3 (DOCX 154 KB)

Supplementary material 4 (DOC 67 KB)

## Footnotes

1 World Health Organisation (WHO) International Classification of Diseases (ICD).
