# Supplementary material for: Development of the SIOPE DIPG network, registry and imaging repository: a collaborative effort to optimize research into a rare and lethal disease
Source: J Neurooncol. 2017 Jan 21;132(2):255–66. doi: 10.1007/s11060-016-2363-y (PMC5378734; doi:10.1007/s11060-016-2363-y)
Supplement: Supplementary file 1 — Supplementary material 1 (DOCX 547 KB) [file 11060_2016_2363_MOESM1_ESM.docx]

**Development of the SIOPE DIPG Network, Registry and Imaging Repository:**

**A collaborative effort to optimize research into a rare and lethal disease.**

Journal of Neuro-Oncology

*Sophie E.M. Veldhuijzen van Zanten, Joshua Baugh and Brooklyn Chaney, Dennis De Jongh, Esther Sanchez Aliaga, Frederik Barkhof, Johan Noltes, Ruben De Wolf, Jet Van Dijk, Antonio Cannarozzo, Carin M. Damen-Korbijn, Jan A. Lieverst, Niclas Colditz, Marion Hoffmann, Monika Warmuth-Metz, Brigitte Bison, David T.W. Jones, Dominik Sturm, Gerrit H. Gielen, Chris Jones, Esther Hulleman, Raphael Calmon, David Castel, Pascale Varlet, Géraldine Giraud, Irene Slavc, Stefaan Van Gool, Sandra Jacobs, Filip Jadrijevic-Cvrlje, David Sumerauer, Karsten Nysom, Virve Pentikainen, Sanna-Maria Kivivuori, Pierre Leblond, Natasha Entz-Werle, Andre O. von Bueren, Antonis Kattamis, Darren Hargrave, Péter Hauser, Miklos Garami, Halldora Kristin Thorarinsdottir, Jane Pears, Lorenza Gandola, Giedre Rutkauskiene, Geert O. Janssens, Ingrid K. Torsvik, Marta Perek-Polnik, Maria João Gil-da-Costa, Olga Zheludkova, Liudmila Shats, Ladislav Deak, Lidija Kitanovski, Ofelia Cruz, Andres Morales La Madrid, Stefan Holm, Nicolas Gerber, Rejin Kebudi, Richard Grundy, Enrique Lopez-Aguilar, Marta Zapata-Tarres, John Emmerik, Tim Hayden, Simon Bailey, Veronica Biassoni, Maura Massimino, Jacques Grill, William P. Vandertop, Gertjan J.L. Kaspers, Maryam Fouladi, Christof M. Kramm, Dannis G. van Vuurden on behalf of the members of the SIOPE DIPG Network.*

**Corresponsing author: Sophie E.M. Veldhuijzen van Zanten, VU University Medical Center Department of Pediatrics, Division of Oncology-Hematology, s.veldhuijzen@vumc.nl**

**
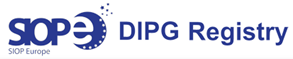

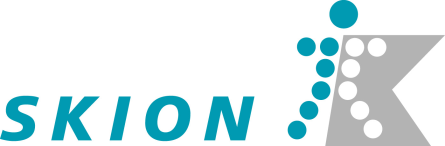
**


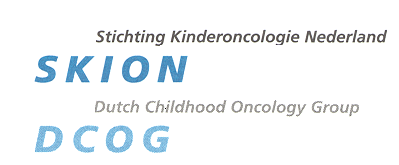


**Data Entry Manual**

| Protocol version and date: | V 1.1, 1-June-2016 |
| --- | --- |
| Version of this manual: | V 1.0, 1-June-2016 |

# TABLE OF CONTENTS

TABLE OF CONTENTS 2

1. Introduction 4

1.1 Background 4

1.2 The SIOPE DIPG Registry and Imaging Repository 4

1.3 Registry perspectives 4

1.4 Registry coordination 5

2. Registry account 6

2.1 Account issuing 6

2.2 Sign in 6

2.3 Downloads after signing in 8

2.4 Password forgotten 8

2.5 Update account details 9

3. Data collection 10

3.1 Population 10

3.2 What data will be collected? 10

3.3 Common conventions 10

4. Get started: add, search and edit a patient 12

4.1 Add a patient 12

4.2 Search and edit Patient data 13

4.3 View CRF 14

4.4 Back 14

4.5 Lock Patient File 14

4.6 Validate File 14

4.7 Edit View Rights 15

4.8 Inactivity logout time 15

5. CRF 1 - REGISTRATION 16

6. CRF 2 - Data concerning “History and Physical exam” 18

6.1 History 18

6.2 Physical exam 20

7. CRF 3 - Data concerning “Diagnosis and Imaging” 25

7.1 MRI brain 25

7.2 MRI spine 26

7.3 MR Spectroscopy 26

7.4 PET 27

7.5 CT 27

7.6 Biopsy 27

7.7 CSF cytology 29

8. CRF 4 - Data concerning “Treatment” 31

8.1 Radiotherapy 32

8.2 Chemotherapy 33

8.3 Surgery 36

8.4 Steroids 37

9. CRF 5 - Data concerning “Response evaluation” 39

9.1 Clinical response evaluation 39

9.2 MRI/CT 41

10. CRF 6 - Data concerning “Follow up” 44

# Introduction

## Background

The SIOPE DIPG registry was build based on international agreements, with the aim to collect uniform data concerning baseline characteristics, clinical presentation, radiological characteristics, tumour biology, quality of life, and response to treatment. The SIOPE DIPG Registry will therewith create the basis for joint European trials and could be of great importance in improving the survival of children suffering DIPG.

## The SIOPE DIPG Registry and Imaging Repository

The SIOPE DIPG registry exists of two entities:

- An online Case Report Form (eCRF)-structured web application and database for clinical data
  ([www.dipgregistry.eu](http://www.dipgregistry.eu));
- An imaging repository system for radiological images;

This manual is solely intended to assist data entry into the Registry specific eCRFs.

The CRFs have been developed by the SIOPE DIPG Network, in close collaboration with colleagues from the USA and Canada.

The clinical database is hosted by the Dutch Childhood Oncology group (DCOG/SKION), which is located in The Hague (Netherlands). The DCOG will provide quality control and storage of incoming data.

## Registry perspectives

The SIOPE DIPG Registry has two perspectives:

1. **Bringing together a retrospective cohort.**From this perspective, the aim is to include all patients diagnosed between 1990 and 2014. This will provide data of (mostly deceased) patients, which are added to the Registry anonymously. For this, no informed consent is mandatory.
2. **A prospective registration.**

Once in a Local Research Site all Legal and Ethical Agreements are approved Local Coordinators are encouraged to prospectively include all patients diagnosed with DIPG after informing parents (and patients), providing the Patient Information Form and requesting for Informed Consent.

Physicians treating children with DIPG are asked to inform their patients about the SIOPE DIPG Registry. Patients may reject participation at all times. In that case we do ask Local Coordinators to report the patients and generate a unique Registry number via the “Add Patient” form (see Paragraph 4.1). The CRFs will in that case be left blank. The Registry number and Registration Form will be used for epidemiologic studies only.

## Registry coordination

The Registry Coordinator will coordinate daily business. The Registry coordinator is responsible for:

- the distribution of (legal) documents,
- the provision of accounts/passwords for the Registry,
- supporting logistics of incoming data (quality control and storage, link of clinical data in Registry and Imaging Repository),
- the contact with National Coordinators and other Registry data providers concerning:
  - erroneous or missing values in the incoming data
  - support in case of a technical system failure
  - FAQs
- coordination of European neuro-radiologists’ central review of images from the Imaging Repository,
- keeping track of participants (e.g. institutions, national groups, etc.),
- keeping track of DIPG Registry accrual by providing a 6-monthly update to all members of the SIOPE DIPG Network,
- keeping an ‘up to date’ list of items to be ascertained (CRF) in the DIPG Registry and Imaging Repository, based on demands from the field / DIPG Network.

In case of questions, please contact the Registry Coordinator : [ckorbijn@skion.nl](mailto:ckorbijn@skion.nl)

# Registry account

## Account issuing

Potential member sites of the DIPG Network will be selected by the DIPG Network National Coordinators. A physician (requestor) from a participating site sends a request to join the DIPG Network and the DIPG Registry to the DIPG Network Executive Committee. After approval by the Executive Committee the chairman will send details to DCOG. Upon receipt of a signed ’Funding and Data Processing Agreement’ and a copy of an IRB/IEC approval letter, DCOG will create a new Registry account and the requestor will receive an automated e-mail including username and temporary password. This information will be sent from a no-reply address: [no_reply@2tci.nl](mailto:no_reply@2tci.nl). When the requestor sings in for the first time, the password needs to be changed.

**NB: this e-mail might be delivered to the junk e-mail folder.**

## Sign in

Open the internet browser and go to <https://www.dipgregistry.eu>.

The following screen will appear:


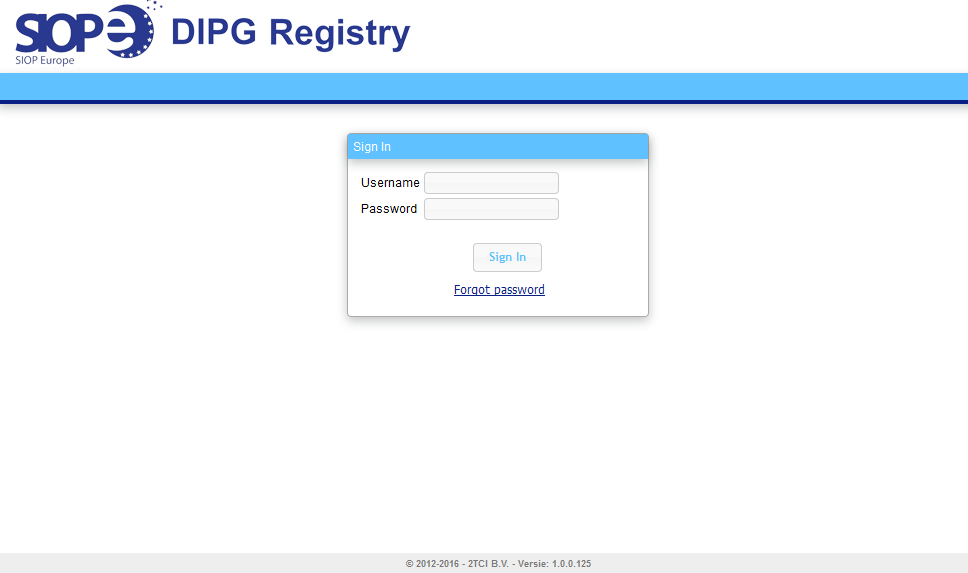


You will need to sign in using the username and password sent to you by automated e-mail. The following screen will appear and at first login you will have to change your temporary password:


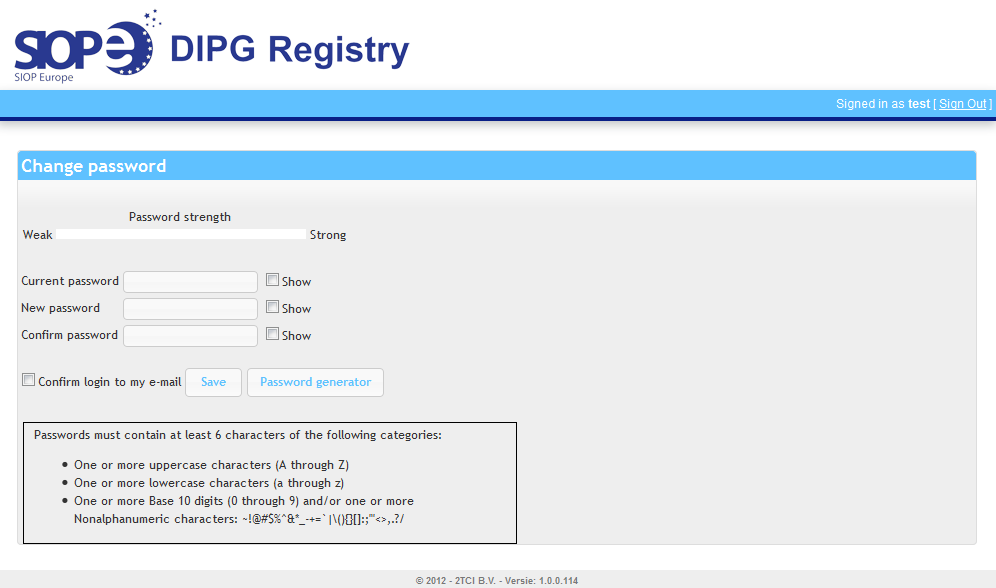


You can either choose your own password, provided it meets the complexity requirements, or let the system generate a password for you.
If you tick the box for confirmation of login, you will receive an automated e-mail that includes your username and new password.

Save your new password.

After saving your password, the following screen will automatically appear:


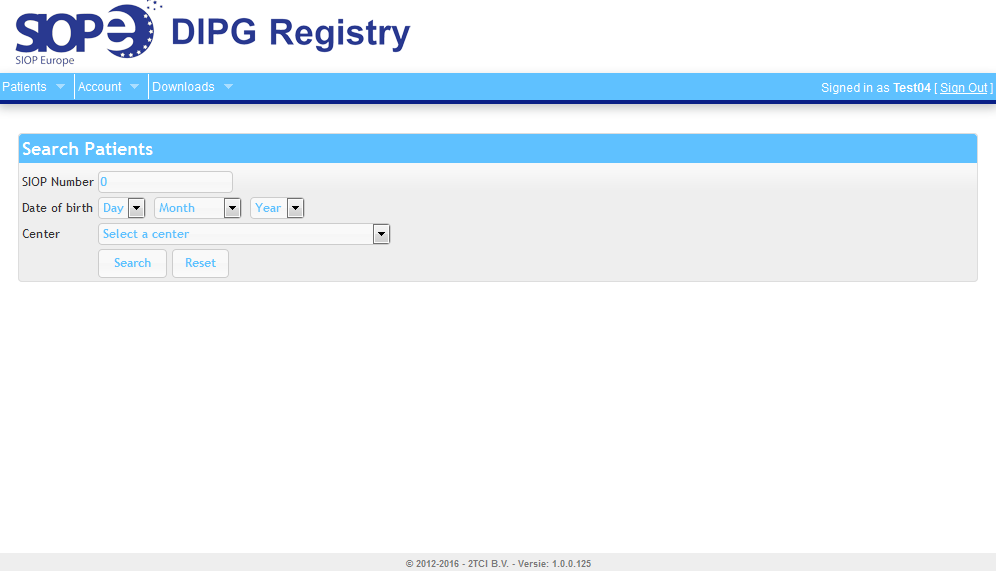


## Downloads after signing in

Once you’re signed in you can download SIOPE DIPG Registry related documents, such as the protocol, the Regulatory document and the Bylaws.


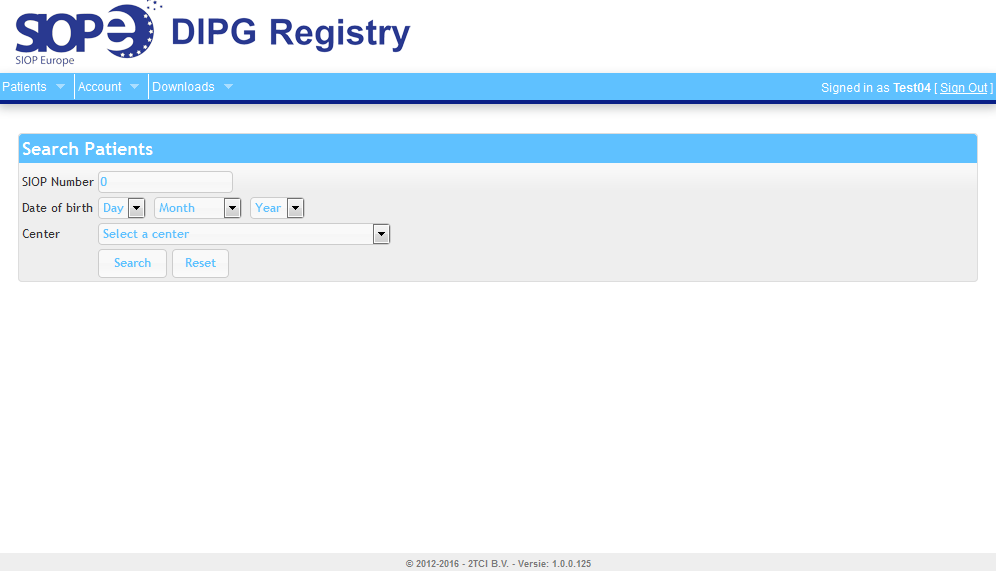


## Password forgotten

In case you have forgotten your password press the ‘Forgot password’ button and follow instructions.


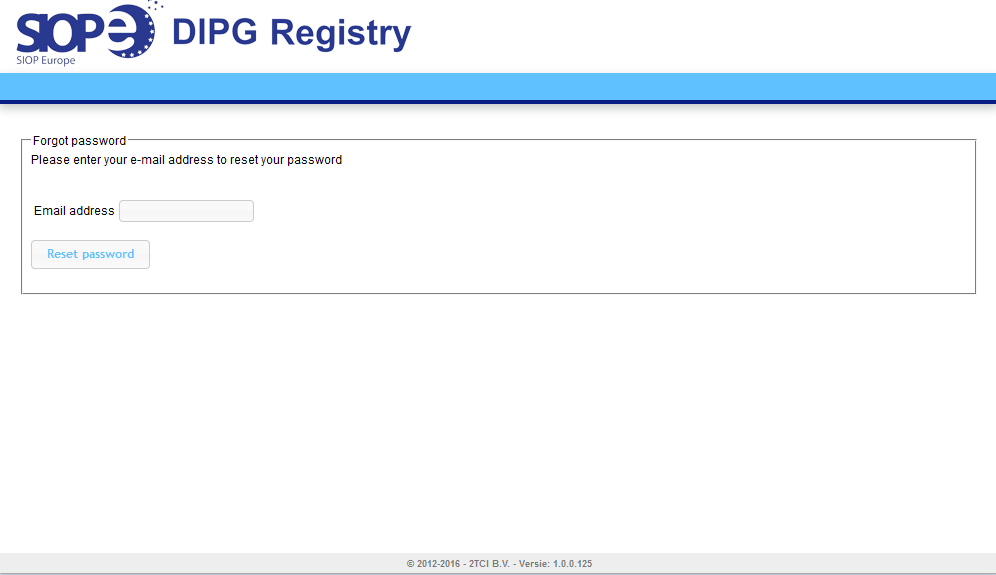


You will receive an automated e-mail that includes your username and new password.

## Update account details

Once you’re signed in, you can update your account (including password) details yourself.


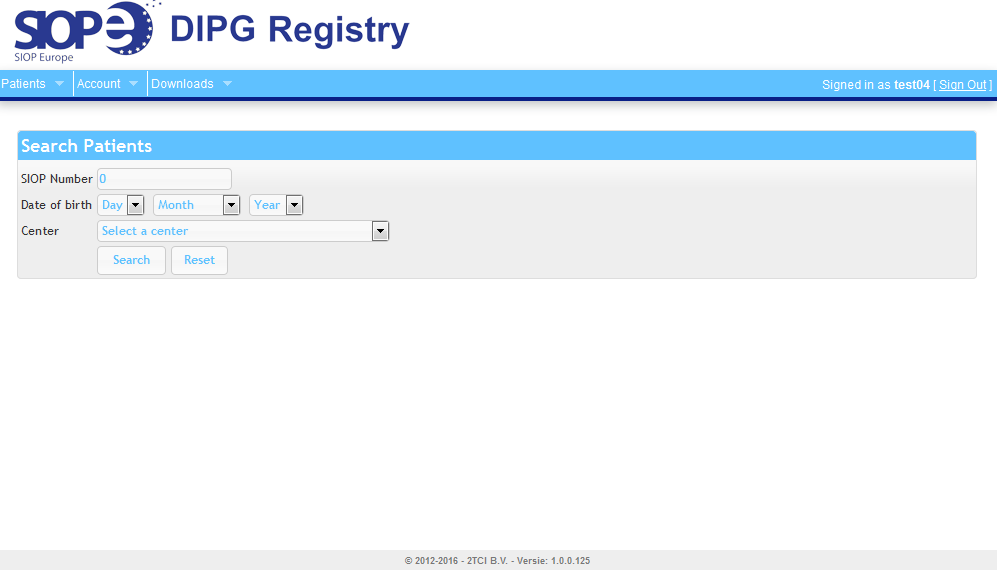


# Data collection

## Population

All paediatric patients with focal and diffuse intrinsic pontine gliomas are eligible for the European DIPG Registry. In order to be able to classify patients, a minimum of diagnostic criteria is required; i.e. clinical and radiological data (MRI images) and, if available, pathology data.

## What data will be collected?

Patients will prospectively be asked informed consent for data collection.

Complete data collection will include: demographics ( country, referring center, age, gender, date of initial diagnosis, time from first presenting symptoms, relevant past medical history & family history, etc.), symptoms & signs at diagnosis and their duration, MRI features at diagnosis and during disease (including, if available, advanced imaging techniques), treatment regimen, and clinical and radiological response to treatment.

In case participation is rejected the patient will only be registered through the electronic “Add a patient“ Form (see Paragraph 4.1). A unique Registry number will be obtained. However, additional CRFs will be left blank. The Registry number and data on the Registration Form will be used for epidemiologic studies only.

## Common conventions

- All items are included in web-based CRFs, in which the mandatory fields are indicated with the following sign:
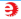
 or after saving a page will get a pop-up message :
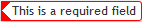

- Every CRF has to be saved separately.
- All dates are to be expressed in the standardized day-month-year format (dd-Month-yyyy, e.g., 1-January-2015). If you are not allowed to provide the full date due to privacy issues, please only provide the true month and year or the true year.
- When completing a free text field please enter **English** text and avoid the use of abbreviations for clarity.
- A field managed with a positive response, frequently indicated with a “Yes” or other specific data, must be supported by the corresponding source documentation. Any positive response must be confirmed by patient’s medical record.
- An indication of “No” may be used if the patient’s medical record confirms the field in question should be managed as such and is supported by the corresponding source documentation.
- An indication of “Unknown” may be acceptable if data are unavailable or missing from a patient’s medical record and no accurate and reliable assessment can be made.
- An indication of “Not applicable”(N/A) may be acceptable for any field in which the content does not apply.

If a complete patient’s medical record makes no mention of a field in question an indication of “N/A” is appropriate as it is assumed to have not been present if not reported.

- An indication of “Unspecified” may be acceptable if the field in question is confirmed positive; however, sufficient information is not available to confirm a sub-question (e.g. paresis is confirmed by the patient record; however, the side of the body impacted is not available).

# Get started: add, search and edit a patient

## Add a patient

To add a patient in the SIOPE DIPG Registry, go to tab ‘Patients’ in the top left of the webpage and click on ‘Add Patient’ and add (patient) details.


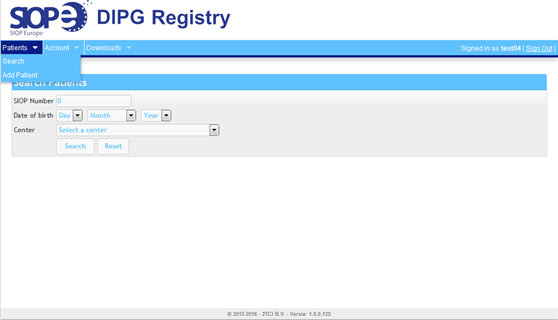


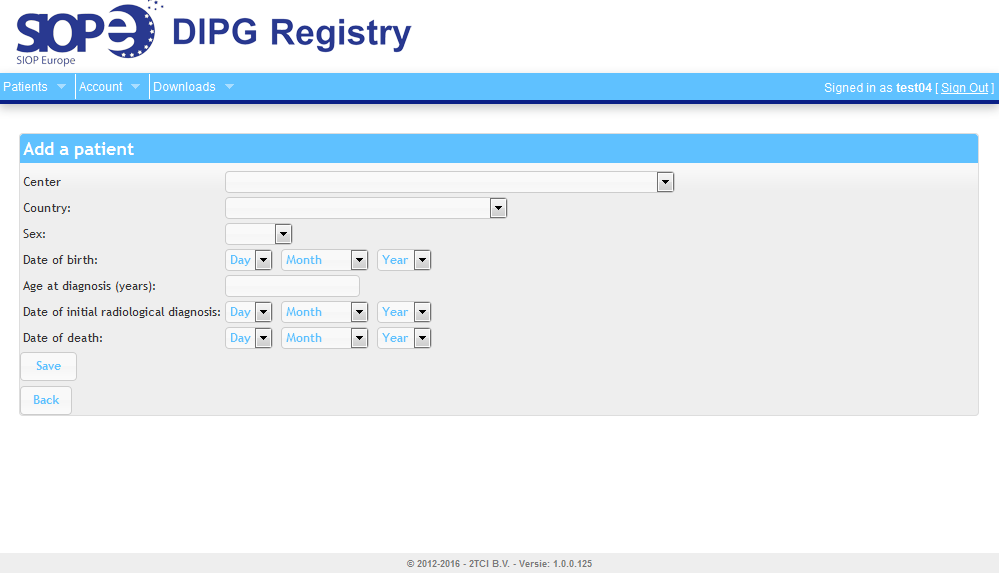


#### Center

#### Institution where the patient received the majority of his/her treatment.

#### Country

Country in which institution is located.

#### Sex

Patients’ sex/gender may be found in patient’s source documents, e.g. patient’s medical record.

#### Date of birth / Age at diagnosis(years)

Date of birth or Age at diagnosis may be found in patient’s source documents, e.g. patient’s medical record. If local privacy agreements do not allow entry of the full date of birth, you must answer ‘Age at diagnosis’. It is mandatory to give either full ‘Date of birth’ or ‘Age at diagnosis’.

Age should be rounded down (for example: ‘7’ for a child that is 7 years and 8 months old).
**Date of initial radiologic diagnosis**

Date of first MRI which provides support for diagnosis.

#### Date of death

Date of death may be found in death note, autopsy record, or other source documents.

**NB.** After saving the data by clicking the ‘Save’-button the **SIOP Number** will appear in a blue bar on the screen. Make a note of this SIOP Number and file it in an appropriate location.

## Search and edit Patient data

Once you have added one or more patients to the DIPG Registry, you will be able to edit and add data in a later stage. After signing in the following screen will appear automatically.


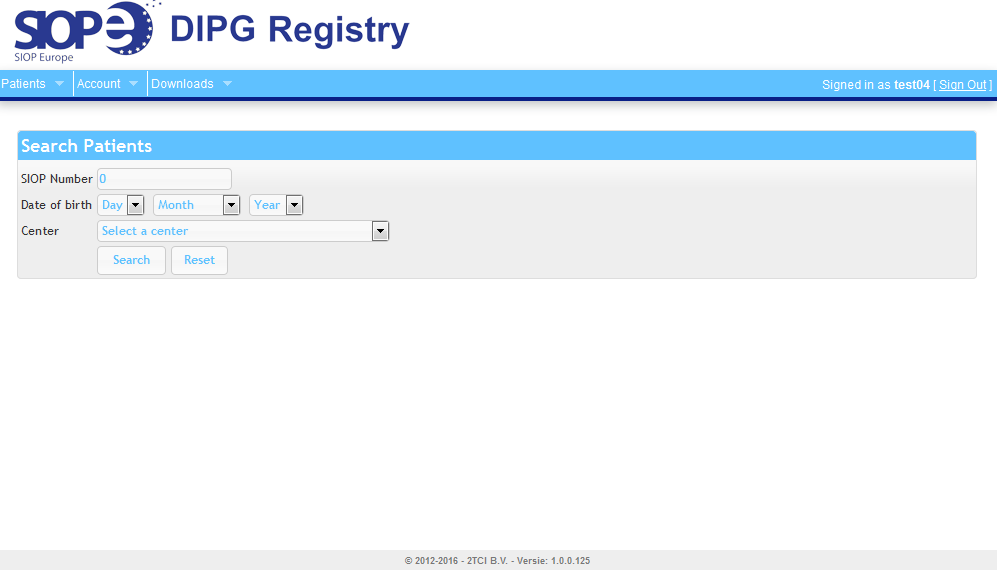


Now, you can search for a specific SIOP Number, date of birth (if this was entered before) or for all the patients from your site that have been added before. Click on the search button and a list of your selected patient(s) will appear.

You can sort this list by SIOP Number or Date of birth by clicking on the column header

Each record in this list will start with three icons:

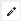
 for direct editing of the Patient data.

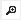
 for viewing the Patient data, and from there start editing if needed.

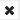
 for removing the patient from the SIOPE DIPG Registry database

Once you are in the Edit mode of the Patient data screen you will see 4 buttons :
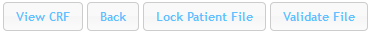


## View CRF

By clicking the ‘View CRF’-button you will be directed to the full electronic CRF with a tab for each subject, e.g. Registration, History & Physical Examination, Diagnosis & Imaging, Treatment, Response Evaluation, Follow Up.

## Back

By clicking the ‘Back’-button you will be re-directed to the ‘Search Patients’-screen.

## Lock Patient File

By clicking the ‘Lock patient File’-button the system will lock the patient file. You will not be able to edit any data for the patient until you click on the ‘Unlock Patient File’-button that appears after locking.
A Patient File should be locked only if data are complete and no further edits are to be expected.

## Validate File

By clicking the ‘Validate File’-button the system will check for missing mandatory data.

## Edit View Rights

In the upper right corner of the CRF screen a button to ‘Edit View Rights’ was added. By clicking the ‘Edit View Rights’ button one will be able to allow another site to also view the patient’s CRF.

## Inactivity logout time

After 20 minutes of inactivity users will automatically be logged out. Make sure you save your entries in time.

# CRF 1 - REGISTRATION

Data concerning demographics will be collected in CRF 1 - the Registration form.
Values entered while adding a patient to the DIPG Registry database (see Figure 4) will automatically be copied to the corresponding item fields in CRF 1 (Referring center, Country, Date of birth or Age at diagnosis, Sex, Date of initial radiological diagnosis)

Four additional questions have been included in this CRF:

- patient’s postal code

- time from first presenting symptoms to radiological diagnosis

- exact number of weeks between first symptoms and date of radiological diagnosis

- (calculated) age at time of diagnosis

#### Referring Center

Institution where the patient received the majority of his/her treatment.

#### Country

Country in which institution is located.

#### Patient’s postal code

Home address zip code may be found in patient’s medical record.

This field is not mandatory; if local privacy agreements do not allow inclusion of this information, please skip this question.

#### Date of birth

Date of birth may be found in patient’s source documents, e.g. patient’s medical record.

#### If local privacy agreements did not allow you to include full date of birth in the ‘Add a patient’-screen, no date will be shown here. Age at diagnosis (years)

If local privacy agreements only allow to include age at diagnosis instead of full date of birth, value for age from the ‘Add a Patient’ form will be shown here.

#### Sex

Patient sex/gender may be found in patient’s source documents, e.g. patient’s medical record.

#### Date of initial radiological diagnosis

Date of first MRI which provides support for diagnosis.

#### Time from first presenting symptoms

This refers to the onset of presenting symptoms, which are retrospectively attributed to DIPG. There is a choice of four subclasses: < 6 weeks, 6-12 weeks, >12 <= 24 weeks, >24 weeks. This information may be found patient’s medical record. Indicate ‘Unknown’ if information is not available.

#### If available, specify exact number of weeks from first presenting symptoms to diagnosis

This refers to the onset of presenting symptoms, which are retrospectively attributed to DIPG. Give round numbers. It is not a mandatory field.

#### (Calculated) Age at time of diagnosis (months)

This will be calculated automatically in case full date of birth and full date of diagnosis are given

**NB: Save the CRF after completing!**

# CRF 2 - Data concerning “History and Physical exam”

## History

Relevant past medical history and family history, and symptoms and signs at diagnosis will be collected. Therefore, a list of most likely signs and symptoms is created based on a comprehensive literature review. Please score the items documented in the initial history and physical examination retrospectively or prospectively during the first intake.

#### Date of history

Date of patients initial reported history. If date initial history is unavailable, use date of history nearest to MRI diagnosis.

#### Signs/symptoms

Signs and symptoms are frequently reported by patient’s parents or guardians.

Provide an answer to ***each*** sign or symptom.

Indicate ‘Yes’ only if a sign or symptom is confirmed in the patient’s medical record. Indicate ‘No’ if the record confirms the sign or symptom was not present or if no mention of the sign or symptom is present within a complete record. If a significant number of reports are missing from patient’s medical record so that patient history may not be assessed select ‘Unknown’.

#### Specify Other signs/symptoms

Include any other relevant physical, mental, and/or behavioural descriptions mentioned in the patient initial history. Please write in English and avoid the use of abbreviations for clarity.

#### Patient one of twins or triplets

Indicate ‘Yes’ only if the patient’s medical record confirms the patient was part of a multiple birth. Indicate ‘No’ if the record confirms patient was not a multiple birth or if no mention of multiple birth is present within a complete medical record. If a significant number of reports are missing from patient’s medical record so that patient history may not be assessed, select ‘Unknown’.

If patient was part of multiple birth, specify any additional relevant information mentioned in patient’s medical record.

#### Pre-existent genetic condition(s)

Pre-existent refers to any time prior to the onset of signs/symptoms. Indicate ‘Yes’ only if a pre-existent genetic condition is confirmed in the patient’s medical record. Indicate ‘No’ if the record confirms there are no pre-existent genetic conditions or if no mention of pre-existent genetic conditions is present within a complete medical record. If a significant number of reports are missing from patient’s medical record so that patient history may not be assessed, select ‘Unknown’.

If a genetic condition is confirmed, specify condition and any additional relevant information.

#### Pre-existent behavioural/psychological condition(s)

Pre-existent refers to any time prior to the onset of symptoms. Indicate ‘Yes’ only if a pre-existent behavioural/psychological condition is confirmed in the patient’s medical record. Indicate ‘No’ if the record confirms there are no pre-existent behavioural/psychological conditions or if no mention of pre-existent behavioural/psychological conditions is present within a complete medical record. If a significant number of reports are missing from patient’s medical record so that patient history may not be assessed, select ‘Unknown’.

If a pre-existent behavioural/psychological condition(s) or mental health concerns are stated in the patient’s medical record, specify condition and any additional relevant information.

#### Other pre-existent condition(s)

Include any additional conditions mentioned in the patient’s medical history not addressed by previous sections at any time prior to the onset of signs/symptoms. Indicate ‘Yes’ only if another pre-existent condition is confirmed in the patient’s medical record. Indicate ‘No’ if the record confirms there are no other pre-existent conditions or if no mention of other pre-existent conditions is present within a complete medical record. If a significant number of reports are missing from patient’s medical record so that patient history may not be assessed, select ‘Unknown’.
If there is an additional pre-existent condition, specify condition and any additional relevant information.

**Genetic condition(s)**

Indicate ‘Yes’ only if confirmed in the patient’s medical record. Indicate ‘No’ if the record confirms there are no pre-existent genetic conditions within the family or if no mention of pre-existent genetic conditions is present within a complete medical record. If a significant number of reports are missing from patient’s medical record so that patient/family history may not be assessed, select ‘Unknown’.

If applicable/available, specify all genetic conditions by individual family members.
**Cancer**
Indicate ‘Yes’ only if directly stated in the patient’s medical record. Indicate ‘No’ if the record states there is no cancer history within the family or if no mention of cancer history is present within a complete medical record. If a significant number of reports are missing from patient’s medical record so that patient/family history may not be assessed, select ‘Unknown’.

If applicable/available, specify all cancer by individual family members by type and age of onset.

**Consanguinity**

Indicate ’Yes’ only if inbreeding within the family is confirmed in the patient record. Indicate ’No’ if the patient record states there is no history of inbreeding within the family or if no mention of inbreeding is present within a complete record. If a significant number of reports are missing from a record so that patient history may not be assessed select ’Unknown’.

If applicable, specify individuals and relationship to the patient.

**Other pre-existent condition(s)**

Include any additional conditions mentioned in the family’s medical history not addressed by previous sections at any time prior to the onset of signs/symptoms. Indicate ‘Yes’ only if confirmed in the patient’s medical record. Indicate ‘No’ if the record confirms there are no other pre-existent conditions within the family or if no mention of other pre-existent conditions is present within a complete medical record. If a significant number of reports are missing from patient’s medical record

so that patient history may not be assessed, select ‘Unknown’.

If there is an additional condition within the family please specify condition and any additional relevant information.

## Physical exam

#### Date of physical exam

Date may be assessed from the initial neurologic exam performed at diagnosis. If initial exam is unavailable, use date of physical exam performed nearest to MRI diagnosis.

**Weight (kg)**

Patient weight at diagnosis should be assessed in patient’s medical record. Patient weight should be rounded to the nearest whole number if necessary.

If weight at time of diagnosis is not available, tick ‘Unknown’.

**Height (cm)**

Patient height at diagnosis should be assessed in patient’s medical record. Patient height should be rounded to the nearest whole number if necessary.

If height at time of diagnosis is not available, tick ‘Unknown’.

**Signs**Provide an answer to each sign.

*For Ataxia, Quadriplegia, Dysmetria, Diplopia and Other findings:*

Indicate ‘Yes’ only if a sign is confirmed in the patient’s medical record. Indicate ‘No’ if the record confirms the sign was not present or if no mention of the sign or symptom is present within a complete record. If a significant number of reports are missing from patient’s medical record so that patient history may not be assessed select ‘Unknown’.

*For Paresis, Hyperreflexia, Sensory loss, Babinski reflex, Papilledema, Cranial nerve palsy (II-XII):*

Indicate ‘Left ’, ‘Right’, or ‘Bilateral’ if patient was positively observed for this sign and side of the body impacted is clearly stated in patient’s medical record. If side of the body impacted cannot be assessed an indication of ‘Unspecified’ is acceptable. If the patient’s medical record confirms the sign was not present or if a complete patient record makes no mention of a sign an indication of ‘No’ is appropriate as it is assumed to have not been present if not reported. If an assessment was not completed or not done an indication of ‘Not tested’ is appropriate.

*For Monoplegia:*

Indicate ‘Left arm’, ‘Right arm’, ‘Left leg’ or ‘Right leg’ if patient was positively observed for this sign and side of the body impacted is clearly stated in patient’s medical record. If side of the body impacted cannot be assessed an indication of ‘Unspecified’ is acceptable. If the patient’s medical record confirms the sign was not present or if a complete patient record makes no mention of a sign an indication of ‘No’ is appropriate as it is assumed to have not been present if not reported. If an assessment was not completed or not done an indication of ‘Not tested’ is appropriate.

*For Hemiplegia:*

Indicate ‘Left’ or ‘Right’ if patient was positively observed for this sign and side of the body impacted is clearly stated in patient’s medical record. If side of the body impacted cannot be assessed an indication of ‘Unspecified’ is acceptable. If the patient’s medical record confirms the sign was not present or if a complete patient record makes no mention of the sign an indication of ‘No’ is appropriate as it is assumed to have not been present if not reported. If an assessment was not completed or not done an indication of ‘Not tested’ is appropriate.

**Ataxia:**

Ataxia describes a lack of muscle coordination during voluntary movements, for example difficulty walking or picking up objects. A sign of an underlying condition, ataxia can affect your movements, your speech, your eye movements and your ability to swallow.

#### Paresis:

Paresis is a condition typified by a weakness of voluntary movement, or partial loss of voluntary movement or by impaired movement. When used without qualifiers, it usually refers to the limbs, but it can also be used to describe the muscles of the eyes, the stomach, and also the vocal cords.

#### Hyperreflexia:

Hyperreflexia is defined as overactive or over-responsive reflexes. Examples of this can include twitching or spastic tendencies.

#### Sensory loss:

#### Sensory loss is defined as loss of function of one or more of the senses. Babinski reflex:

A positive sign is noted when the big toe moves upward toward the surface of the foot and the other toes fan out after the sole of the foot has been firmly stroked. In normal adults the plantar reflex causes a downward response.

#### Papilledema:

Papilledema is categorized as swelling of the head of the optic nerve and may be a sign of increased intracranial pressure.

#### Hemiplegia:

Hemiplegia is paralysis of the arm, leg, and trunk on the same side of the body.

#### Monoplegia:

Monoplegia is a paralysis of a single limb, usually an arm.

#### Quadriplegia:

Quadriplegia is paralysis that results in the partial or total loss of use of all limbs and torso.

#### Dysmetria:

Dysmetria refers to a lack of coordination of movement typified by the undershoot or overshoot of intended position with the hand, arm, leg, or eye. It is sometimes described as an inability to judge distance or scale.

#### Diplopia:

Diplopia is commonly known as double vision.

#### Cranial nerve palsy II:

The second cranial nerve is called the optic nerve. Palsy of the optic nerve may affect visual acuity, visual fields and/or the direct pupillary response to light.

#### Cranial nerve palsy III:

The third cranial nerve is called the oculomotor nerve. Palsy of the oculomotor nerve may cause limited eye movements resulting in outward deviation of the eye, failure of the indirect pupillary response to light, ptosis and/or mydriasis.

#### Cranial nerve palsy IV:

The fourth cranial nerve is called the trochlear nerve. Palsy of the trochlear nerve causes failure of downward and inward eye movements.

**Cranial nerve palsy V:**The fifth cranial nerve is called the trigeminal nerve and consists of three major branches. Palsy of the ophthalmic nerve (V1) results in sensory loss of the upper 1/3 part of the face including the nose, forehead and eye (including corneal reflex). Palsy of the maxillary nerve (V2) results in sensory loss of the middle 1/3 part of the face. Palsy of the mandibular nerve may results in sensory loss of the lowest 1/3 part of the face, weakness of jaw clenching and /or an exaggerated jaw reflex.

#### Cranial nerve palsy VI:

The sixth cranial nerve is called the abducens nerve. Palsy of the abducens nerve causes failure of lateral eye movement.

#### Cranial nerve palsy VII:

#### The seventh cranial nerve is called the facial nerve. Palsy of the facial nerve causes facial weakness resulting in asymmetrical expression.

#### Cranial nerve palsy VIII:

The eighth cranial nerve is called the vestibulocochlear nerve. Palsy of the vestibulocochlear nerve may cause hearing loss and/or vestibular symptoms.

#### Cranial nerve palsy IX:

#### The ninth cranial nerve is called the glossopharyngeal nerve. Palsy of the glossopharyngeal nerve causes a weakened or absent gag reflex.

#### Cranial nerve palsy X:

The tenth cranial nerve is called the vagus nerve. Palsy of the vagus nerve may result in hoarseness, dysphagia, dyspnoea and/or asymmetrical elevation of the soft palace with uvular deviation away from the affected side.

#### Cranial nerve palsy XI:

The eleventh cranial nerve is called the spinal accessory nerve. Spinal accessory nerve palsy results in weakness of the sternocleidomastoid and/or trapezius muscles.

#### Cranial nerve palsy XII:

The twelfth nerve is called the hypoglossal nerve. Hypoglossal nerve palsy results in deviation of the tongue towards the side of the lesion and atrophy of the affected side may be present.

#### Other findings

Indicate ‘Yes’ only if stated in the patient’s medical record. Indicate ‘No’ if the record confirms there are no other findings or if no mention of other findings is present within a complete medical record. If a significant number of reports are missing from patient’s medical record so that physical examination may not be assessed, select ‘Unknown’.

Describe any other relevant findings. Please write in English and avoid the use of abbreviations for clarity.

**NB: Save the CRF after completing!**

# CRF 3 - Data concerning “Diagnosis and Imaging”

Diagnosis & Imaging at diagnosis has been split up in 7 different sections: MRI Brain, MRI Spine, MR Spectroscopy, PET, CT, Biopsy and CSF cytology.

Data from the original radiologic reports (MRI and if available advanced imaging techniques) at time of diagnosis will be collected. Only if a certain report is available, i.e. if the answer to the question “Was an MRI/PET/CT/Biopsy/CSF performed at diagnosis” is “yes” additional questions will appear.

When tumour samples are available from biopsy at time of diagnosis, the registry collects data from the existing local pathology reports. In the future, we hope to centrally collect the actual samples for central (digital) pathology review by experts in the field of neuro-oncology.

## MRI brain

#### Was an MRI of the brain performed at diagnosis?

This refers to date of initial imaging diagnosis. Indicate ‘yes’ if MRI record is on file or there is a report of an MRI procedure at the time of diagnosis available in the patient’s medical record. Indicate ‘no’ if no record exists that an MRI was collected during diagnosis or a CT was collected instead. If a significant number of reports are missing from the patient’s medical record so that no clear assessment can be made of an MRI procedure being done, select ‘Unknown’

#### Date of imaging

Date of MRI can be found in MRI report or patient’s medical record.

**NB: Save the CRF after completing!**

## MRI spine

#### Was an MRI spine performed at diagnosis?

This refers to date of initial imaging diagnosis. Indicate ‘yes’ if MRI spine record is on file or there is a report of an MRI spine procedure at the time of diagnosis available in the patient’s medical record.

Indicate ‘no’ if no record exists that an MRI spine was collected during diagnosis or a CT was collected instead. If a significant number of reports are missing from the patient’s medical record so that no clear assessment can be made of an MRI spine procedure being done, select ‘Unknown’

#### Date of imaging

Date of MRI spine can be found in MRI spine report or patient’s medical record.

**Did the MRI report state evidence of disease in the spine?**

Indicate ‘yes’ if the MRI report states there is evidence of disease in the spine. Indicate ‘no’ if the MRI report states there is no evidence of disease in the spine. Indicate ‘Unknown’ if the record confirms spine MRI was performed; however, the report and results are unavailable.

**NB: Save the CRF after completing!**

## MR Spectroscopy

#### Was MR spectroscopy completed at diagnosis?

Indicate ‘yes’ if MR Spectroscopy record is on file or there is a report of an MR Spectroscopy procedure at the time of diagnosis available in the patient’s medical record. Indicate ‘no’ if no record exists that an MR Spectroscopy was collected during diagnosis. If a significant number of reports are missing from the patient’s medical record so that no clear assessment can be made of an MR Spectroscopy procedure being done, select ‘Unknown’.

#### Date of imaging

Date of MR Spectroscopy can be found in MR spectroscopy report or patient’s medical record.

**NB: Save the CRF after completing!**

## PET

#### Was a PET completed at diagnosis?

This refers to date of initial diagnosis. Indicate ‘yes’ if PET record is on file or there is a report of a PET procedure at the time of diagnosis available in the patient’s medical record. Indicate ‘no’ if no record exists that a PET was collected during diagnosis. If a significant number of reports are missing from the patient’s medical record so that no clear assessment can be made of a PET procedure being done, select ‘Unknown’.

#### Date of imaging

Date of PET can be found in PET report or patient’s medical record.

**NB: Save the CRF after completing!**

## CT

#### Was a CT completed at diagnosis?

This refers to date of initial diagnosis. Indicate ‘yes’ if CT record is on file or there is a report of a CT procedure at the time of diagnosis available in the patient’s medical record. Indicate ‘no’ if no record exists that a CT was collected during diagnosis. If a significant number of reports are missing from the patient’s medical record so that no clear assessment can be made of a CT procedure being done, select ‘Unknown’.

#### Date of imaging

Date of CT can be found in CT report or patient’s medical record.

**NB: Save the CRF after completing!**

## Biopsy

#### Was patient’s diagnosis confirmed by biopsy?

This refers to any point at which a biopsy was performed that provided a confirmation of diagnosis. This may be found in pathology or operative notes. If pathology is unavailable, patient’s medical record may be used.

Indicate “Yes” only if the patient’s medical record states that diagnosis was confirmed by biopsy.

Indicate “No” if the patient record states a biopsy was not performed or if a performed biopsy was not diagnostic. Additionally, if no mention of a biopsy is available within a complete record it is assumed to have not been performed. Indicate “Unknown” if record confirms a biopsy was performed; however the results are unavailable. If a significant number of reports are missing from patient’s medical record so that no clear assessment of biopsy being performed can be made, select “Unknown”.

#### Reason for biopsy

The reason for biopsy is subdivided into various classes: Confirmation of diagnosis, Study related, Other (and specify) . The reason for biopsy can be found in operative or pathology notes, or patient’s medical record. If the reason for biopsy is not ‘confirmation of diagnosis’ or ‘study related’, please indicate ’Other’ and specify. Indicate ’Unknown’ if record confirms a biopsy was performed; however the intent and/or results are unavailable.

#### Location of biopsy

This may be found in operative or pathology notes or patient’s medical record.

Please write in English and avoid the use of abbreviations for clarity. Specify as ’Unknown’ if record confirms a biopsy was performed; however the location is unavailable.

#### Number of biopsies

Indicate the total number of samples collected during biopsy. This may be found in operative or pathology notes or patient’s medical record. Indicate ’Unknown’ if record confirms a biopsy was performed; however the number of biopsies are unavailable.

#### Pathology diagnosis

Indicate the confirmed diagnosis. This may be found in pathology report of biopsy or in patient’s medical record. The pathology diagnosis is subdivided into various classes: Pilocytic astrocytoma, Fibrillary astrocytoma, Anaplastic astrocytoma, Glioblastoma multiforme, Oligodendroglioma, PNET, Ependymoma. Indicate ‘Other + Specify’ if the diagnosis from biopsy is different from the options described above. In case of ‘Other’, also provide a specification. Indicate “Unknown” if record confirms a biopsy was performed; however the diagnosis is unavailable.

#### WHO grading

Please specify the WHO grading for diagnosis. This may be found in pathology report of biopsy or in patient’s medical record. WHO grading is subdivided into various classes: Grade I, Grade II, Grade III, Grade IV. Indicate “Unknown” if record confirms a biopsy was performed; however grading is unavailable.

#### Reported complications

This refers to all reported complications due to the biopsy. This may be found in operative or clinic notes. Please write in English and avoid the use of abbreviations for clarity.

**Mutation analysis done?**

#### For H3F3A (H3.3K27M), HIST1H3B/C/I (H3.1K27M), ACVR1, TP53, ATRX, DAXX, TERT, Other:

Indicate ‘Yes’ only if a mutation analysis is confirmed in the patient’s medical record. Indicate ‘No’ if the record confirms no mutation analysis or if no mention of a mutation analysis is present within a complete medical record. If a significant number of reports are missing from patient’s medical record

so that patient history may not be assessed, select ‘Unknown’.

**Specify**
In case a mutation analysis other than for H3F3A (H3.3K27M), HIST1H3B/C/I (H3.1K27M), ACVR1, TP53, ATRX, DAXX or TERT was done, specify the mutation of interest. Please write in English and avoid the use of abbreviations for clarity.

**Mutation present?**

#### For H3F3A (H3.3K27M), HIST1H3B/C/I (H3.1K27M), ACVR1, TP53, ATRX, DAXX, TERT, Other:

Indicate ‘Yes’ only if mutation is confirmed to be present in the patient’s medical record. Indicate ‘No’ if the record confirms mutation is not present. If a significant number of reports are missing from patient’s medical record so that results of mutation analyses may not be assessed, select ‘Unknown’.

**NB: Save the CRF after completing!**

## CSF cytology

#### Was CSF cytology done at diagnosis?

Indicate “Yes” if the patient record states that CSF was collected at the time of diagnosis. Indicate “No” if the record confirms CSF was not collected, CSF was collected but not at diagnosis, or if there is no mention of a procedure in the patient record. If there is no mention within a complete record it is assumed to have not been performed. Indicate “Unknown” if the record confirms a procedure; however, there is no date associated with the collection that can be linked to time of diagnosis.
Also, if a significant number of reports are missing from patient’s medical record so that patient history may not be assessed, select ‘Unknown’.

#### Site of fluid

The site of fluid collection will be directly stated in the pathology report, lab notes or patient’s medical record. There are different ways to get a sample of CSF. Lumbar puncture (spinal tap) is the most common method. CSF may also be collected from a tube that's already placed in the fluid, such as a shunt or a ventricular drain. Indicate “Lumbar” if collection was performed by lumbar puncture . Indicate “Ventricular” if collection taken from a shunt or a ventricular drain. Indicate “Unknown” if there is a record of collection; however, no site associated with the collection.

#### Was the CSF positive for tumour cells?

This pertains only to the CSF collection at time of diagnosis. It will be directly stated in the pathology report, lab notes or patient’s medical record. Indicate “Yes” if the patient record states that CSF was positive for tumour cells or disease at the time of diagnosis. Indicate “No” if the record confirms CSF was not positive for tumour cells/disease or if there is no mention of tumour cells/disease in the patient record. If there is no mention within a complete record it is assumed to have been negative.

Indicate “Unknown” if there is a record of collection; however, there is no result described associated with the collection.

**NB: Save the CRF after completing!**

# CRF 4 - Data concerning “Treatment”

Data collection concerning treatment regimen includes radiotherapy, chemotherapy regimens, surgical interventions and steroid application. Dates, types and doses of therapy will be collected.

Each period of treatment, either started:

- at diagnosis or
- at any other evaluation moment leading to a change in the initially proposed therapy or leading to a new therapy after the preceding therapy was ended as planned

is represented by a ‘Therapy number’.

For every such period of treatment a new Treatment record should be created indicated by a unique and sequential ‘Therapy number’.

For each period of treatment you want to add, go to the ‘Create’-button, fill in the next consecutive ‘Therapy number’. Also fill in the start date of the treatment period to be added (at ‘Date’).

#### Date Start date of treatment period should be equal to the earliest start date of a treatment modality.

Then, for this treatment period, start answering the questions for each treatment modality by clicking on ***each*** of the treatment modality tabs (e.g. Radiotherapy, Chemotherapy, Surgery, Steroids).

Only if a certain treatment modality was applied in the indicated treatment period, i.e. if the answer to the question “Was the patient treated with….?” or “Did the patient undergo..........?” is “yes” the rest of the CRF will be shown.
Once all the relevant questions related to the treatment modalities have been answered, click the ‘Save’-button.

Create a new Treatment record for each period of treatment that was started.

A list of all Treatment records created for a patient will appear on the screen. You can sort this list by Therapy number and Treatment Date by clicking on the column header.

You can also save data for each modality separately. But then, after saving one modality, you will have to select the Therapy number from the list of Treatment records and start adding data for the next modality, etc.

## Radiotherapy

#### Was the patient treated with radiotherapy?

This refers to radiotherapy in any amount throughout the treatment period/ treatment number being added. Examine clinic notes for any mention of radiation (RT, RTx, XRT) and/or a protocol regimen that includes radiation as treatment.

Indicate “Yes” if the record confirms radiation was used as treatment. Indicate “No” if the record confirms radiation was not used or no radiation is mentioned in a complete record. If a significant number of reports are missing from a record so that patient history may not be assessed select “Unknown”.

#### Start

This refers to the date a radiation regimen started.

#### Stop

This refers to the date a radiation regimen was completed.

#### Type of radiation

Examine clinic notes for any mention of radiation (RT, RTx, XRT) and/or a protocol regimen that includes radiation as treatment. Specify radiation type only if confirmed by the patient record. The types of initial radiotherapy regimens are subdivided into three classes:

- 1. Hypofractionated radiation is divided into large doses; not given daily. Typically provided over 3 to 4 weeks.
  2. Normofractionated radiation is divided into normal doses. The conventional dose of radiation ranges between 54 Gy and 60 Gy given locally to the primary tumour site in single daily fractions. This is considered standard of care. (e.g. 54 Gy in single doses of 1.8 Gy given once daily over 6 weeks).
  3. Hyperfractionated radiation is divided into small doses and treatments that are given more than once a day. Patients receive higher doses of radiation (e.g. 70.2 Gy) over 6 weeks.

Indicate “Unknown” if radiation was received during treatment; however, the type of radiation cannot be accurately determined.

#### Total dose (Gy)

Total Dose is indicated in Gy (Gray) ( e.g. 54 Gy). This information may be found in patient’s medical record. If applicable a protocol may provide specifics if not found in the patient record.

Indicate ‘Unknown’ if the record states radiation was given; however, total dose cannot be retrieved.

#### Total number of fractions

The total dose of radiation is divided into fractions (amount given in a single administration, e.g. 54 Gy in 1.8 Gy fractions). This information may be found in clinic and treatment notes. If applicable a protocol may provide specifics if not found in the patient record.

Indicate ‘Unknown’ if the record states radiation was given; however, number of fractions cannot be retrieved.

#### Did the patient receive local radiation only?

Local radiation refers to radiation limited to the tumour area.

Indicate “Yes” if the record confirms local radiation. If not confirmed by the patient record, assume radiation is local or focal as this is the current standard of care. Indicate “No” if the record confirms expanded radiation. Indicate “Unknown” if radiation was received during treatment; however, the area of radiation was not accurately specified.

**NB: Save the CRF after completing!**

**NB.** Cases of a re-irradiation need to be added by creating a new Treatment period/Therapy number.

## Chemotherapy

#### Was the patient treated with chemotherapy?

Examine to labs, clinic notes, treatment notes, and notes on clinical protocols to determine if the patient received chemotherapy (e.g. CH, CMT, CTX, CRT) at any point during treatment. If a significant number of reports are missing from a record so that patient history may not be assessed select “Unknown”.

#### Was the patient included in a clinical trial?

Indicate ‘yes’ if the patient record confirms that patient is included in a clinical trial. Indicate “No” if the record confirms patient is not included in a clinical trial or no clinical trial is mentioned in a complete record. If a significant number of reports are missing from a record so that patient history may not be assessed select “Unknown”.

#### Please specify protocol

This refers to the treatment procedures used in a clinical trial. Specify protocol name. Write “Unknown” if the record confirms that chemotherapy was received; however, no information about the protocol is available.

**Before being able to add more details about a given chemotherapy regimen you need to save the entries in the Chemotherapy screen first. After saving those data search for the correct record in the list of Therapy numbers/Treatment Dates. Click on the corresponding “Pencil” to start editing, e.g. adding drugs to the Chemotherapy screen.**

**A ‘Create’-button will have appeared on the Chemotherapy screen.**

**To add more information about the chemotherapy regimen, please click “Create”.**

**A separate entry screen will pop up in which you can enter details for the first drug.**

#### Drug

Refers to the name of drugs used as chemotherapy. Specify drug name.

#### Administration type

This refers to the mode of administration of drugs. The types of administration are subdivided into three classes; oral chemotherapy, intravenous chemotherapy, oral and intravenous chemotherapy. This may be found in clinic notes, treatment notes, and notes on clinical protocols. Indicate “Unknown” if the record confirms that chemotherapy was received; however, no information about the administration type is available.

#### Cumulative dose (mg)

Depending on the drug(s) to be given, there are different ways to determine chemotherapy doses. Most chemotherapy drugs are measured in milligrams (mg). The overall dose may be based on a person’s body weight in kilograms (1 kilogram is 2.2 pounds). For instance, if the standard dose of a drug is 10 milligrams per kilogram (10 mg/kg), a person weighing 110 pounds (50 kilograms) would receive 500 mg (10 mg/kg x 50 kg). Some chemotherapy doses are determined based on body surface area (BSA), expressed in meters squared (m^2^). Examine clinic and treatment notes for any mention of chemotherapy used as treatment to determine the dosage for a given chemotherapy regimen. If applicable a protocol may provide specifics if not found in the patient record.

Indicate “Unknown” if the record confirms that chemotherapy was received; however, no information about cumulative dose is available.

#### Number of cycles received

Chemotherapy may repeat weekly, bi-weekly, or monthly. Usually, a cycle is defined in monthly intervals. For example, two bi-weekly chemotherapy sessions may be classified as one cycle. In most cases, the number of cycles - or the length of chemotherapy from start to finish - has been determined by research and clinical trials. Examine clinic and treatment notes for any mention of chemotherapy used as treatment to determine the number of cycles for a given chemotherapy regimen. If applicable a protocol may provide specifics if not found in the patient record.

Indicate “Unknown” if the record confirms that chemotherapy was received; however, no information about number of cycles is available.

#### Start date

Refers to the date of first administration of the drug.

#### Stop date

Refers to the date a patient received the last gift of the drug.

#### Timing of chemotherapy

“Timing” refers to the point at which chemotherapy was provided in relation to the timing of other methods of treatment, as well as stage of disease in relation to stages of treatment. The timing of chemotherapy is subdivided into various classes.

- Indicate “Neoadjuvant” if chemotherapy is given before primary therapy.
- Indicate “During RT” (radiation) if chemotherapy is given in conjunction with radiotherapy.
- Indicate “Adjuvant” if chemotherapy is given after primary therapy.
- Indicate “Progressive” if chemotherapy is given in response to an observed progression of disease.
- Indicate “Refractory” if chemotherapy is provided as treatment to disease that has not responded or has become resistant to treatment. May also be called resistant cancer.
- Indicate “Unknown” if the record states that chemotherapy was received; however, not enough information exists in the patient record to determine timing.

**NB: Save the CRF after completing!**

**In case of a second drug, restart the procedure as described above: click “Create” and start answering the questions for the next drug.**

## Surgery

#### Did the patient undergo surgical intervention?

This may be found in pathology, operative or clinic notes.

Indicate “Yes” only if the patient’s medical record states brain specific surgery was performed. Indicate “No” if the patient’s medical record states no surgery was performed or if the patient’s medical record shows no evidence of brain specific surgery. It is assumed to have not been performed if no report is available within a complete record. If a significant number of reports are missing from a record so that no accurate and reliable assessment can be made, select “Unknown”.

#### Date of surgery

This refers to the date surgery was performed and may be found in patient’s medical record: pathology, operative or clinic notes.

#### Type and extent of surgery (if available, confirm with post-operative imaging):

For type and extent of surgery there are four answer options: Shunt, Biopsy (10%), Incomplete resection (10-90%) and Other. This may be found in patient’s medical record: pathology, operative or clinic notes. Specify only if confirmed in patient’s medical record. Indicate “Unknown” if record confirms a surgical procedure was performed; however, there is no type and/or extent specified with the report.

#### If other, please comment

Other refers to any other type and/or extent of surgery not mentioned in the list of choices. Please add any additional information necessary.

#### Did the patient have obstructive hydrocephalus?

This refers to the presentation of obstructive hydrocephalus at point of surgery. This may be found in patient’s medical record: clinic notes, imaging, pathology, or operative reports.
Indicate “Yes” if patient’s medical record clearly confirms that the patient presented with hydrocephalus. Indicate “No” if patient’s medical record confirms the patient did not present with hydrocephalus or there is no mention of hydrocephalus in a patient record. It is assumed to have not been present if no mention is available in a complete record. If a significant number of reports are missing from a record so that no accurate and reliable assessment can be made, select “Unknown”.

#### Did the patient undergo a CSF diversion?

If the patient’s medical record confirms a procedure was performed to drain fluid from the brain by means of surgical intervention to address hydrocephalus select the type of procedure completed. For type of ventriculostomy there are various answer options: No, Third ventriculostomy, External ventricular drain, Ventriculo-peritoneal shunt, Unknown. This may be available in the patient’s medical record; operative reports, imaging reports, clinic notes, etc.

Indicate “No” if the record confirms no procedure was performed or if the patient record shows no evidence of a procedure. It is assumed to have not been performed if no report is available in a complete record.

If a surgical procedure was performed; however, the type of procedure is unavailable select “Unknown”.

**NB: Save the CRF after completing!**

## Steroids

#### Did patient receive steroids?

This question examines if the patient received steroids in any amount throughout the indicated period of treatment/Therapy number. This may be found in patient’s medical record: clinic notes, treatment section, etc.

Indicate “Yes” if patient’s medical record clearly confirms that the patient was treated with steroids.

Indicate “No” if patient’s medical record confirms the patient was not treated with steroids or there is no mention of steroid use in a patient record. It is assumed to have not been used if no mention is available in a complete record.

If a significant number of reports are missing from a record so that no accurate and reliable assessment can be made, select “Unknown”.

#### When were steroids tapered?

This refers to the date that a steroid regimen (during initial radiotherapy or re-irradiation) taper began. Tapering refers to the gradual decrease of steroids given to a patient. You need to compare radiation therapy timeline with steroid record to determine when taper began. Full taper does not have to be completed to be applicable.

For timing of steroid tapering there are various answer options: Never, <3 weeks after start RT, 3-6 weeks after start RT, <4 weeks after end of RT, >4 weeks after end of RT, Unknown and Not applicable.

Indicate “Never” if patient received steroids during radiotherapy; however, no intent to taper ever occurred. Indicate “Unknown” if the record states that there was intent to taper steroids during radiotherapy; however, taper cannot be confirmed. Indicate “Not Applicable” if steroids were not given during radiotherapy.

**Before being able to add more details about steroids given you need to save the entries in the Steroids screen first. After saving those data search for the correct record in the list of Therapy numbers/Treatment Dates. Click on the corresponding “Pencil” to start editing, e.g. adding steroids to the Steroids screen.**

**A ‘Create’-button will have appeared on the Steroids screen.**

**To add more information about the steroids given, please click “Create”.**

**A separate entry screen will pop up in which you can enter details for the first steroid .**

#### Drug

Refers to the name of the steroid used. Specify drug name.

#### Cumulative dose (mg)

Cumulative dose should be given in mg.

Indicate ‘Unknown’ if the record states that a specific steroid was given ; however, dose cannot be retrieved.

#### Start date

Refers to the date that a steroid regimen started.

#### Stop date

Refers to the date that a steroid regimen was completed.

**NB: Save the CRF after completing!**

**In case of a second steroid, restart the procedure as described above: click “Create” and start answering the questions for the next steroid.**

# CRF 5 - Data concerning “Response evaluation”

Response evaluation includes clinical and/or radiological evaluation. Clinical evaluation includes reported signs/symptoms and findings on physical and neurological examination. Neuro-imaging to be collected include MRIs.

Data of clinical and/or radiological response evaluation are collected at the following time points or events (if available):

- at time of best response after each therapy, and
- at time of progression.

For each Response evaluation time point that you want to add, go to the ‘Create’-button and fill in ‘Date’. For ‘Date’ use the earliest date of the evaluations (clinical or imaging) done for that particular Response evaluation time point.

Then start answering the questions for each evaluation type by clicking on the tabs (e.g. Clinical Response Evaluation, MRI/CT).

Only if a certain evaluation type was applied in relation to the indicated Response evaluation time point, i.e. if the answer to the question “Did the patient have..........?” or “Was an MRI/CT completed….?” is “yes” the rest of the CRF will be shown. Once all the relevant questions related to the type(s) of evaluation have been answered, click the ‘Save’-button.

A list of all Response Evaluation records created for a patient will appear on the screen. You can sort this list by Date by clicking on the column header.

Create a new Response Evaluation record for each Response evaluation time point that is considered to be of relevance.

## Clinical response evaluation

#### Did the patient have a clinical response evaluation?

This refers to a physical evaluation of the patient to assess symptoms, response to treatment, or reactions to therapy. Examine patient’s medical record to identify any physical evaluation associated with the designated time point or associated imaging.

Indicate “Yes” if the patient’s physical status was evaluated at the designated time point or an evaluation was performed in conjunction with time point imaging.

Indicate “No” if the report confirms the patient’s physical status was not evaluated at the designated time point or no report is available in a complete record.

If a significant number of reports are missing from a record so that patient history may not be assessed select “Unknown”.

#### What was the patient’s neurological response?

Refers to the patient’s physical status reported at designated time point. The neurological response is therefore subdivided into various classes: Progression, Stable disease/Unchanged neurology- Partial response, Complete resolution of neurological symptoms.

- “Progression” refers to the presence of new observed symptoms, worsening of prior symptoms, and/or general deterioration of physical and neurological status.
- “Stable disease/unchanged neurology” refers to observed symptoms that are neither decreasing nor increasing in extent or severity.
- “Partial response” refers to an improvement in observed symptoms related to treatment.
- “Complete Resolution” refers to the disappearance of all associated symptoms.

Indicate “Unknown” if the report confirms the patient did receive a physical evaluation; however, the results are unknown or unavailable.

#### Date of clinical response evaluation

Refers to clinical evaluation that corresponds with the designated time point or associated imaging. Clinical evaluation and associated imaging may be completed on different dates.

#### Clinical evaluation findings

Specify all signs and symptoms reported by patient or patient’s parents of guardians and all findings from physical and neurological examination at designated time point of evaluation stated in patient’s medical record.

**Weight (kg)**

Patient weight should be assessed in patient’s medical record. Patient weight should be rounded to the nearest whole number if necessary.

If weight at date of clinical response evaluation is not available, select ‘Unknown’.

**Height (cm)**

Patient height should be assessed in patient’s medical record. Patient height should be rounded to the nearest whole number if necessary.

If height at date of clinical response evaluation is not available, select ‘Unknown’.

**NB: Save the CRF after completing!**

## MRI/CT

#### Was an MRI/CT completed at response evaluation time point?

This refers to MRI/CT that corresponds with the designated time point.

Indicate “Yes” if an MRI/CT was performed and can be associated with the designated time point.

Indicate “No” if no reports confirm no MRI/CT was performed or if no image can be associated with the designated time point. If a significant number of reports are missing from a record so that patient history may not be assessed select “Unknown”.

**Type of image obtained at evaluation**

This refers to the type of image obtained at response evaluation time point; magnetic resonance imaging (MRI) or computerized tomography (CT scan). Indicate “MRI” if an MRI was obtained at response evaluation time point. Indicate “CT” if a CT was obtained at response evaluation time point. If a significant number of reports are missing from a record so that type of imaging may not be assessed select “Unknown”.

#### Date of imaging

This refers to date of MRI/CT that corresponds with the designated time point. This can be found in MRI report or patient’s medical record.

#### Was metastatic disease reported in the brain?

This refers to the MRI/CT that corresponds with the designated time point. Indicate “Yes” only if the image report clearly states that metastatic disease was observed in the brain. Indicate “No” if the image report states no metastatic disease was observed or if a complete report makes no mention of metastatic disease. Indicate “Unknown” if an image is associated with the time point; however, the image report and/or results are unavailable.

#### Was metastatic disease reported in the spine?

This refers to the MRI/CT that corresponds with the designated time point. Indicate “Yes” only if the image report clearly states that metastatic disease was observed in the spine. Indicate “No” if the image report states no metastatic disease was observed in the spine or if a complete report makes no mention of metastatic disease in the spine. Indicate “Unknown” if an image is associated with the time point; however, the image report and/or results are unavailable.

#### Was a new or worsening of prior hydrocephalus present?

This refers to the observed presence of new or worsening hydrocephalus at the designated time point. Indicate “Yes” if the record confirms new or worsening hydrocephalus is observed at the designated time point. Indicate “No” if the record confirms no hydrocephalus is observed, no mention of hydrocephalus is made, or if previously reported hydrocephalus remains stable. Indicate “Unknown” if hydrocephalus is reported; however it cannot be determined when hydrocephalus was first observed. If a significant number of reports are missing from a record so that patient history may not be assessed select “Unknown”.

#### Did the MRI/CT report state evidence of relapse or progression of disease?

#### This refers to MRI/CT that corresponds with the designated time point.

- “Relapse” refers to the return of disease after a period of improvement.
- “Progression” refers to the worsening or spread of disease within the body.

Indicate “Yes” only if the image report clearly states that relapse or progression of disease was observed. Indicate “No” if the image report states no relapse or progression of disease was observed or if a complete report makes no mention of relapse or progression. Indicate “Unknown” if an image is associated with the time point; however, the image report and/or results are unavailable.

**Specify the type of tumour relapse or progression.**

#### This refers to MRI/CT that corresponds with the designated time point.

#### For the purposes of this question “Progressive disease” refers to any observable worsening, growth, and/or spread of disease while not receiving treatment.

#### “Refractory” refers to cancer that does not respond to treatment. The cancer may be resistant at the beginning of treatment or it may become resistant during treatment. Also called resistant cancer.

#### “Recurrent” refers to cancer that has recurred (come back), usually after a period of time during which the cancer could not be detected. The cancer may come back to the same place as the original (primary) tumour or to another place in the body. Also called recurrence.

#### “Second Primary” refers to a new primary cancer in a person with a history of cancer.

#### What degree of response was confirmed in the MRI/CT report?

#### This refers to MRI/CT that corresponds with the designated time point and refers to any documented measurable improvement or decrease in lesion size related to treatment observed via imaging.

- Minor Response (MR): "Minor response" roughly means a small amount of shrinkage. Minor response is not really a standard term but is increasingly used. Roughly speaking, a minor response is more than 25% of total tumour volume but less than the 50%.
- Partial response (PR): “Partial response refers to a decrease of 50% or more in the sum of total tumour volume.

#### Complete response (CR): “Complete response” refers to the disappearance of all target lesions in response to treatment. This does not always mean the cancer has been cured. Also called complete remission.

#### Indicate “Unspecified” if the report confirms a response; however, the degree of response cannot be determine or is unavailable. Indicate “Not applicable” if no response was observed or reported.

**NB: Save the CRF after completing!**

# CRF 6 - Data concerning “Follow up”

Survival follow up and/or death will be registered in the Follow Up screens.
For each follow up moment that you want to add, click the ‘Create’-button. An entry screen with follow up data will appear.

A list of all Follow up records created for a patient will appear on the screen. You can sort this list by Follow Up Date by clicking on the column header.

#### Date of follow up

Refers to last available documented contact with the patient or patient’s parents or guardians. This may be MRI/CT report, clinic note, phone record, hospice record, or death note.
If patient is deceased ‘Date of follow up’ should be the date of death.

#### Is the patient alive?

Refers to the patient’s status during evaluation at last available documented contact.

Indicate “Yes” if the record confirms the patient is alive at time of physical evaluation associated with final time point. Indicate “No” if the record confirms the patient is deceased at time of physical evaluation associated with final time point.

#### Date deceased

Refers to the confirmed date of death. This may be found in patient’s medical record, death note, or autopsy report.

#### Cause

Cause of death may be found in final clinic note, death note, or autopsy report. If confirmed in the patient’s medical record, indicate cause of death stated within the report. Cause of death is divided into various classes: Disease related, Treatment related, Progression of next malignancy, Other and Unknown.
Indicate “Unknown” if patient’s death was confirmed but cause of death could not be retrieved from patient’s medical record, death note or autopsy report or cause of death was unclear.

**Specify cause of death**

Include any relevant specification to cause of death mentioned in the patient’s medical record.

#### If deceased, was an autopsy performed?

May be found in final patient’s medical record, death note, or autopsy report.

Indicate “Yes” if the record confirms an autopsy was performed. Indicate “No” if the record confirms an autopsy was not performed. If a significant number of reports are missing from a record so that patient history may not be assessed select “Unknown”.

**NB: Save the CRF after completing!**

***! CONGRATULATIONS, YOU HAVE SUCCESFULLY ENTERED A PATIENT TO THE SIOPE DIPG REGISTRY!***
