# Supplementary material for: Development of the SIOPE DIPG network, registry and imaging repository: a collaborative effort to optimize research into a rare and lethal disease
Source: J Neurooncol. 2017 Jan 21;132(2):255–66. doi: 10.1007/s11060-016-2363-y (PMC5378734; doi:10.1007/s11060-016-2363-y)
Supplement: Supplementary file 2 — Supplementary material 2 (DOCX 95 KB) [file 11060_2016_2363_MOESM2_ESM.docx]

**Development of the SIOPE DIPG Network, Registry and Imaging Repository:**

**A collaborative effort to optimize research into a rare and lethal disease.**

Journal of Neuro-Oncology

*Sophie E.M. Veldhuijzen van Zanten, Joshua Baugh and Brooklyn Chaney, Dennis De Jongh, Esther Sanchez Aliaga, Frederik Barkhof, Johan Noltes, Ruben De Wolf, Jet Van Dijk, Antonio Cannarozzo, Carin M. Damen-Korbijn, Jan A. Lieverst, Niclas Colditz, Marion Hoffmann, Monika Warmuth-Metz, Brigitte Bison, David T.W. Jones, Dominik Sturm, Gerrit H. Gielen, Chris Jones, Esther Hulleman, Raphael Calmon, David Castel, Pascale Varlet, Géraldine Giraud, Irene Slavc, Stefaan Van Gool, Sandra Jacobs, Filip Jadrijevic-Cvrlje, David Sumerauer, Karsten Nysom, Virve Pentikainen, Sanna-Maria Kivivuori, Pierre Leblond, Natasha Entz-Werle, Andre O. von Bueren, Antonis Kattamis, Darren Hargrave, Péter Hauser, Miklos Garami, Halldora Kristin Thorarinsdottir, Jane Pears, Lorenza Gandola, Giedre Rutkauskiene, Geert O. Janssens, Ingrid K. Torsvik, Marta Perek-Polnik, Maria João Gil-da-Costa, Olga Zheludkova, Liudmila Shats, Ladislav Deak, Lidija Kitanovski, Ofelia Cruz, Andres Morales La Madrid, Stefan Holm, Nicolas Gerber, Rejin Kebudi, Richard Grundy, Enrique Lopez-Aguilar, Marta Zapata-Tarres, John Emmerik, Tim Hayden, Simon Bailey, Veronica Biassoni, Maura Massimino, Jacques Grill, William P. Vandertop, Gertjan J.L. Kaspers, Maryam Fouladi, Christof M. Kramm, Dannis G. van Vuurden on behalf of the members of the SIOPE DIPG Network.*

**Corresponsing author: Sophie E.M. Veldhuijzen van Zanten, VU University Medical Center Department of Pediatrics, Division of Oncology-Hematology, s.veldhuijzen@vumc.nl**


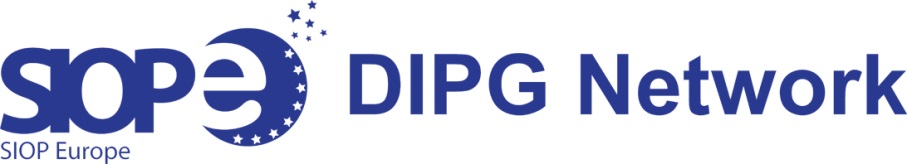


Bylaws of the SIOPE DIPG Network

The SIOPE Diffuse Intrinsic Pontine Glioma (DIPG) Network, hereinafter “DIPG Network”, is a sub-committee of the high-grade glioma (HGG) working group of the Brain Tumour Group (BTG) of the International Society of Paediatric Oncology Europe (SIOPE) committed to supporting and fostering the mission of the Society.

The DIPG Network is composed of paediatric oncologists, paediatric neurologists, radiotherapists, biologists, institutions and others motivated to carry out excellent clinical and biological research in the field of DIPG and collaborate with colleagues around the world.

Mission

The mission of the DIPG Network will be to serve as a facility for the design and execution of high quality, international multi-centre clinical and preclinical DIPG studies, intended to enhance the understanding of DIPG and to improve outcome of patients suffering from DIPG.

The DIPG Network also will be committed to foster investigator-initiated collaborative studies and promote and promulgate DIPG research internationally.

Aims

1. To initiate and expand an international DIPG Registry (which includes a DIPG Imaging Repository), hereafter “DIPG Registry”
2. To promote preclinical research in DIPG biology and *in vitro* and *in vivo* models, using the DIPG Registry
3. To enhance and facilitate high-quality investigator-initiated clinical studies in DIPG, with uniform in- and exclusion criteria, response criteria and endpoints.
4. To investigate clinically-relevant questions drawing from both bedside and laboratory observations.
5. To serve as a resource for professionals and patients and their parents

Core values

1. Collaboration and collegiality
2. Honesty
3. Support
4. Trust
5. Innovation
6. Promotion and development of scientific thought
7. Integrity
8. Accountability to our patients (and parents) and the scientific community

Structure

The DIPG Network is comprised of an executive committee, a group of scientific advisors, National Coordinators (NC) and non-NC members.

Below is a description of the duties and obligations of each component of the DIPG Network.

Executive Committee

The Executive Committee will manage and control the DIPG Network and will abide by and enforce the mission and the core values of the DIPG Network. This committee also will provide for all the administrative management of the DIPG Network.

The members of the Executive Committee will consist of the following: Chair, three National Coordinators, a patient/parent representative, a neurosurgeon, a radiotherapist and the immediate-past Chair, who will be Vice Chair. All members of the Executive Committee have voting rights. The chair will be elected every 4 years. Voting of new Executive Committee members will be 2 years before / after election of the chair, to guarantee continuity. These officers will hold their positions for a period of 4 years. Any member of the DIPG Network can nominate itself for consideration to be Chair. All Executive Committee officers, except for the Vice Chair, will be elected by the members of the DIPG Network by anonymous voting.

The Chair’s duties include:

- the representation of the DIPG Network and its interests to third parties,
- chairing the meetings of the Executive Committee
- chairing all the General Meetings of the DIPG Network.

The Vice Chair will be in charge of coordinating all the DIPG Network meetings, correspondence on behalf of the DIPG Network, and the minutes and agenda for every meeting.

The Executive Committee tasks are:

- Draft the terms and conditions for submitting, reviewing and approving proposals for research projects, a draft of which is incorporated in Annex 1
- Draft the terms and conditions for managing, maintaining of and access to the DIPG Registry
- Deciding on research proposals submitted to the DIPG Network, including requests for use of data from the DIPG Registry
- Appointing the principal investigator to the DIPG Network initiated research
- Deciding on grant funding mechanisms
- Coordinating and review of publications resulting from proposals that involved any asset of the DIPG Network
- Assisting the Chair and Vice-Chair in executing their tasks
- Appointing scientific advisors
- Coordinate funding activities to support the DIPG Network
- Appointing DIPG Network Administrator(s) that may receive and administer DIPG Network Funding, including funding for a specific project
- Appoint other third parties to provide services for the DIPG Network and decide on the budget spent for such services.
- Any other tasks that may be assigned by the DIPG Network to the Executive Committee from time to time.
- To communicate with the rest of the DIPG Network by e-mailing the minutes of Executive Committee meetings and posting these on the SIOPE website.

Group of Scientific Advisors

The number of scientific advisors will vary depending on the needs of the DIPG Network and will be appointed on ad hoc basis. Such advisors will consist of individuals with expertise in areas such as: biostatistics and biometry, medical ethics and health policy, basic science research, translational research, (neuro)psychology and neuroimaging. Individuals with expertise in other areas not mentioned also can be added if deemed necessary by the DIPG Network Members. Scientific Advisors will be selected from nominations presented by members of the DIPG Network and will be retained provided they contribute to the development of the scientific mission of the DIPG Network. The Executive Committee shall have the right to terminate the involvement of any Scientific Advisor if it considers that such advisor no longer contributes to the mission and core values of the DIPG Network.

The Scientific Advisors

- offer honest and transparent advice to the Executive Committee in matters of development and implementation of research protocols including ideas for innovative studies that could be executed using the DIPG Network.
- advise the Executive Committee on research proposals submitted to the DIPG Network.
- Shall sign an advisory agreement, a model of which is attached as Annex 2.

National Coordinators

National Coordinators are those DIPG Network Members that coordinate collaboration between the DIPG Members in their country. NC’s are presented by the DIPG Network members in their own country and appointed by the Executive Committee.

The tasks of the National Coordinators are:

- Identify and select hospitals and scientific experts in their country, that are involved in the treatment of DIPG patients and that potentially may join the DIPG Network.
- Raise awareness of the existence of the DIPG Network and its mission to potential DIPG Network members
- Select sites from the DIPG Network that may participate in a particular research project, based on available subject population and technical facilities;
- Coordinate research projects which involve sites of DIPG Network Members on a national level
- Be intermediary between such sites and the principal investigator
- Be intermediary between the Executive Committee and the DIPG Network members in their country
- Submit data to the DIPG Registry via web-based CRF forms as facilitated by the Dutch Childhood Oncology Group (DCOG/SKION)
- Any tasks that may be assigned to them if necessary for the coordination of the DIPG Network activities in their region, subject to consultation, discussion and decision by the Members

Members

Members will be identified by DIPG Network National Coordinators’ recommendations. The Executive Committee will notify and subscribe the approved Member to the DIPG Network.

The Members’ duties shall include honest and transparent participation in research projects (such as including patients in the DIPG Registry) initiated by the DIPG Network in accordance with these Bylaws. The Executive Committee will consider for each research project a dedicated principal investigator. The Principal Investigator of the research proposal in consultation with the National Coordinators will determine which DIPG Network sites will and can participate in a particular research project. All sites will pledge to uphold the principles of Good Clinical Practice and follow all the necessary regulatory requirements. Once a Member agrees to participate in the DIPG Network, they are registered into the DIPG Network database and will be eligible to participate in research studies. Sites will be selected to participate in a research project based on having available adequate subject population and technical facilities needed to safely conduct the specific research. DIPG Network members are free to decide on whether they wish to participate in a research project on a case by case basis and at their sole discretion. The DIPG Network members shall elect the Executive Committee Chair and Executive Committee Members except for the Vice Chair. The Executive Committee shall have the right to terminate the membership of any person or site at any time if they consider that the member no longer contributes to the mission and core values of the DIPG Network.

Meetings and voting

The Executive Committee will meet quarterly via teleconferencing and the DIPG Network membership will meet bi-annually during a SIOPE DIPG Network meeting (February/March) and in conjunction with the annual SIOPE BTG meeting (June), either as ‘standalone’ meeting or in conjunction with the International Society of Pediatric Neuro-Oncology (ISPNO) meeting, or before the annual International Society of Pediatric Oncology (SIOP) meeting (October). The DIPG Network meeting will be planned before the scientific gatherings mentioned above. The Vice-Chair, in consultation with the Chair, will organize the meetings, keep and disseminate the minutes to the DIPG Network membership not later than 1 week after their occurrence.

The minutes will include a list of meeting attendees, the general organization of the conference and the discussions and voting that were undertaken. The quorum for the validity of the Executive Committee meetings will be 5 and for the bi-annual DIPG Network meetings will be 2/3 of the total number of participants to the DIPG Network, as stated in the DIPG Network member list, which is maintained by the Executive Committee and circulated with its minutes. All questions or decisions that arise at any of the meetings will be decided by a majority vote. In case of equality of ballots, the Chair will cast the deciding vote. The Vice-Chair can also call for an emergency meeting to discuss issues that cannot be postponed until the regularly-scheduled meetings.

The business for the Executive Committee meetings will include discussions on proposals submitted by investigators, decisions about membership applications, the general status of the DIPG Network, and financial reports prepared by DCOG. The business of the general DIPG Network meetings will include presentations of the general status, the status of the DIPG Registry and DIPG Network updates by the Chair, research proposals by the Principal Investigators, updates on ongoing studies already endorsed by the DIPG Network, financial reports presented by the Administrative Office of DCOG, minutes discussions chaired by the Vice-Chair, and any other order of business deemed necessary for discussion by either the Executive Committee or any other DIPG Network members.

Even though the election of officers will be done electronically the general meetings also will serve for ratification of those positions.

DIPG Network Funding

Financial recourses will be needed to support the DIPG Network Administration as described above, as well as part of the meetings planned by the DIPG Network and the administration of the DIPG Network.

The Executive Committee will seek funding to support the DIPG Network activities. This funding may originate from third parties through national and international funding agencies and if possible charities, EU funding, participation in research, etc. All funds raised by or on behalf of the DIPG Network will be applied to further the mission and core values of the DIPG Network and for no other purpose. The applicable DIPG Network Administrator appointed by the Executive Committee will keep the accounting records and prepare the reports annually or at such other intervals as the Executive Committee may require. The DIPG Network accounts will be examined or audited at least once a year.

All contribution by the DIPG Network consists of providing access to highly qualified and dedicated investigational sites and investigators from its DIPG Network and making available data from the DIPG registry. DIPG Network shall not make available financial resources and is not considered a funding entity.

Changes

Members can propose changes to the Bylaws . Proposals for changes will be provided to the Executive Committee at least two months before the bi-annual meeting.

These Bylaws may be changed as follows:

- The Executive Committee will prepare any changes

- The proposed changes will be sent to the members and be added to the agenda of the DIPG Network meeting

- Changes will become effective if 2/3 of the Members represented at the DIPG Network meeting in accordance with the quorum, have voted for the change.

**Annex 1: Terms and conditions for submitting, reviewing and approving Proposals**

The Executive Committee will consider studies that make use of data from the DIPG Registry that comply with mission and core values of the DIPG Network. The committee will preferentially endorse protocols submitted prospectively (i.e., before any study activities are undertaken).

However, the Executive Committee may decide to retrospectively review a proposal if such is justified by exceptional circumstances (i.e., timelines for grant applications or funding). The Executive Committee will review investigator-initiated protocols. All study protocols must be developed in accordance with Good Clinical Research Practice Guidelines and in compliance with the regulations and ethical guidelines for the protection of human subjects.

In general the protocol format should include the following:

1. Cover letter addressed to the Executive Committee that details expectation from the review process
2. Name of Principal Investigator and affiliated institution.
3. Study aims, hypotheses, and rationale.
4. Background and significance.
5. Preliminary data.
6. Study population: inclusion and exclusion criteria with justification, protocol for intervention and standard treatment, measurements and methodology of data collection, masking and randomization (if applicable), timelines (including patient accrual plan), and statistical analysis (including sample size calculations and analysis of outcome measures).
7. Human subject protection: data and safety monitoring, safety-related stopping rules, interim analyses, and reporting of adverse events.
8. Plan for monitoring and compliance: sample consent form, IRB submission or equivalent, and study monitoring plan (including protocol deviations and violations and data quality).
9. Dissemination and data sharing plan.
10. Budget and costs breakdown

Application process

Any Researcher, as defined in the Regulatory Document, can submit a proposal for a DIPG study for consideration by sending an email to info@dipg.eu.

The Executive Committee will review the proposal and nominate two of its members and/or one member and one scientific advisor as determined by the Executive Committee, who are not principal investigators on the study, to serve as anonymous reviewers. Principal Investigators will formally present their protocols to the Executive Committee via teleconferencing and to the entire DIPG Network membership during one of the face-to-face meetings. This will allow for exposure and feedback of all DIPG Network members to the protocols and identification of potential participating sites.

Three possible outcomes are expected: two recommendations for endorsement, two conflicting recommendations, or two recommendations for rejection. If two recommendations for endorsement are received notification of the pending endorsement will be sent to all Executive Committee members who are offered one week to express their comments or concerns. If there is agreement, a letter that advises DIPG Network endorsement will be sent to the applicant. If two conflicting recommendations are received, the proposal is discussed during Executive Committee meeting to decide the course of action. Once a consensus is reached, a notification will be sent to the applicant. If both reviewers recommend that the application be rejected, the Chair of the Executive Committee will personally notify the applicant.

If appropriate, the Principal Investigator will have the opportunity to respond to reviewers’ comments and suggested amendments and to resubmit the proposal.

In addition, Principal Investigators may appeal in the event that endorsement is rejected. Appeals will be reviewed by the Executive Committee, and where appropriate by one of the Scientific Advisors that has not been involved in the initial review procedure.

Conditions of endorsement

The DIPG Network will endorse proposals for studies using data from the DIPG Registry or clinical DIPG studies based on members review, taking into account whether the study is in compliance with its mission and core values and in compliance with the DIPG Registry regulatory document. The Principal Investigators’ duties include becoming a member of the DIPG Network (if not already). All Principal Investigators shall remain as the liaison with the DIPG Network for the duration of the study, submit all potential conflicts of interest to the DIPG Network Administrator for review by the Executive Committee, and prepare and present a study progress report (including patient accrual rate, funding, and adverse events) during one of the DIPG Network face-to-face meetings. The Executive Committee reserves the right to withdraw study endorsement at any time, without any liability of the DIPG Network, should the study deviate from the mission and core values of the DIPG Network or unmanageable conflicts of interest arise or are discovered.

The study data remain the ownership of the Principal Investigator but it is expected that he/she presents a data sharing plan.

Continuous use

In the event a Member leaves the DIPG Network for whatever reason, the data already transferred to the DIPG Registry shall remain in the DIPG Registry and be used for the purposes of the DIPG Registry pursuant to the version of the Regulatory Document at the date the Member leaves the DIPG Network. No further data from the leaving Member shall be entered into the DIPG Registry after the discontinuation of the member in the DIPG Network.

Publication Policy

All publications that result from DIPG Network-endorsed studies must include its name. The preferred authorship should be as “Listed investigators and the SIOPE DIPG Network Investigators”. A list of the DIPG Network Investigators must be provided as an appendix at the end of each publication.
All publications and presentations stemming from DIPG Network-endorsed studies must be prospectively approved by the Executive Committee. The procedures for review and approval are similar to those described above under conditions of endorsement for proposed studies. Investigators must not refer to the DIPG Network in those studies not endorsed by it.

**ANNEX 2a MODEL SCIENTIFIC ADVISORY AGREEMENT**

**(directly with the Expert)**

This Agreement is made and entered into as of this day [***date***] (the “**Effective Date**”) by and between:

(1) **Dutch Childhood Oncology Group**, with its official address at Zinkwerf 5-7, 2544 EC, Den Haag,
 acting for and on behalf of the SIOPE DIPG Network;

On the one hand, and

(2) [***Scientific Advisor***] with its official address [address] (“**Expert**”),

On the other hand.

The Members and Expert are hereinafter individually (or collectively) referred to as a “**Party**” (or the “**Parties**”)

**WHEREAS**, the SIOPE Diffuse Intrinsic Pontine Glioma (DIPG) Network (hereinafter the “**Network**”) is a sub-committee of the high-grade glioma (HGG) working group of the Brain Tumour Group (BTG) of the International Society of Paediatric Oncology Europe (SIOPE) committed to supporting and fostering the mission of the Society;

**WHEREAS**, the Network is composed of paediatric oncologists, paediatric neurologists, radiotherapists, biologists, institutions and others motivated to carry out excellent clinical and biological research in the field of DIPG and collaborate with colleagues around the world;

**WHEREAS**, the Dutch Childhood Oncology Group (hereinafter “DCOG”) is the legal entity that on behalf of the members of the Network, including its Executive Committee, is authorized to enter into this Agreement;

**WHEREAS**, the Executive Committee (identified below) wishes to appoint the Expert to provide consultative and advisory services as further detailed in Article 2.

**NOW, THEREFORE**, in consideration of the premises and mutual covenants contained herein, the Parties hereto agree as follows:

**1. Definitions**

For the purposes of this Agreement the following terms shall have the following meaning:

1.1 “**Authorized Persons**” shall mean each Party’s independent contractors, agents,
 Affiliates, directors, officers, employees, members, consultants and legal or other
 professional advisers.

1.2 “**Confidential Information**” shall mean and collectively include any information, in
 tangible or non-tangible form, and/or physical items or materials, received from the Members or their Authorized Persons (the “**Disclosing Party**”) or generated in the performance of the Services, not generally available to the public, including but not limited to know-how, ideas, data, formulae, results of experimentation, specifications, inventions, techniques, research and development plans, business strategies, software and other technical or business information whether or not marked as confidential and whether obtained or disclosed in writing, orally, in connection with site visits or otherwise. Any such information received by Expert prior to the Effective Date shall also be deemed Confidential Information for purposes of this Agreement.

1.3 “**Executive Committee**” means the Executive Committee of the Network.

1.4 “**Expert**” means [name plus title of the advisor], who has specific expertise in the field of
 [add field] and who is affiliated to or an employee of [legal entity].

1.5 “**Results**” means any and all discoveries, developments, inventions (whether patentable or not), works of authorship, improvements, formulas, processes, compositions of
matter, formulations, methods of use or delivery, specifications, computer programs or models and related documentation, know-how or trade secrets conceived or first
reduced to practice, by Expert solely or in collaboration with others, in connection with the performance of this Agreement, or as a result of Confidential Information supplied to Expert, directly or indirectly, by a Disclosing Party.

1.6 “**Services**” means the services outlined in Article 2 hereof.

**2. Appointment**

2.1 The Expert hereby agrees to:

- offer honest and transparent advice to the Executive Committee in matters of development and implementation of research protocols, including ideas for innovative studies that could be executed through the Network.

- advise the Executive Committee on research proposals submitted to the Network.

2.2 Expert shall perform such Services with due care, skill and ability in accordance with
 the highest professional standards applicable to the Services.

2.3 Expert shall be available for the time needed and as agreed between the Parties and shall provide the Executive Committee with reports. Other than attendance at specific meetings, Expert may determine the hours or days he/she is to provide the Services and the location where he/she renders the Services.

2.4 Further details of any other Services provided by the Expert may be agreed in writing between the Parties.

**3. Compensation**

3.1 The Parties agree that the Expert shall not be compensated for the performance of his/her obligations under this Agreement.

3.2 DCOG on behalf of the Network will pay for modest travel expenses and hospitality, such as flights, train travel, accommodation and work-related meals that are pre-approved by the Executive Committee in writing.

3.3 Any payments made pursuant to the above, will be made within sixty (60) days to an account nominated by the Expert prior in writing upon receipt of a correct invoice (i) complying with applicable legal and tax requirements and (ii) containing the original receipts. Further details will be agreed between the Parties.

3.4 Expert shall be responsible for all other taxes payable on account of payments made hereunder.

3.5 Expert agrees that the Executive Committee may store, process and publish any payments made on behalf of the Network under this Agreement, if such disclosure is required by statutory or internal regulation or any binding code of conduct.

**4. Confidentiality**

4.1 Expert agrees to use the same level of care used with its own information of a similar nature to keep confidential the Confidential Information it receives from the Disclosing Party, which shall at all times be at least reasonable standard of care.

4.2 Expert agrees that it will neither disclose to any third party the Confidential Information it receives from the Disclosing Party nor use such Confidential Information for any purpose other than to accomplish the Purpose of this Agreement. Expert agrees that it will disclose the Disclosing Party’s Confidential Information only to Authorized Persons who (i) have a need to access such

information to accomplish the Purpose of this Agreement; and (ii) agree to be bound by the terms at least as restrictive as the terms of this Agreement. Expert agrees that the Disclosing Party’s Confidential Information shall not be used for the Expert’s benefit, or on behalf or for the benefit of its employer.

4.3 Expert’s obligations under Paragraphs 4.1 and 4.2 above shall not extend to all or any part of the Confidential Information:

a. that was in the public domain or publicly known or available prior to the date of the disclosure to the Expert; or

b. that was rightfully in the possession of the Expert prior to the disclosure to the Expert as it can be demonstrated by competent evidence; or

c. that becomes part of the public domain or publicly known or available by publication or otherwise, not due to any unauthorized act or omission on the part of the Expert; or

d. that is supplied to the Expert by a third party without binder of secrecy, provided, however, that, such third party has no obligation to the Disclosing Party to maintain such information in confidence; or

e. that is subsequently and independently developed by employees, independent contractors, agents of the Expert without reference to or reliance upon such Confidential Information as it can be demonstrated by competent evidence.

4.4 The Expert may disclose Confidential Information that is required to be disclosed by valid statute or governmental rule or regulation or by a competent court or administrative body, provided that the Expert limits disclosure to the extend requested by application of the relevant legal provision or order and provided that the Expert: (i) to the extent legally permissible and reasonably practicable, shall promptly notify the Disclosing Party to allow Disclosing Party to exercise any actions to prevent or further limit the disclosure; (ii) limits disclosure to the portion of the Disclosing Party’s Confidential Information which the Expert is legally required to disclose; and (iii) provides reasonable assistance to the Disclosing Party in obtaining an order or other remedy protecting the Confidential Information from the disclosure. Confidential Information that is disclosed pursuant to such required disclosure shall remain otherwise subject to the confidentiality and non-use provisions set forth herein.

4.5 Upon (i) completion of the Services and in the absence of any further written agreement between the Parties; or (ii) request by the Disclosing Party at any time, the Expert shall cease all use of the Disclosing Party’s Confidential Information and promptly return or destroy originals, copies, and summaries of documents, materials, and other tangible manifestations of Confidential Information in the possession or control of the Expert; provided, however, that one (1) copy of the Confidential Information may be retained by the Expert for the sole purpose of monitoring its ongoing obligations hereunder.

**5. Rights to Results**

5.1 Expert shall promptly disclose any Results to the Executive Committee and DCOG in writing. All rights, title and interest in any Results will be owned exclusively by the Network member(s) indicated by the Executive Committee, and Expert shall assign (or cause to be assigned) and does hereby assign fully to each of such Network member(s) all right, title and interest in and to any such Results, without payment of any additional compensation to the Expert. With regard to any copyrights, Expert consents to the right to reproduce, modify and use all copyrightable works designed or made by the Expert by each of the Network members.

**6. Liability**

6.1 The Expert shall be liable for and agrees to indemnify and hold harmless the other Party, the Network members, their employees for any loss, damage, claim, suit, cost or expense arising out of his/her gross negligent or intentional acts in the performance of the Services under the Agreement.

**7. Compliance**

7.1 Expert agrees to comply with all applicable laws and regulations in the performance of this Agreement.

7.2 Expert agrees that the Executive Committee may disclose to third parties and the general public information regarding the Services provided hereunder, including the name of Expert [and its staff] assigned to carry out the Services, the location and the value of any payment or benefit in kind that Expert receives in relation to this Agreement, such as fees, travel and accommodation or other expenses.

7.3 Expert represents and warrants that:

a. he/she has received all required approvals in connection with entering into this Agreement and performing the obligations under this Agreement. If required, the legal entity the Expert is affiliated to or which is the employer of the Expert may confirm that it has no objections to Expert entering into this Agreement, through an authorized representative’s signature at the place indicated below;

b. compliance with, and performance of, the terms of this Agreement do not and will not breach or conflict with (i) any other agreement or arrangement to which Expert is a party; or (ii) any statutory or internal regulations Expert is subject to; (iii) any agreement to keep in confidence proprietary information acquired in confidence or

in trust. Expert agrees it shall not enter into any such conflicting agreements during
 the term of this Agreement;

c. during performance of the Agreement, Expert will not disclose to the Network members, or induce Network members to use, any proprietary information belonging to a third party;

d. if there is any disclosure obligation to a third party (including, but not limited to, any healthcare institution, medical committee, or other medical or scientific organization with which Expert is affiliated) regarding the existence and nature of Expert’s relationship with the Network, including the amount of any compensation received in relation thereto, Expert has made and will make in the future, any such disclosures;

e. he/she has the qualifications and ability to perform the Services in a professional manner, without the advice, control or supervision of Executive Committee, any Network member or DCOG;

f. He/she is not prohibited by any applicable laws, regulations or ethical guidelines from furnishing Services;

g. if he/she is a member of a committee that sets formularies or develops clinical guidelines, he/she has to disclose the existence and nature of Expert’s relationship with the Network to such committee and he/she shall follow all procedures set forth by such committee related to managing any potential conflict of interest; and

h. if he/she, consistent with the terms of this Agreement, speaks publicly or publishes any article or letter about any matter related to Services or that otherwise relates to the Network, he/she will disclose the existence of his/her relationship with Network.

[*insert if US only*]

i. where Expert is an officer or employee of the United States government or is serving on a detail to the United States government, or is an officer or enlisted member of the uniformed services, Expert represents and warrants that he/she has obtained a written waiver from his or her ethics officer to enter into this Agreement, provide the services to the Members[, and receive expenses and/or compensation in connection

with the services provided hereunder]. Upon request, Expert shall provide DCOG
with a copy of such waiver;

j. Expert is not an Ineligible Person, as defined below, and agrees to recertify in writing to the Executive Committee at any time in the future and at the request of DCOG, that Expert is not an Ineligible Person. Expert further agrees (1) to disclose immediately to the Executive Committee any debarment, disqualification, exclusion, suspension, or other event that makes Expert an Ineligible Person, or any notice or threat thereof, or any facts and circumstances that may lead to Expert becoming an Ineligible Person; and ii) to promptly provide to DCOG all data requested by the Executive Committee for purposes of complying with any disclosure, reporting or compliance obligations under Federal and/or state laws relating to any reporting obligations for Ineligible Persons. If Expert becomes an Ineligible Person, this Agreement shall terminate, effective when Expert became an Ineligible Person, and all obligations of the Network are immediately terminated. The notification obligations of this subsection shall survive termination of this Agreement.

k. For purposes of this Agreement, an “Ineligible Person” is an individual or entity who: (i) is excluded, debarred, suspended, or otherwise ineligible to participate in the federal health care programs or in Federal procurement or non-procurement programs; (ii) has been convicted of a criminal offense that falls within the ambit of 42 U.S.C. § 1320a-7(a), but has not yet been excluded, debarred, suspended, or otherwise declared ineligible; or (iii) is debarred under the provisions of the Generic Drug Enforcement Act of 1992, 21 U.S.C. §335a(a) and (b), or disqualified as a clinical investigator under the provisions of 21 C.F.R. §312.70.

**8. Anti-Bribery and Anti-Corruption**

8.1 The Expert represents and warrants that he/she nor any of his/her personnel nor sub-contractors are officials, agents, representatives or employees of any government or political party or any international public organization where they may be in a position of official government authority able to use that position to help Members or any of their Affiliates to obtain or maintain business or obtain a business advantage. The Expert further represents and warrants that he/she has not and agrees that he/she or any of his/her personnel or sub-contractors shall not make any payment or any offer or promise for payment, either directly or indirectly, of money or other assets, to government or political party officials, officials of international organizations, candidates for public office, or representatives of other businesses or persons acting on behalf of any of the foregoing for the purpose of influencing decisions or actions or where such payment would constitute violation of any applicable anti-bribery/anti-corruption law provisions.

8.2 In the event the Expert is a government employee, he/she represents and warrants that he/she has not accepted nor been offered any payment of money or other assets for the purpose of influencing his/her decisions or actions to help Members obtain or maintain business or obtain a business advantage where such payment would constitute violation of any applicable anti-bribery/anti-corruption law provisions. The Expert further represents and warrants that he/she has not made and agrees that he/she shall not make any payment or any offer or promise for payment, either directly or indirectly, of money or other assets, to government or political party officials, officials of international organizations, candidates for public office, or representatives of other businesses or persons acting on behalf of any of the foregoing for the purpose of influencing decisions or actions or where such payment would constitute violation of any applicable anti-bribery/anti-corruption law provisions.

**9. Audit**

9.1 During the term of the Agreement and for three (3) years thereafter, the Executive Committee’s designated representative may, during business hours and upon reasonable advanced notice to the Expert inspect, copy and audit all books, data, records and work products of Expert that relate to the Agreement. The Expert will retain detailed and up to date books of account and records of all acts performed in connection with the Agreement for seven (7) years following the termination date of the Agreement.

**10. Personal Data Protection**

10.1 The Expert is informed and hereby agrees that DCOG collects and processes his/her personal data, and may transfer such personal data to the Executive Board and if applicable to Network members in order to allow the Network to manage the relationship with their experts. Where a Network member, including the Executive Committee is in receipt of the Expert’s personal data, such member will make every effort to protect the personal data from loss or unauthorised use or disclosure. The Expert is informed that he/she has the right to access and, where appropriate, to request the rectification and/or deletion of his/her personal data.

**11. General**

11.1 This Agreement shall be effective as of the Effective Date and will remain in full force and effect for a term of [term].

11.2. DCOG may immediately terminate this Agreement without cause by giving written notice to the Expert.

11.3 Neither this Agreement nor the disclosure by the Disclosing Party hereunder shall be deemed by implication or otherwise to vest in the Expert any right or license in, to or under (a) any Confidential Information so disclosed, or (b) any intellectual property rights of the Disclosing Party, including those covering or related to such Confidential Information. The disclosure of Confidential Information shall not result in any obligation to grant the Expert any rights therein.

11.4 The Parties are independent contractors. Nothing contained herein shall be construed as an employee/employer relationship between Expert and DCOG or any member of the Network. Expert agrees that its staff, employees and agents are not entitled to any benefits or rights associated with an employment relationship from any of the DCOG or members of the Network. No Party is the partner, joint venturer, or agent of another and no Party has authority to make any statement, representation, commitment, or action of any kind which purports to bind another (including the Network members) without such other's prior written authorization.

11.5 DCOG shall be entitled to assign or otherwise transfer, wholly or partly, the rights and obligations resulting from the Agreement to another Network member. The Expert undertakes to sign and to have any authorized Persons sign, at the request of DCOG, any document necessary to the legal and administrative regularization of such assignment or transfer. The Expert shall not be entitled to assign, transfer or subcontract any of the rights or obligations herein without the prior written consent of the other Party.

11.6. Expert acknowledges that any member of the Network shall have the right to enforce any breach of this Agreement by the Expert, to the extent such member of the Network’s rights are affected. The Executive Committee shall have the right, but not the obligation, to enforce the terms of this Agreement at the request of any of such Network member. Expert further acknowledges that each Network member shall be fully and solely liable for any damage or loss caused by a breach of this Agreement by such member and/or its Authorized Persons and, for the avoidance of doubt, DCOG and the Executive Committee will have no responsibility or liability, jointly or severally, in respect of another Network member’s or its Authorized Persons’ breaches of an agreement.

11.7 Notices or other communications required to be sent under this Agreement to:

(a) Expert shall be addressed to [add contact information]; and

(b) DCOG shall be addressed to [add contact information]; and

(c) Executive Committee shall be addressed to [add contact information].

11.8 This Agreement represents the entire understanding and agreement of the Parties and supersedes all prior communications, agreements and understandings relating to the subject matter hereof. The provisions of this Agreement may not be modified, amended, nor waived, except by a written instrument duly executed by the Parties. This Agreement shall is made subject to and shall be construed under the laws of The Netherlands. The courts of The Hague (The Netherlands) will have exclusive jurisdiction to deal with any dispute which has arisen or may arise out, of or in connection with, this Agreement, except any party may bring proceedings for an injunction in any jurisdiction

11.9 The Expert shall not make any kind of publication or communication (i) on the Results hereunder without the prior written consent of the Executive Committee.

11.10 If any provision of this Agreement or the application in any particular circumstance is held illegal, invalid or unenforceable, such illegality, invalidity or unenforceability shall not affect any other provision hereof. This Agreement will, in such circumstances, be deemed modified to the extent necessary to render enforceable the provisions hereof to the fullest extent permitted by law.

11.11 This Agreement may be executed in two or more counterparts, each of which shall be deemed an original, but all of which together shall constitute one and the same instrument.

*[signature page follows]*

IN WITNESS WHEREOF, the DCOG has caused this Agreement to be executed by its duly authorized representatives, as of the Effective Date, in its own name and on behalf and in the name of the Network:

**Dutch Childhood oncology Group [DCOG]**

**Name:______________________**

**Title: __________________________**

**Date: _________________________


Signature:**

IN WITNESS WHEREOF, Expert has executed this Agreement as of the Effective Date:

**[Expert]**

**Name:______________________**

**Title: _______________________**

**Date: _______________________**

**Signature:**

[AGREED AND ACKNOWLEDGED by **[Expert’s EMPLOYER/Legal Entity], when applicable**.

**Signature: __________________________**

**Date: __________________________**

**ANNEX 2b MODEL SCIENTIFIC ADVISORY AGREEMENT**

**(with legal entity and/in the presence of the Expert)**

This Agreement is made and entered into as of this day [***date***] (the “**Effective Date**”) by and between:

(1) **Dutch Childhood Oncology Group**, with its official address at […] Den Haag, acting for and on behalf of the SIOPE DIPG Network (“**DCOG**”);

On the one hand, and

(2) [Legal entity employing the expert], with its official address (“**Institution**”)

In the presence of

(3) [***Scientific Advisor***] with its official address [*address*] (“**Expert**”),

On the other hand.

The DCOG, Institution are hereinafter individually (or collectively) referred to as a “**Party**” (or the “**Parties**”)

**WHEREAS**, the SIOPE Diffuse Intrinsic Pontine Glioma (DIPG) Network (hereinafter the “**Network**”) is a sub-committee of the high-grade glioma (HGG) working group of the Brain Tumour Group (BTG) of the International Society of Paediatric Oncology Europe (SIOPE) committed to supporting and fostering the mission of the Society;

**WHEREAS**, the Network is composed of paediatric oncologists, paediatric neurologists, radiotherapists, biologists, institutions and others motivated to carry out excellent clinical and biological research in the field of DIPG and collaborate with colleagues around the world;

**WHEREAS**, the Dutch Childhood Oncology Group (hereinafter “DCOG”) is the legal entity that on behalf of the members of the Network, including its Executive Committee, is authorized to enter into this Agreement;

**WHEREAS**, the Executive Committee (identified below) wishes to appoint the Expert to provide consultative and advisory services as further detailed in Article 2 and the Institution approves the Expert to perform such services.

**NOW, THEREFORE**, in consideration of the premises and mutual covenants contained herein, the Parties hereto agree as follows:

**1. Definitions**

For the purposes of this Agreement the following terms shall have the following meaning:

1.1 “**Affiliate**” shall mean any legal entity that is under the direct or indirect control of a Party, under the same direct or indirect control as a Party, or is directly or indirectly controlling a Party, control taking any of the following forms: (a) the direct or indirect holding of more than at least 50% of the nominal value of the issued share capital in the legal entity concerned, or (b) of a majority of the voting rights of the shareholders or associates of that entity; or (c) the direct or indirect holding, in fact or in law, of decision-making powers in the legal entity concerned.

1.2 “**Authorized Persons**” shall mean each Party’s independent contractors, agents, Affiliates, directors, officers, employees, members, consultants and legal or other professional advisers.

1.3 “**Confidential Information**” shall mean and collectively include any information, in tangible or non-tangible form, and/or physical items or materials, received from the Members or their Authorized Persons (the “Disclosing Party”) or generated in the performance of the Services, not generally available to the public, including but not limited to know-how, ideas, data, formulae, results of experimentation, specifications, inventions, techniques, research and development plans, business strategies, software and other technical or business information whether or not marked as confidential and whether obtained or disclosed in writing, orally, in connection with site visits or otherwise. Any such information received by Expert prior to the Effective Date shall also be deemed Confidential Information for purposes of this Agreement.

1.4 “**Executive Committee**” means the Executive Committee of the Network.

1.5 “**Expert**” means [name plus title of the advisor], who has specific expertise in the field of [add field] and who is affiliated to or an employee of the Institution.

1.6 “**Results**” means any and all discoveries, developments, inventions (whether patentable or not), works of authorship, improvements, formulas, processes, compositions of matter, formulations, methods of use or delivery, specifications, computer programs or models and related documentation, know-how or trade secrets conceived or first reduced to practice, by Expert solely or in collaboration with others, in connection with the performance of this Agreement, or as a result of Confidential Information supplied to Expert, directly or indirectly, by a Disclosing Party.

1.7 “**Services**” means the services outlined in Article 2 hereof.

**2. Appointment**

2.1 The Expert hereby agrees to:

- offer honest and transparent advice to the Executive Committee in matters of development and implementation of research protocols, including ideas for innovative studies that could be executed through the Network.

- advise the Executive Committee on research proposals submitted to the Network.

2.2 Expert shall perform such Services with due care, skill and ability in accordance with the highest professional standards applicable to the Services.

2.3 Expert shall be available for the time needed and as agreed between the Parties and shall provide the Executive Committee with reports. Other than attendance at specific meetings, Expert may determine the hours or days he/she is to provide the Services and the location where he/she renders the Services.

2.4 Further details of any other Services provided by the Expert may be agreed in writing between the Parties.

**3. Compensation**

3.1 The Parties agree that the Expert shall not be compensated for the performance of his/her obligations under this Agreement.

3.2 DCOG on behalf of the Network will pay for modest travel expenses and hospitality, such as flights, train travel, accommodation and work-related meals that are pre-approved by the Executive Committee in writing.

3.3 Any payments made pursuant to the above, will be made within sixty (60) days to an account nominated by the Institution prior in writing upon receipt of a correct invoice (i) complying with applicable legal and tax requirements and (ii) containing the original receipts. Further details will be agreed between the Parties.

3.4 Institution shall be responsible for all other taxes payable on account of payments made hereunder.

3.5 Institution and Expert agree that the Executive Committee may store, process and publish any payments made on behalf of the Network under this Agreement, if such disclosure is required by statutory or internal regulation or any binding code of conduct.

**4. Confidentiality**

4.1 Institution and Expert agree to use the same level of care used with its own information of a similar nature to keep confidential the Confidential Information either of them receives from the Disclosing Party, which shall at all times be at least reasonable standard of care.

4.2 Institution and Expert agree that the will neither disclose to any third party the Confidential Information it receives from the Disclosing Party nor use such Confidential Information for any purpose other than to accomplish the Purpose of this Agreement. Institution and Expert agree that they will disclose the Disclosing Party’s Confidential Information only to Authorized Persons who (i) have a need to access such information to accomplish the Purpose of this Agreement; and (ii) agree to be bound by the terms at least as restrictive as the terms of this Agreement. Institution and Expert agree that the Disclosing Party’s Confidential Information shall not be used for the Institution’s and/or Expert’s benefit.

4.3 Institution’s and Expert’s obligations under Paragraphs 4.1 and 4.2 above shall not extend to all or any part of the Confidential Information:

a. that was in the public domain or publicly known or available prior to the date of the disclosure to the Institution and/or Expert; or

b. that was rightfully in the possession of the Institution (or its Affiliates) and/or Expert prior to the disclosure to the Institution and/or Expert as it can be demonstrated by competent evidence; or

c. that becomes part of the public domain or publicly known or available by publication or otherwise, not due to any unauthorized act or omission on the part of the Institution and/or Expert; or

d. that is supplied to the Institution (or its Affiliates) and/or Expert by a third party without binder of secrecy, provided, however, that, such third party has no obligation to the Disclosing Party to maintain such information in confidence; or

e. that is subsequently and independently developed by the Expert and/or the employees, independent contractors, agents, or Affiliates of the institution without reference to or reliance upon such Confidential Information as it can be demonstrated by competent evidence.

4.4 The Institution and/or Expert may disclose Confidential Information that is required to be disclosed by valid statute or governmental rule or regulation or by a competent court or administrative body, provided that the Institution/Expert limit disclosure to the extend requested by application of the relevant legal provision or order and provided that the Institution and/or Expert: (i) to the extent legally permissible and reasonably practicable, shall promptly notify the Disclosing Party to allow Disclosing Party to exercise any actions

to prevent or further limit the disclosure; (ii) limits disclosure to the portion of the Disclosing Party’s Confidential Information which the Institution or Expert are legally required to disclose; and (iii) provides reasonable assistance to the Disclosing Party in obtaining an order or other remedy protecting the Confidential Information from the disclosure. Confidential Information that is disclosed pursuant to such required disclosure shall remain otherwise subject to the confidentiality and non-use provisions set forth herein.

4.5 Upon (i) completion of the Services and in the absence of any further written agreement between the Parties; or (ii) request by the Disclosing Party at any time, the Institution and the Expert shall cease all use of the Disclosing Party’s Confidential Information and promptly return or destroy originals, copies, and summaries of documents, materials, and other tangible manifestations of Confidential Information in the possession or control of the Institution and/or Expert; provided, however, that one (1) copy of the Confidential Information may be retained by the Institution for the sole purpose of monitoring its and Expert’s ongoing obligations hereunder.

**5. Rights to Results**

5.1 Institution and Expert shall promptly disclose any Results to the Executive Committee and DCOG in writing. All rights, title and interest in any Results will be owned exclusively by the Network member(s) indicated by the Executive Committee, and Institution shall assign and to the extent applicable shall ensure that the Expert shall assign and does hereby assign fully to each of such Network member(s) all right, title and interest in and to any such Results, without payment of any additional compensation to the Institution and/or Expert. With regard to any copyrights, Institution and Expert consent to the right to reproduce, modify and use all copyrightable works designed or made by the Expert by each of the Network members.

**6. Liability**

6.1 The Institution shall be liable for and agrees to indemnify and hold harmless the other Party, the Network members, their employees for any loss, damage, claim, suit, cost or expense arising out of his/her gross negligent or intentional acts in the performance of the Services by the Expert under the Agreement.

**7. Compliance**

7.1 Institution and Expert agree to comply with all applicable laws and regulations in the performance of this Agreement.

7.2 Institution and Expert agree that the Executive Committee may disclose to third parties and the general public information regarding the Services provided hereunder, including the name of Expert [and its staff] assigned to carry out the Services, the location and the value of any payment or benefit in kind that Expert receives in relation to this Agreement, such as fees, travel and accommodation or other expenses.

7.3 the Institution represents and warrants that:

a. the Expert has received all required approvals in connection with entering into this Agreement and performing the obligations under this Agreement;

b. compliance with, and performance of, the terms of this Agreement do not and will not breach or conflict with (i) any other agreement or arrangement to which Expert is a party; or (ii) any statutory or internal regulations Expert is subject to; (iii) any agreement to keep in confidence proprietary information acquired in confidence or in trust. Expert agrees it shall not enter into any such conflicting agreements during the term of this Agreement;

c. during performance of the Agreement, Expert will not disclose to the Network members, or induce Network members to use, any proprietary information belonging to a third party;

d. if there is any disclosure obligation to a third party (including, but not limited to, any healthcare institution, medical committee, or other medical or scientific organization with which Expert is affiliated) regarding the existence and nature of Expert’s relationship with the Network, including the amount of any compensation received in relation thereto, Expert has made and will make in the future, any such disclosures;

e. Expert has the qualifications and ability to perform the Services in a professional manner, without the advice, control or supervision of Executive Committee, any Network member or DCOG;

f. Expert is not prohibited by any applicable laws, regulations or ethical guidelines from furnishing Services;

g. if the Expert is a member of a committee that sets formularies or develops clinical guidelines, he/she has to disclose the existence and nature of Expert’s relationship with the Network to such committee and he/she shall follow all procedures set forth by such committee related to managing any potential conflict of interest; and

h. if the Expert, consistent with the terms of this Agreement, speaks publicly or publishes any article or letter about any matter related to Services or that otherwise relates to the Network, he/she will disclose the existence of his/her relationship with Network.

[*insert if US only*]

i. where Expert is an officer or employee of the United States government or is serving on a detail to the United States government, or is an officer or enlisted member of the uniformed services, Institution shall ensure that the Expert represents and warrants that he/she has obtained a written waiver from his or her ethics officer to enter into this Agreement, provide the services to the Members[, and receive expenses and/or compensation in connection with the services provided hereunder]. Upon request, Expert shall provide DCOG with a copy of such waiver;

j. Expert is not an Ineligible Person, as defined below, and agrees to recertify in writing to the Executive Committee at any time in the future and at the request of DCOG, that Expert is not an Ineligible Person. Expert further agrees (1) to disclose immediately to the Executive Committee any debarment, disqualification, exclusion, suspension, or other event that makes Expert an Ineligible Person, or any notice or threat thereof, or any facts and circumstances that may lead to Expert becoming an Ineligible Person; and ii) to promptly provide to DCOG all data requested by the Executive Committee for purposes of complying with any disclosure, reporting or compliance obligations under Federal and/or state laws relating to any reporting obligations for Ineligible Persons. If Expert becomes an Ineligible Person, this Agreement shall terminate, effective when Expert became an Ineligible Person, and all obligations of the Network are immediately terminated. The notification obligations of this subsection shall survive termination of this Agreement.

For purposes of this Agreement, an “Ineligible Person” is an individual or entity who: (i) is excluded, debarred, suspended, or otherwise ineligible to participate in the federal health care programs or in Federal procurement or non-procurement programs; (ii) has been convicted of a criminal offense that falls within the ambit of 42 U.S.C. § 1320a-7(a), but has not yet been excluded, debarred, suspended, or otherwise declared ineligible; or (iii) is debarred under the provisions of the Generic

Drug Enforcement Act of 1992, 21 U.S.C. §335a(a) and (b), or disqualified as a clinical investigator under the provisions of 21 C.F.R. §312.70.

**8. Anti-Bribery and Anti-Corruption**

8.1 The Institution ensures that through representation and warranties, the Expert, any of the Institution’s personnel or sub-contractors are officials, agents, representatives or employees of any government or political party or any international public organization where they may be in a position of official government authority able to use that position to help Members or any of their Affiliates to obtain or maintain business or obtain a business advantage. The Expert further represents and warrants that he/she has not and agrees that he/she or any of his/her personnel or sub-contractors shall not make any payment or any offer or promise for payment, either directly or indirectly, of money or other assets, to government or political party officials, officials of international organizations, candidates for public office, or representatives of other businesses or persons acting on behalf of any of the foregoing for the purpose of influencing decisions or actions or where such payment would constitute violation of any applicable anti-bribery/anti-corruption law provisions.

8.2 In the event the Expert is a government employee, the Institution ensures through
 representation and warranties that the Expert

8.2.1 has not accepted nor been offered any payment of money or other assets for the
 purpose of influencing his/her decisions or actions to help Members obtain or
 maintain business or obtain a business advantage where such payment would
 constitute violation of any applicable anti-bribery/anti-corruption law provisions,
 and

8.2.2 has not made and agrees that he/she shall not make any payment or any offer or
 promise for payment, either directly or indirectly, of money or other assets, to
 government or political party officials, officials of international organizations,
 candidates for public office, or representatives of other businesses or persons
 acting on behalf of any of the foregoing for the purpose of influencing decisions
 or actions or where such payment would constitute violation of any applicable
 anti-bribery/anti-corruption law provisions.

**9. Audit**

9.1 During the term of the Agreement and for three (3) years thereafter, the Executive Committee’s designated representative may, during business hours and upon reasonable advanced notice to the Institution and Expert inspect, copy and audit all books, data, records and work products of Expert that relate to the Agreement. The

Institution and/or Expert will retain detailed and up to date books of account and records of all acts performed in connection with the Agreement for seven (7) years following the termination date of the Agreement.

**10. Personal Data Protection**

10.1 The Institution and the Expert are informed and hereby agree that DCOG collects and processes personal data relating to the Institution and the Expert, and may transfer such personal data to the Executive Board and if applicable to Network members in order to allow the Network to manage the relationship with their experts. Where a Network member, including the Executive Committee is in receipt of the Institution’s and the Expert’s personal data, such member will make every effort to protect the personal data from loss or unauthorised use or disclosure. The Institution and the Expert are informed that he/she has the right to access and, where appropriate, to request the rectification and/or deletion of the/his/her personal data.

**11. General**

11.1 This Agreement shall be effective as of the Effective Date and will remain in full force and effect for a term of [term].

11.2. DCOG may immediately terminate this Agreement without cause by giving written notice to the Institution and Expert.

11.3 Neither this Agreement nor the disclosure by the Disclosing Party hereunder shall be deemed by implication or otherwise to vest in the Expert any right or license in, to or under (a) any Confidential Information so disclosed, or (b) any intellectual property rights of the Disclosing Party, including those covering or related to such Confidential Information. The disclosure of Confidential Information shall not result in any obligation to grant the Expert any rights therein.

11.4 The Parties are independent contractors. Nothing contained herein shall be construed as an employee/employer relationship between Expert and DCOG or any member of the Network. Expert agrees that its staff, employees and agents are not entitled to any benefits or rights associated with an employment relationship from any of the DCOG or members of the Network. No Party is the partner, joint venturer, or agent of another and no Party has authority to make any statement, representation, commitment, or action of any kind which purports to bind another (including the Network members) without such other's prior written authorization.

11.5 DCOG shall be entitled to assign or otherwise transfer, wholly or partly, the rights and obligations resulting from the Agreement to another Network member. The Institution and to the extent applicable, the Expert undertake to sign and to have any authorized Persons sign, at the request of DCOG, any document necessary to the legal and administrative regularization of such assignment or transfer. The Institution nor the Expert shall be entitled to assign, transfer or subcontract any of the rights or obligations herein without the prior written consent of the other Party.

11.6. Institution and Expert acknowledges that any member of the Network shall have the right to enforce any breach of this Agreement by the Institution and/or Expert, to the extent such member of the Network’s rights are affected. The Executive Committee shall have the right, but not the obligation, to enforce the terms of this Agreement at the request of any of such Network member. Expert further acknowledges that each Network member shall be fully and solely liable for any damage or loss caused by a breach of this Agreement by such member and/or its Authorized Persons and, for the avoidance of doubt, DCOG and the Executive Committee will have no responsibility or liability, jointly or severally, in respect of another Network member’s or its Authorized Persons’ breaches of an agreement.

11.7 Notices or other communications required to be sent under this Agreement to:

(a) Institution shall be addressed to [add contact information; and
(b) Expert shall be addressed to [add contact information]; and
(c) DCOG shall be addressed to [add contact information]; and
(d) Executive Committee shall be addressed to [add contact information].

11.8 This Agreement represents the entire understanding and agreement of the Parties and supersedes all prior communications, agreements and understandings relating to the subject matter hereof. The provisions of this Agreement may not be modified, amended, nor waived, except by a written instrument duly executed by the Parties. This Agreement shall is made subject to and shall be construed under the laws of The Netherlands. The courts of The Hague (The Netherlands) will have exclusive jurisdiction to deal with any dispute which has arisen or may arise out, of or in connection with, this Agreement, except any party may bring proceedings for an injunction in any jurisdiction

11.9 The Institution and Expert shall not make any kind of publication or communication (i) on the Results hereunder without the prior written consent of the Executive Committee.

11.10 If any provision of this Agreement or the application in any particular circumstance is held illegal, invalid or unenforceable, such illegality, invalidity or unenforceability shall not affect any other provision hereof. This Agreement will, in such circumstances, be deemed modified to the extent necessary to render enforceable the provisions hereof to the fullest extent permitted by law.

11.11 This Agreement may be executed in two or more counterparts, each of which shall be deemed an original, but all of which together shall constitute one and the same instrument.

*[signature page follows]*

IN WITNESS WHEREOF, the DCOG has caused this Agreement to be executed by its duly authorized representatives, as of the Effective Date, in its own name and on behalf and in the name of the Network:

**Dutch Childhood oncology Group [DCOG]**

**Name:______________________**

**Title: __________________________**

**Date: _________________________**

**Signature:**

**[Institution]**

**Name:______________________**

**Title: __________________________**

**Date: _________________________**

**Signature:**

IN WITNESS WHEREOF, Expert has executed this Agreement as of the Effective Date:

**[Expert]**

**Name:______________________**

**Title: _______________________**

**Date: _______________________**

**Signature:**

AGREED AND ACKNOWLEDGED by **Expert**

**Signature: __________________________**

**Date: __________________________**
