# Supplementary material for: Development of the SIOPE DIPG network, registry and imaging repository: a collaborative effort to optimize research into a rare and lethal disease
Source: J Neurooncol. 2017 Jan 21;132(2):255–66. doi: 10.1007/s11060-016-2363-y (PMC5378734; doi:10.1007/s11060-016-2363-y)
Supplement: Supplementary file 3 — Supplementary material 3 (DOCX 154 KB) [file 11060_2016_2363_MOESM3_ESM.docx]

**Development of the SIOPE DIPG Network, Registry and Imaging Repository:**

**A collaborative effort to optimize research into a rare and lethal disease.**

Journal of Neuro-Oncology

*Sophie E.M. Veldhuijzen van Zanten, Joshua Baugh and Brooklyn Chaney, Dennis De Jongh, Esther Sanchez Aliaga, Frederik Barkhof, Johan Noltes, Ruben De Wolf, Jet Van Dijk, Antonio Cannarozzo, Carin M. Damen-Korbijn, Jan A. Lieverst, Niclas Colditz, Marion Hoffmann, Monika Warmuth-Metz, Brigitte Bison, David T.W. Jones, Dominik Sturm, Gerrit H. Gielen, Chris Jones, Esther Hulleman, Raphael Calmon, David Castel, Pascale Varlet, Géraldine Giraud, Irene Slavc, Stefaan Van Gool, Sandra Jacobs, Filip Jadrijevic-Cvrlje, David Sumerauer, Karsten Nysom, Virve Pentikainen, Sanna-Maria Kivivuori, Pierre Leblond, Natasha Entz-Werle, Andre O. von Bueren, Antonis Kattamis, Darren Hargrave, Péter Hauser, Miklos Garami, Halldora Kristin Thorarinsdottir, Jane Pears, Lorenza Gandola, Giedre Rutkauskiene, Geert O. Janssens, Ingrid K. Torsvik, Marta Perek-Polnik, Maria João Gil-da-Costa, Olga Zheludkova, Liudmila Shats, Ladislav Deak, Lidija Kitanovski, Ofelia Cruz, Andres Morales La Madrid, Stefan Holm, Nicolas Gerber, Rejin Kebudi, Richard Grundy, Enrique Lopez-Aguilar, Marta Zapata-Tarres, John Emmerik, Tim Hayden, Simon Bailey, Veronica Biassoni, Maura Massimino, Jacques Grill, William P. Vandertop, Gertjan J.L. Kaspers, Maryam Fouladi, Christof M. Kramm, Dannis G. van Vuurden on behalf of the members of the SIOPE DIPG Network.*

**Corresponsing author: Sophie E.M. Veldhuijzen van Zanten, VU University Medical Center Department of Pediatrics, Division of Oncology-Hematology, s.veldhuijzen@vumc.nl**

**
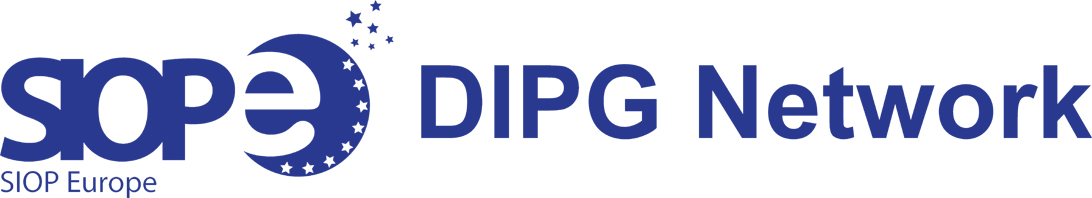
**

**DIPG Registry**

**and**

**Imaging Repository**

**Regulatory Document**

Contents

[**Introduction and Scope** 3](#_Toc448505012)

[**1.** **Definitions** 4](#_Toc448505013)

[**2.** **Data collection and transfer to the DIPG Registry** 5](#_Toc448505014)

[**3.** **Management** 5](#_Toc448505015)

[**4.** **Protection of privacy** 6](#_Toc448505016)

[**5.** **Conditions for Access** 7](#_Toc448505017)

[**6.** **Authorship** 8](#_Toc448505018)

[**7.** **Ownership and Intellectual property rights** 8](#_Toc448505019)

[**8.** **Accountability** 8](#_Toc448505020)

[**9.** **Donor complaints procedure** 8](#_Toc448505021)

[**10. About this document** 8](#_Toc448505022)

**APPENDIX A: MODEL CONFIDENTIALY AGREEMENT …………………………………………………………………………………………10

APPENDIX B: CERTIFICATE OF AUTHORITY…………………………………………………………………………………………………………13**

**Introduction and Scope**

Diffuse intrinsic pontine glioma (DIPG) is a very aggressive type of brain tumor, mostly occurring in children, for which currently no curative treatment exists. DIPG represent 10% of all pediatric central nervous system tumors. In most cases, DIPG are histological WHO grade II or III astrocytomas or glioblastoma multiforme (WHO grade IV) showing an extremely poor prognosis, with a 2 year overall survival of less than 10%. The last 20 years several therapies have been applied, but prognosis is unchanged. Radiotherapy is the only proven, albeit palliative, therapy to accomplish clinical and radiological responses. The median overall survival of patients with diffuse intrinsic pontine gliomas (DIPG) is nine months.

To improve prognosis, more knowledge about this group of tumors is necessary. It is important to review patients treated in the past and to learn from successful and less successful strategies. Information regarding epidemiology of DIPG is scant. The understanding of the incidence of these tumors is necessary in planning future strategies and protocol development. Furthermore, identifying clinical features that may be associated with prognosis would be important in future clinical trials. In addition, the international DIPG Registry being setup for this specific project will be of importance to be able to perform joint international trials in DIPG. These joint trials are necessary to progress with this devastating but relatively rare disease.

The International DIPG Registry (bringing together comprehensive data from multiple clinical trials and national registries) is established for the purpose of academic research. The Registry allows a better understanding of this rare disease and to define prognostic subgroups of DIPG patients. Results from clinical studies conducted in different countries are difficult to compare because of the lack of proper international agreements on diagnostic criteria and on central review. By using international centralized data collection with the possibility of central review, these results can be interpreted in the context of the SIOPE DIPG Network, hereinafter referred to as “DIPG Network”. International data centralization is essential in view of international studies in the future.

Clinical data will be submitted to the SIOPE DIPG Registry, hereinafter referred to as “DIPG Registry” by individual DIPG Network Members or each country’s national coordinator of the DIPG Network via web-based CRF forms to the DIPG Registry, facilitated by the Dutch Childhood Oncology Group (DCOG/SKION), hereinafter referred to as “DCOG”.

No personal identifiers are included in these CRFs. A unique DIPG Registry number is assigned to each case. Per country, a separate list, kept under a special password, connects the DIPG Registry number with the personal identifiers. Access to this list is restricted to each country’s national coordinator.

Neuroimaging data will be submitted to the DIPG Imaging Repository by the individual DIPG Network Members or national coordinators via a secure FTP-server or on CDs. After de-identification / pseudonymization, either in the referring center or at the time of receipt, these images are then uploaded into the SIOPE DIPG Imaging Repository, as part of the DIPG Registry using a Registry number from the DIPG Registry. Neuroradiologists as members of the DIPG Network or external neuroradiologists, on a central neuroradiology panel, will be provided access to view images on this system. An international central neuroimaging review panel will be assembled to conduct imaging review of submitted cases. If these neuroradiologists are members of the DIPG Network, they adhere to the DIPG Network Bylaws. Neuroradiologists outside the Network need to sign the confidentiality agreement, as shown in Appendix A.

This DIPG Registry and Imaging Repository Regulatory Document has been created taking into account the EU and different DIPG Network members’ national laws.

**1.** **Definitions**

1) In this Regulatory Document the following terms have the meanings ascribed to them below:

1. Access: Access to the Registry in accordance with Section 5.
2. Coded: processed i) by or on behalf of the Member making available Data to the DIPG Registry and ii) by or on behalf of the DIPG Network making available a Dataset to a Researcher, through reliable and safe information and communication technologies, in such manner that the party receiving Data cannot, without disproportional efforts, identify the Donors involved.
3. Data: The information collected from Donors that is transferred to and stored in the DIPG Registry in Coded form.
4. Dataset: the Data from the DIPG Registry made available for the purpose of a Project.
5. Donor: Any individual who’s Data are transferred to the DIPG Registry in compliance with the terms and conditions of this Regulation.
6. Dutch Childhood Oncology Group (DCOG): Dutch foundation and National Paediatric Haematology-Oncology Society, in which all Dutch paediatricians and other professionals specialized in research and treatment of children with cancer (paediatric oncology) are organized. DCOG will have tasks related to the DIPG Registry as outlined under 3.3 and 3.4
7. Executive Committee: The board that in accordance with the DIPG Network Bylaws has the authority to take decisions with respect to:

- Access for a Project; and

- The management of the DIPG Registry.

1. Findings: Results, data and information, whether or not they can be protected, which are generated as a result of a Project.
2. Funds: the amounts raised by the DIPG Network for the development and maintenance of the DIPG Registry.
3. Member: Each member of the DIPG Network.
4. National Coordinator: A Member coordinating the DIPG Network in the country of residence.
5. Project: Any scientific research based on an authorized project proposal involving Data.
6. Project Proposal: The document describing the scope, purposes and methodology of the Project.
7. Researcher: A person, institution, or organisation that undertakes Research in accordance with the conditions of this Regulation.
8. Terms and Conditions: the DIPG Network Terms and Conditions for submitting, reviewing and approving Proposals.

**2. Data collection and transfer to the DIPG Registry**

1. Members shall transfer into the DIPG Registry free of charge, Coded Data of patients that consented and are eligible to participate in the DIPG Registry. The Data in the DIPG Registry shall be used for further research purposes.
2. Each Member shall remain the owner of the Data it transfers into the Registry. Transfer of Data into the DIPG Registry shall not restrict any use of such Data by the Member that have contributed such Data.
3. It is the responsibility and liability of each Member transferring Data into the DIPG Registry to ensure such transfer is in compliance with their national law, including but not limited to privacy laws and that such Data can be used for the purposes of the DIPG Registry. As a consequence, if required by or if subject to a Member’s national law or Institution policies, the terms and conditions of the DIPG Registry shall be submitted to a (ethical) review board, together with the informed consent forms and other relevant documents related to the DIPG Registry as determined by such review board.
4. Data shall only be made available from the DIPG Registry on behalf of the DIPG Network for Projects after approval has been obtained for the Proposal in accordance with Section 5 hereof.

**3. Management**

1. The composition and duties of the DIPG Network Executive Committee are outlined in the DIPG Network Bylaws. The Executive Committee will be responsible for overall management of the DIPG Registry on behalf of the Members. The Executive Committee shall be responsible for monitoring the proper financial management and hosting of the DIPG Registry. More specifically, the Executive Committee shall be responsible for:
   1. Approving to guidelines, specifications and Standard Operating Procedures;
   2. Publication of guidelines and SOPs on a DIPG Registry website;
   3. Informing the Members on the progress of the DIPG Registry;
   4. Reviewing and approving Project Proposals in accordance with Section 5 and the Terms and Conditions.
2. The financial management and hosting of the DIPG Registry will be carried out by the Dutch Childhood Oncology Group (DCOG), a National Paediatric Haematology-Oncology Society (NaPHOS) member of SIOPE. DCOG is mandated by the Executive Committee of the DIPG Network to act as a legal entity on its behalf in matters concerning the DIPG Registry, by a letter of mandate, as shown in Appendix B.
3. The responsibilities of DCOG include:
   1. conclude on behalf of the DIPG Registry written agreements with regard to Funds received from third parties for the DIPG Registry
   2. receive, administer and allocate all Funds for the DIPG Registry in accordance with the reasonable instructions of the Executive Committee.
   3. conclude written agreements with third parties providing services to the DIPG Registry, including but not limited to such agreement with the DIPG Imaging Repository
   4. conclude on behalf of the DIPG Registry written agreements with scientific advisors based on the model agreement attached to the Bylaws as Annex 2.
   5. invoice third parties on behalf of and as instructed by Executive Committee where appropriate
   6. pay invoices received from DIPG Network coordinators, third party service providers and other third parties in relation with any agreements executed by or for the DIPG Network in relation to the DIPG Registry as approved by the Executive Committee;
   7. DCOG shall ensure transparent book-keeping in relation to the DIPG Registry’s financial administration.
   8. any responsibilities agreed between the Executive Committee and DCOG, pursuant to the decision making procedures of the DIPG Network.
4. Maintenance of the Registry shall be paid from Funds. All projected expenditures related to the Registry paid from the Funds shall require the prior approval of the Executive Committee. However DCOG shall only pay invoices from the DIPG Registry funds to the extent funds are available to cover such expenses. In no event shall DCOG be responsible nor liable for paying any invoices or costs if these are not covered by the DIPG Registry funding.
5. DCOG shall furthermore be responsible for hosting the DIPG Registry in accordance with applicable privacy law and the instructions of the Executive Committee. The DIPG Registry shall be kept in a secured area. DCOG shall ensure that Data stored in the DIPG Registry is adequately protected and that sufficient technical and other measures have been put in place to prevent unauthorised access to the DIPG Registry and to prevent the loss, theft and unauthorised access and use of the Data.
6. DCOG shall not be responsible or liable for any failure or a Member to comply with its national law and/or institutional policies with regard to transferring Data into the DIPG Registry. DCOG shall not be liable for any breach of contract by a Member of a contract concluded by DCOG pursuant to article 3.3 a, c and d.
7. DCOG shall provide a copy of each contract concluded under article 3.3. a, c and d above to the Executive Committee, who will be responsible for providing such document to the Members.

**4. Protection of privacy**

1. Members shall ensure that the privacy of the Donors and the confidentiality of Data are protected in accordance with the statutory requirements applicable in their own country and the policies of the institution of which the Donor is a patient.
2. Members shall transfer Data into the DIPG Registry in Coded form only, in accordance with the guidelines and instructions of the Executive Committee.
3. Prior to making available the Data for a Project, the Dataset to be transferred shall be given a new Code.
4. The Executive Committee shall inform Members about the method of coding and the location of the DIPG Registry (and any changes to the location).

**5. Conditions for Access**

1. Researchers shall be bound by this Regulation. The applicable conditions must be made known to the Researcher at least prior to the review of the Project Proposal.
2. Researchers requesting Data shall submit their Project Proposal to the address shown on the DIPG Network website and in accordance with the Terms and Conditions.
3. Making available Data shall be conditional to obtained approval from the Executive Committee and to the extent applicable to the permits and licenses required by the Researcher’s national law.
4. If the Executive Committee approves a Project Proposal, the Researcher will receive a ‘Letter of Approval’ stipulating the general terms and condition with regard to receipt and use of the Data, a signed copy of which must be returned to the Executive Committee prior to making the Data available.
5. The Executive Committee in its reasonable decision, following the advice of the advisors (mentioned in the Terms and Conditions) may set additional conditions to a specific Project. Such additional conditions will be communicated to the Researcher during the reviewing process and will be added to the Letter of Approval if the Project Proposal is approved.
6. Access to Data shall at least be conditional to the following:
7. The Researcher shall be responsible for obtaining the permits and approvals necessary in its own country and the policies of its institution.
8. The Researcher shall use the Data for the approved Project only. In case of deviations or changes in the project the Executive Committee shall have the right to terminate Access without any liability at its sole discretion
9. The Researchers shall bear sole responsibility for the handling and use of the Data in accordance with applicable law and legislation
10. The Researcher shall not duplicate the Data or have them duplicated
11. The Researcher shall not disclose or provide access to the Data to any third party without the prior written consent of Executive Committee.
12. The Researcher shall report the progress of the Project and Findings in a frequency as outlined in the Letter of Approval
13. Findings must be shared with the research community at large and therefore be scientifically published in accordance with the Terms and Conditions.
14. Researchers, conditional to the above, shall have access to the data free of charge.
15. Upon receipt of the Letter of Approval signed by the Researcher [and the fee mentioned in the Letter of Approval], the Data necessary to perform the Project will be selected from the DIPG Registry and sent to the Researcher.

1. The Findings shall be owned by the Researcher. The DIPG Network shall have a non-exclusive right to use the Data for the purpose of further research in the field of DIPG and development of professional and/or medical standards.

**6. Authorship**

The publication of Findings generated with data from the DIPG Registry needs to comply with rules concerning authorship, as defined by the International Committee of Medical Journal Editors (ICMJE). To qualify as author, at least the following criteria apply to the contribution of the investigator in respect to the intended publication:

- Substantial contributions to the conception or design of the work; or the acquisition, analysis, or interpretation of data for the work; AND
- Drafting the work or revising it critically for important intellectual content; AND
- Final approval of the version to be published; AND
- Agreement to be accountable for all aspects of the work in ensuring that questions related to the accuracy or integrity of any part of the work are appropriately investigated and resolved.

**7. Ownership and Intellectual property rights**

Without prejudice to the ownership of the Data by the Members and ownership in any software used for the DIPG Registry, by any Member or third party, the DIPG Registry shall be owned by the Members jointly. No Member can withdraw its Data from the DIPG Registry until the DIPG Registry is dissolved.

The results of the Project including the intellectual property rights thereto shall be owned by the Researcher generating the same. Intellectual project strategy shall be determined on a case by case basis, including possible compensation for commercial exploitation. The IPR strategy shall require prior assessment by and approval of the Executive Committee.

**8. Accountability**

The Executive Committee shall on an annual basis report to the Members the following subjects:

- 1. the number of approved and rejected Projects;
  2. any organisational problems;
  3. Funds and expenses;
  4. Other as agreed by the Members.

**9. Donor complaints procedure**

Complaints of Donors relating to or arising from the DIPG Registry shall be submitted to the institution of the Member at which the Donor is a patient and/or where Data were collected.

**10. About this document**

1. This Regulation may be referred to as follows: Regulatory Document of the SIOPE Diffuse Intrinsic Pontine Glioma (DIPG) Network, a sub-committee of the high-grade glioma (HGG) working group of the Brain Tumour Group (BTG) of the International Society of Paediatric Oncology Europe (SIOPE), version 1.1.
2. Copyright is vested in the Members jointly.

**APPENDIX A: MODEL CONFIDENTIALITY AGREEMENT**

**This Agreement is made by and between the** UNDERSIGNED:

Dutch Childhood Oncology Group, acting for and on behalf of the SIOPE Diffuse Intrinsic Pontine Glioma (DIPG) Network, having its principal place of business at, The Netherlands, lawfully represented in this matter by

Hereinafter referred to as “DCOG”,

and

, acting for itself and having an address at

Hereinafter referred to as “Advisor”

Hereinafter individually referred to as “Party” and collectively as “Parties”.

RECITALS

- The SIOPE Diffuse Intrinsic Pontine Glioma (DIPG) Network (hereinafter the “DIPG Network”) is a sub-committee of the high-grade glioma (HGG) working group of the Brain Tumour Group (BTG) of the International Society of Paediatric Oncology Europe (SIOPE) committed to supporting and fostering the mission of the Society;
- The DIPG Network is composed of paediatric oncologists, paediatric neurologists, radiotherapists, biologists, institutions and others motivated to carry out excellent clinical and biological research in the field of DIPG and collaborate with colleagues around the world;
- DCOG is the legal entity that on behalf of the Executive Committee of the DIPG Network is authorized to enter into third party agreements in matters concerning the DIPG Registry;
- The Advisor is an expert in the field of ; and
- The DIPG Network wishes to retain the Advisor to review certain neuro-images (the “Purpose”).

NOW, THEREFORE, in consideration of the advisor receiving Advisor information for the Purpose, which is of a confidential nature, the Parties hereby agree as follows:

1. For the purpose of this Confidentiality Agreement (the “Agreement”), "Confidential Information" shall mean information disclosed in whatever form by or on behalf of the DIPG Network and/or DCOG to Advisor under this Agreement, which information includes but is not limited to imaging materials, which imaging materials may include or be accompanied by personal data as defined in Directive 95/46/EC.

1. The Advisor in each case shall keep the Confidential Information in confiden­ce and will not use the same except for the Purpose. Accordingly, the Advisor will not publish or otherwise disclose the Confidential Information received without the prior written consent of the DIPG Network’s Executive Committee.
2. Notwithstanding clause 2 above, the Advisor may give access to the DIPG Network and/or DCOG’s Confidential Information to those employees or co-workers (the “Co-Advisors”) who have a need to know to such Confidential Information for the Purpose and who have been instructed of the restrictions imposed on the Advisor pursuant to this Agreement and who have agreed to abide by such restrictions. It is the responsibility of the Advisor to make sure that any and all Co-Advisors will be advised of and will abide to the conditions of this Agreement.
3. The Advisor’s obligations of confidentiality and non-disclosure pursuant to this Agreement shall however not apply to information and data:
4. which was already in possession of the Advisor at the time of receipt hereunder, as evidenced by their written re­cords; or
5. which was at the time of receipt or there­after becomes publicly available otherwise than in breach of any obligati­on he­reun­der; or
6. which the Advisor has received from a third party who is legally entitled to disclose the same; or
7. which was independently developed by employees of the Advisor without reference to the Confidential Information, as evidenced by the Advisor's written records; or
8. which is required by law, regulation or order of a competent authority (including any regulatory or governmental or securities exchange) to be disclosed.
9. The Advisor will maintain reasonable security with respect to said Confidential Information.
10. DCOG and the DIPG Network Executive Committee warrant that they have the rights to disclose the Confidential Information to the other Party.
11. Upon the completion of the Purpose or at the request of DCOG at any time during the term of this Agreement, the Advisor shall discontinue the use of and shall promptly return all Confidential Information and will promptly return all documentation and/or other information carriers embodying that Confidential Information.
12. This Agreement shall be effective from the date of final signature and terminates upon completion of the Purpose, provided that each Party may terminate this Agreement upon written notification to the other Party. The obligations of confidentiality shall survive the expiry or termination of this Agreement.
13. This Agreement shall be governed and enforced in accordance with the Laws of The Netherlands without regard to its conflict of law provisions. All disputes arising in connection with this Agreement shall exclusively be submitted to the competent courts in Den Haag, The Netherlands.

*[signatures on the next page]*

THUS AGREED UPON, SIGNED AND EXECUTED IN **TWO** COUNTERPARTS

**Dutch Childhood Oncology Group**

Signature: …………………………………… Signature: ……………………………………

Name: …………………………………… Name: ……………………………………

Title: …………………………………… Title: ……………………………………

Date: …………………………………… Date: ……………………………………

**APPENDIX B: CERTIFICATE OF AUTHORITY**

The Executive Committee of the SIOPE DIPG Network, pursuant to the “Regulatory Document of the SIOPE Diffuse Intrinsic Pontine Glioma (DIPG) Network, a sub-committee of the high-grade glioma (HGG) working group of the Brain Tumour Group (BTG) of the International Society of Paediatric Oncology Europe (SIOPE), version 1.1”, hereby authorises the Dutch Childhood Oncology Group (DCOG) to represent the SIOPE DIPG Network vis-à-vis [name and address contract party] and to negotiate and execute the [identify type of agreement] with respect to [add the purpose].

Subject to article 4.3 of the DIPG Regulatory Document, the Executive Committee of the SIOPE DIPG Network acknowledges and agrees that DCOG shall administer the funds received from [name of the contract party] and shall be entitled to make third party payments from such funds in accordance with the DIPG Network Regulatory Document. DCOG shall only pay such third party payments to the extent DIPG Network allocated funds are available at the DCOG bank accounts.

This Certificate of Authority is effective from [date] and will remain effective until the expiry or early termination of the [add type of agreement], whichever occurs first.

[Date]

...........................................................

Name: [name representative]

Title: Chair of the Executive Committee of the DIPG Network
