# Supplementary material for: Development of the SIOPE DIPG network, registry and imaging repository: a collaborative effort to optimize research into a rare and lethal disease
Source: J Neurooncol. 2017 Jan 21;132(2):255–66. doi: 10.1007/s11060-016-2363-y (PMC5378734; doi:10.1007/s11060-016-2363-y)
Supplement: Supplementary file 4 — Supplementary material 4 (DOC 67 KB) [file 11060_2016_2363_MOESM4_ESM.doc]

**Development of the SIOPE DIPG Network, Registry and Imaging Repository:**

**A collaborative effort to optimize research into a rare and lethal disease.**

Journal of Neuro-Oncology

*Sophie E.M. Veldhuijzen van Zanten, Joshua Baugh and Brooklyn Chaney, Dennis De Jongh, Esther Sanchez Aliaga, Frederik Barkhof, Johan Noltes, Ruben De Wolf, Jet Van Dijk, Antonio Cannarozzo, Carin M. Damen-Korbijn, Jan A. Lieverst, Niclas Colditz, Marion Hoffmann, Monika Warmuth-Metz, Brigitte Bison, David T.W. Jones, Dominik Sturm, Gerrit H. Gielen, Chris Jones, Esther Hulleman, Raphael Calmon, David Castel, Pascale Varlet, Géraldine Giraud, Irene Slavc, Stefaan Van Gool, Sandra Jacobs, Filip Jadrijevic-Cvrlje, David Sumerauer, Karsten Nysom, Virve Pentikainen, Sanna-Maria Kivivuori, Pierre Leblond, Natasha Entz-Werle, Andre O. von Bueren, Antonis Kattamis, Darren Hargrave, Péter Hauser, Miklos Garami, Halldora Kristin Thorarinsdottir, Jane Pears, Lorenza Gandola, Giedre Rutkauskiene, Geert O. Janssens, Ingrid K. Torsvik, Marta Perek-Polnik, Maria João Gil-da-Costa, Olga Zheludkova, Liudmila Shats, Ladislav Deak, Lidija Kitanovski, Ofelia Cruz, Andres Morales La Madrid, Stefan Holm, Nicolas Gerber, Rejin Kebudi, Richard Grundy, Enrique Lopez-Aguilar, Marta Zapata-Tarres, John Emmerik, Tim Hayden, Simon Bailey, Veronica Biassoni, Maura Massimino, Jacques Grill, William P. Vandertop, Gertjan J.L. Kaspers, Maryam Fouladi, Christof M. Kramm, Dannis G. van Vuurden on behalf of the members of the SIOPE DIPG Network.*

**Corresponsing author: Sophie E.M. Veldhuijzen van Zanten, VU University Medical Center Department of Pediatrics, Division of Oncology-Hematology, s.veldhuijzen@vumc.nl**

**PATIENT INFORMATION SHEETS**

SIOPE DIPG Registry

** PIS PARENTS **

City: ……………….

Date: ………………

Dear parent(s)/guardian,

In ……………….....(month/year), your son/daughter has been diagnosed with a diffuse intrinsic pontine glioma (DIPG). DIPG is a rare and childhood specific malignancy. Unfortunately there is no definite treatment, yet, to cure you child therefore we need common efforts to define and better treat this disease.

**Scientific research**

In order to get more insight into this disease and to answer questions such as: “How often does DIPG occur? and: “What are the effects of different treatments?”, paediatric oncologists have united within an international partnership: the SIOPE DIPG Network. The SIOPE DIPG Network is part of the SIOPE (International Society of Paediatric Oncology).

The SIOPE DIPG Network has, in collaboration with the Dutch Childhood Oncology Group (DCOG/SKION), developed a registration system for DIPG: the SIOPE DIPG Registry.

The aim of this registry study is to collect medical data from all DIPG patients diagnosed and treated in Europe. However, only data from parents and patients who give their permission to include the medical data anonymously will be collected. We therefore ask you whether you agree to include the data of your child, anonymously, in the SIOPE DIPG Registry.

**Advantages and disadvantages of participating in this study**

With the development of the SIOPE DIPG Registry, researchers will be able to perform epidemiologic studies and be able to evaluate how DIPG patients are treated and respond to treatment. These data are indispensable to improve the current treatment strategies and to evaluate whether applied changes truly improve the survival. Your child will probably have minimal or no benefit from participation in the SIOPE DIPG Registry, but his/her data may well contribute to better treatment and survival of future children with DIPG.

**What do we ask?**

We ask you whether you agree to include the medical data of your child anonymously in the SIOPE DIPG Registry. Participation in this study is a free choice and not an obligation. You have the right to refrain from recording information from your son/daughter in the SIOPE DIPG Registry at any time and without giving a reason. If you are currently under treatment, a decision not to participate or to stop recording will in no way affect the attention and care that your child receives.

**Confidentiality of data**

Your child's medical information is treated confidentially. Therefore, only authorized personnel will have access to the computer files. During registration, the confidentiality of the data is guaranteed by replacing the name of your child by a unique registration code. Only a few people in your local hospital know the key to this code.

If you refrain from further recording of information, the key to this code will be removed by authorized personnel in your local hospital. The already anonymized data will be kept in the SIOPE DIPG Registry.

Data from the SIOPE DIPG Registry will be used for research purposes only. When using the data for research, we will comply with the Data Protection Act.

**Grace period**

We advise you to take sufficient time to think about whether you want to give permission for participation in this registration. Also, you might want to talk about this with others. For this you get the opportunity and time.

**Permission**

If you decide to participate and to record the medical data of your son/daughter in the SIOPE DIPG Registry, we will ask you to sign an Informed Consent Form to confirm your consent. The treating physician will also sign the form. With doing this, he/she confirms that he/she has informed you about the registration system and that he/she was willing and available to answer emerging questions.

**Further information**

If, after reading this letter, before or during the period of registration, you have further questions, please feel free to contact your local hospital referent pediatric oncologist (Dr. ………………………………) or the researchers coordinating this project (via [info@dipgregistry.eu](mailto:info@dipgregistry.eu)).

Sincerely,

The paediatric oncologists united within the SIOPE DIPG Network

SIOPE DIPG Registry

** PIS DIPG PATIENTS **

City: ……………….

Date: ………………

Dear …………………,

In ………………........(month/year), you have been diagnosed with a diffuse intrinsic pontine glioma (DIPG). DIPG is a rare and childhood specific malignancy.

**Scientific research**

In order to get more insight into this disease and to answer questions such as: “How often does DIPG occur? and: “What are the effects of different treatments?”, paediatric oncologists have united within an international partnership: the SIOPE DIPG Network. The SIOPE DIPG Network is part of the SIOPE (International Society of Paediatric Oncology).

The SIOPE DIPG Network has, in collaboration with the Dutch Childhood Oncology Group (DCOG/SKION), developed a registration system for DIPG: the SIOPE DIPG Registry.

The aim of this registry study is to collect medical data from all DIPG patients diagnosed and treated in Europe. However, only data from patients and parents who give their permission to include the medical data anonymously will be collected. We therefore ask you whether you agree to include your medical data, anonymously, in the SIOPE DIPG Registry.

**Advantages and disadvantages of participating in this study**

With the development of the SIOPE DIPG Registry, researchers will be able to perform epidemiologic studies and be able to evaluate how DIPG patients are treated and respond to treatment. These data are indispensable to improve the current treatment strategies and to evaluate whether applied changes truly improve the survival. You will probably have minimal or no benefit from participation in the SIOPE DIPG Registry, but your data may well contribute to better treatment and survival of future children with DIPG.

**What do we ask?**

We ask you whether you agree to include your medical data anonymously in the SIOPE DIPG Registry.

Participation in this study is a free choice and not an obligation. You have the right to refrain from recording information in the SIOPE DIPG Registry at any time and without giving a reason. If you are currently under treatment, a decision not to participate or to stop recording will in no way affect the attention and care that you receive.

**Confidentiality of data**

Your medical information is treated with confidentially. Therefore, only authorized personnel will have access to the computer files. During registration, the confidentiality of the data is guaranteed by replacing your name by a unique registration code. Only a few people in your local hospital know the key to this code.

If you refrain from further recording of information, the key to this code will be removed by authorized personnel in your local hospital. The already anonymized data will be kept in the SIOPE DIPG Registry.

Data from the SIOPE DIPG Registry will be used for research purposes only. When using the data for research, we will comply with the Data Protection Act.

**Grace period**

We advise you to take sufficient time to think about whether you want to give permission for participation in this registration. Also, you might want to talk about this with others. For this you get the opportunity and time.

**Permission**

If you decide to participate and to record your medical data in the SIOPE DIPG Registry, we will ask you, and your parents, to sign an Informed Consent Form to confirm your consent. Your treating physician will also sign the form. With doing this, he/she confirms that he/she has informed you about the registration system and that he/she was willing and available to answer emerging questions.

**Further information**

If, after reading this letter, before or during the period of registration, you have further questions, please feel free to contact your local hospital referent pediatric oncologist (Dr. ………………………………) or the researchers coordinating this project (via [info@dipgregistry.eu](mailto:info@dipgregistry.eu)).

Sincerely,

The paediatric oncologists united within the SIOPE DIPG Network

Consent Form for Registration in the SIOPE DIPG Registry

(Medical file version)

I understand that the information about the disease and treatment of me/my son or daughter will be collected anonymously and will be recorded to the SIOPE DIPG Registry, managed by the Dutch Childhood Oncology Group (DCOG/SKION, The Netherlands).

It is clear to me that the data will be used for (inter)national studies into DIPG. The data are used to investigate the disease course, current treatments, and outcome. The research will be performed by members of the SIOPE DIPG Network only.

I understand that only authorized personnel has access to the (computer) files. The medical information of my (sons/daughters) disease will be used for no other purpose than the study of DIPG. In reports that follow from this research, I/my child will not be recognizable.

I know that I can ask for more information at all times. I know that the authorization for this registration can be withdrawn at any time. I know that I do not need to give a reason for this. Finally, it is clear to me that the care and quality of treatment does not change when I give my permission, consent or withdrawal to participate.

**Permission Statement:**

Patient Name (full): ……………………………………………………………………

Date of Birth: ……………………………………………………………………

Registry number: ……………………………………………………………………

**Parents:**

- I DO / DO NOT agree with the anonymous registration of medical data concerning the treatment and disease course of my son/daughter in the SIOPE DIPG Registry, managed by DCOG/SKION.

Name (full):

Parent/Guardian………………………………… Signature…………………………..... Date…………………………………

Parent/Guardian………………………………… Signature…………………………..... Date…………………………………

**Child (if applicable, given the age):**

- I DO / DO NOT agree with the anonymous registration of medical data concerning my treatment and disease course in the SIOPE DIPG Registry, managed by DCOG/SKION.

Name (full):

Patient……………..………………………………… Signature…………………………..... Date…………………………………

**Treating physician:**

- I certify that I have explained to the patient the content and purpose of the registration, as described above, and that I have given the patient and his/her parents the opportunity to ask questions.

Name (full):

Doctor……………..………………………………… Signature…………………………..... Date…………………………………

Consent Form for Registration in the SIOPE DIPG Registry

(Parent and patient version)

I understand that the information about the disease and treatment of me/my son or daughter will be collected anonymously and will be recorded to the SIOPE DIPG Registry, managed by the Dutch Childhood Oncology Group (DCOG/SKION, The Netherlands).

It is clear to me that the data will be used for (inter)national studies into DIPG. The data are used to investigate the disease course, current treatments, and outcome. The research will be performed by members of the SIOPE DIPG Network only.

I understand that only authorized personnel has access to the (computer) files. The medical information of my (sons/daughters) disease will be used for no other purpose than the study of DIPG. In reports that follow from this research, I/my child will not be recognizable.

I know that I can ask for more information at all times. I know that the authorization for this registration can be withdrawn at any time. I know that I do not need to give a reason for this. Finally, it is clear to me that the care and quality of treatment does not change when I give my permission, consent or withdrawal to participate.

**Permission Statement:**

Patient Name (full): ……………………………………………………………………

Date of Birth: ……………………………………………………………………

Registry number: ……………………………………………………………………

**Parents:**

- I DO / DO NOT agree with the anonymous registration of medical data concerning the treatment and disease course of my son/daughter in the SIOPE DIPG Registry, managed by DCOG/SKION.

Name (full):

Parent/Guardian………………………………… Signature…………………………..... Date…………………………………

Parent/Guardian………………………………… Signature…………………………..... Date…………………………………

**Child (if applicable, given the age):**

- I DO / DO NOT agree with the anonymous registration of medical data concerning my treatment and disease course in the SIOPE DIPG Registry, managed by DCOG/SKION.

Name (full):

Patient……………..………………………………… Signature…………………………..... Date…………………………………

**Treating physician:**

- I certify that I have explained to the patient the content and purpose of the registration, as described above, and that I have given the patient and his/her parents the opportunity to ask questions.

Name (full):

Doctor……………..………………………………… Signature…………………………..... Date…………………………………
